# Human cortex development is shaped by molecular and cellular brain systems

**DOI:** 10.1101/2023.05.05.539537

**Published:** 2023-05-05

**Authors:** Leon D. Lotter, Amin Saberi, Justine Y. Hansen, Bratislav Misic, Gareth J. Barker, Arun L. W. Bokde, Sylvane Desrivières, Herta Flor, Antoine Grigis, Hugh Garavan, Penny Gowland, Andreas Heinz, Rüdiger Brühl, Jean-Luc Martinot, Marie-Laure Paillère, Eric Artiges, Dimitri Papadopoulos Orfanos, Tomáš Paus, Luise Poustka, Sarah Hohmann, Juliane H. Fröhner, Michael N. Smolka, Nilakshi Vaidya, Henrik Walter, Robert Whelan, Gunter Schumann, IMAGEN Consortium, Frauke Nees, Tobias Banaschewski, Simon B. Eickhoff, Juergen Dukart

**Affiliations:** 1 Institute of Neuroscience and Medicine, Brain & Behaviour (INM-7), Research Centre Jülich, Germany; 2 Institute of Systems Neuroscience, Medical Faculty, Heinrich Heine University Düsseldorf, Germany; 3 Max Planck School of Cognition, Stephanstrasse 1A, 04103 Leipzig, Germany; 4 Otto Hahn Research Group for Cognitive Neurogenetics, Max Planck Institute for Human Cognitive and Brain Sciences, Leipzig, Germany; 5 McConnell Brain Imaging Centre, Montréal Neurological Institute, McGill University, Montréal, QC, Canada; 6 Department of Neuroimaging, Institute of Psychiatry, Psychology & Neuroscience, King’s College London, United Kingdom; 7 Discipline of Psychiatry, School of Medicine and Trinity College Institute of Neuroscience, Trinity College Dublin, Dublin, Ireland; 8 Centre for Population Neuroscience and Precision Medicine (PONS), Institute of Psychiatry, Psychology & Neuroscience, SGDP Centre, King’s College London, United Kingdom; 9 Institute of Cognitive and Clinical Neuroscience, Central Institute of Mental Health, Medical Faculty Mannheim, Heidelberg University, Square J5, Mannheim, Germany; 10 Department of Psychology, School of Social Sciences, University of Mannheim, 68131 Mannheim, Germany; 11 NeuroSpin, CEA, Université Paris-Saclay, F-91191 Gif-sur-Yvette, France; 12 Departments of Psychiatry and Psychology, University of Vermont, 05405 Burlington, Vermont, USA; 13 Sir Peter Mansfield Imaging Centre School of Physics and Astronomy, University of Nottingham, University Park, Nottingham, United Kingdom; 14 Department of Psychiatry and Psychotherapy CCM, Charité – Universitätsmedizin Berlin, corporate member of Freie Universität Berlin, Humboldt-Universität zu Berlin, and Berlin Institute of Health, Berlin, Germany; 15 Physikalisch-Technische Bundesanstalt (PTB), Braunschweig and Berlin, Germany; 16 Ecole Normale Supérieure Paris-Saclay, Université Paris-Saclay, Université paris Cité, INSERM U1299 “Trajectoires Développementales & Psychiatrie”; Centre Borelli CNRS UMR9010, Gif-sur-Yvette, France; 17 AP-HP Sorbonne Université, Department of Child and Adolescent Psychiatry, Pitié-Salpêtrière Hospital, Paris; 18 Department of Psychiatry, EPS Barthélémy Durand, Etampes, France; 19 Departments of Psychiatry and Neuroscience, Faculty of Medicine and Centre Hospitalier Universitaire Sainte-Justine, University of Montreal, Montreal, Quebec, Canada; 20 Departments of Psychiatry and Psychology, University of Toronto, Toronto, Ontario, Canada; 21 Department of Child and Adolescent Psychiatry and Psychotherapy, University Medical Centre Göttingen, von-Siebold-Str. 5, 37075, Göttingen, Germany; 22 Department of Child and Adolescent Psychiatry and Psychotherapy, Central Institute of Mental Health, Medical Faculty Mannheim, Heidelberg University, Square J5, 68159 Mannheim, Germany; 23 Department of Psychiatry and Neuroimaging Center, Technische Universität Dresden, Dresden, Germany; 24 Centre for Population Neuroscience and Stratified Medicine (PONS), Department of Psychiatry and Neuroscience, Charité Universitätsmedizin Berlin, Germany; 25 School of Psychology and Global Brain Health Institute, Trinity College Dublin, Ireland; 26 Centre for Population Neuroscience and Precision Medicine (PONS), Institute for Science and Technology of Brain-inspired Intelligence (ISTBI), Fudan University, Shanghai, China; 27 Institute of Medical Psychology and Medical Sociology, University Medical Center Schleswig-Holstein, Kiel University, Kiel, Germany

**Keywords:** Neurodevelopment, Cortex, Cortical thickness, Normative modeling, Nuclear imaging, Neurotransmitters, Neuronal cell types, Dominance analysis, Longitudinal

## Abstract

Human brain morphology undergoes complex developmental changes with diverse regional trajectories. Various biological factors influence cortical thickness development, but human data are scarce. Building on methodological advances in neuroimaging of large cohorts, we show that population-based developmental trajectories of cortical thickness unfold along patterns of molecular and cellular brain organization. During childhood and adolescence, distributions of dopaminergic receptors, inhibitory neurons, glial cell populations as well as features of brain metabolism explain up to 50% of variance associated with regional cortical thickness trajectories. Cortical maturation patterns in later life are best explained by distributions of cholinergic and glutamatergic systems. These observations are validated in longitudinal data from over 8,000 adolescents, explaining up to 59% of developmental change at population- and 18% at single-subject level. Integrating multilevel brain atlases with normative modeling and population neuroimaging provides a biologically and clinically meaningful path to understand typical and atypical brain development in living humans.

## 1 Introduction

Human cerebral cortex volume and thickness are subject to global and regionally specific developmental changes^1,2^. Myelination, synaptic remodeling (“pruning”), as well as neuronal and glial reorganization have been suggested as key biological factors influencing development and maturation of cortical thickness (CT)^3–6^. More recently, availability of human high-resolution postmortem transcriptomic brain atlases^7^ enabled the study of cortex-wide colocalization patterns between CT estimates and genetic markers of microstructural tissue properties^3,8^. These studies point to involvement of genes associated with glial cells (astrocytes, microglia, oligodendrocytes), pyramidal neurons, and neuronal cell components (myelination, dendrites, dendritic spines) in CT development^9–14^. However, biological processes underlying CT development as observed using magnetic resonance imaging (MRI), are likely not constrained to the cellular level. Instead, brain morphological changes may reflect development and/or activity of broader brain-functional processes such as brain metabolism, immunity, as well as molecular signaling, e.g., neurotransmitter functions.

The rising popularity of multimodal association techniques is paralleled by recent progress in population-level normative modeling of brain development^15^. Used as a reference norm, these models have great value as tools for quantification of individual pathophysiology^16^. However, such reference models also carry the potential to study developmental trajectories as far as these models represent typical brain development in “representative subjects” with high temporal resolution. Combining these models with human *postmortem* cell population markers^17^ and recently published collections of diverse *in vivo* nuclear imaging atlases mapping various neurotransmitter, brain metabolism, and molecular systems^18–24^ can provide new insights into mechanisms underlying CT development. Going beyond *typical* development, neurodevelopmental disorders are associated with both, atypical cortex development^25–27^ and dysfunction of various neurotransmitter systems^28,29^. However, given a lack of reproducible biomarkers^30^, methodological approaches that describe and bridge to the molecular level are urgently called for^31^.

Assuming that CT changes over the lifespan are shaped by development, maturation, and degeneration of certain biological systems, we hypothesized that the spatiotemporal patterns of CT development colocalize with the non-pathological adult spatial distributions of the respective biological systems^10,13^. Increased colocalization of CT changes with an individual biological system at a given developmental period would then support a role of the associated biological process in respective developmental alterations. Establishing these spatial associations will help to identify both typical and atypical human neurodevelopmental mechanisms, supporting formulation of hypotheses for future research endeavors. If translated to the level of the individual subject, spatial colocalization estimates promise value as interpretable biomarkers of biological and clinical significance^18,32,33^.

**Fig. 1:**
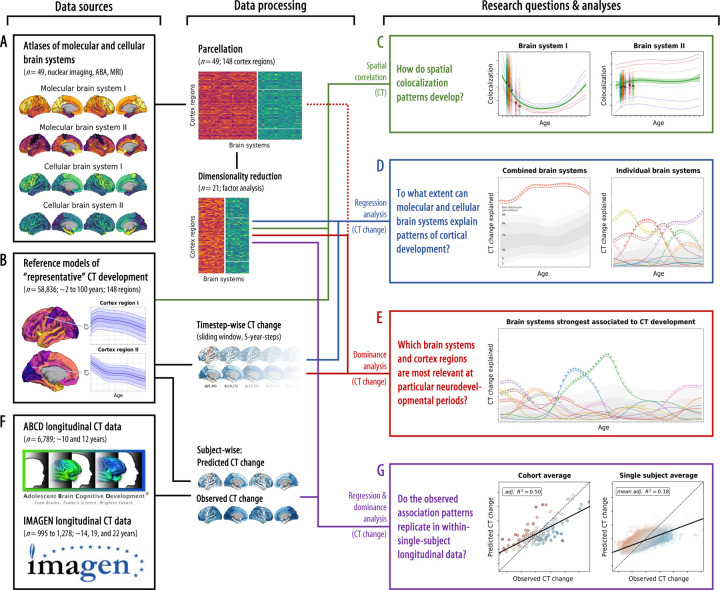
Study overview The workflow of the present study, from data sources (left side) to data processing and analysis method (middle) to the research questions and results (right side). A–G: See last Introduction paragraph. Abbreviations: CT = cortical thickness, ABA = Allen Brain Atlas, MRI = magnetic resonance imaging.

Following this reasoning, we demonstrate that population-average and single-subject CT trajectories colocalize with, and are explained by, spatial distributions of brain metabolism and immunity features, neurotransmitter systems, cortical myelin, as well as neuronal and glial cell populations. We gathered 49 *in vivo* and *postmortem* brain atlases mapping these neurobiological systems and extracted the latent spatial patterns using factor analysis (Fig. 1; A). Population-average CT trajectories for 148 cortex regions were obtained from a normative model by Rutherford et al.^2^ based on over 58,000 subjects (B). In the following, we refer to the clustered brain atlases as “(multilevel) biological (brain) systems”, and to the Rutherford CT data as “representative” or “modeled CT (change)”. First, we tested if modeled CT at each given time point in life was distributed across the cortex in patterns reflecting the distributions of specific biological brain systems (C)^13^. Temporal dynamics in these spatial colocalization patterns can provide first evidence on which systems are associated with cortical development. To further understand the observed developmental associations, we fitted regression models predicting the spatial patterns of modeled longitudinal CT *change* from biological brain systems. The outcome was quantified as the overall and system-wise explained variance *R*^*2*^, interpretable as the percentage to which CT change patterns can be explained from multilevel systems^19,34^. We first assessed the combined and individual relevance of the biological brain systems for cortical development (D). After identifying the most important systems, we evaluated their role in explaining modeled CT changes while accounting for shared variance (E). Last, we validated the observed model-based CT development associations using longitudinal CT data from approximately 8,000 adolescents (F)^35,36^, successfully validating our findings on the individual level (G).

## 2 Results

### 2.1 Multilevel biological brain systems

Using factor analyses, we extracted the latent spatial patterns underlying the 49 included brain atlases to reduce predictor intercorrelation in the following regression analyses. To independently evaluate the relevance of molecular- and cellular-level systems for explaining CT development, this step was performed separately per atlas modality resulting in 10 nuclear imaging (named *ni1–10*), 10 gene expression cell markers (*ce1–10*), and an MRI-derived myelin map (*mr1*; Supp. 1.1; Fig. S1–2; Tab. S1). Each original atlas was assigned to the biological system (factor-level map) on which it loaded most strongly, and the factor maps were named accordingly (Fig. S2D–E). The factor maps represented biologically meaningful brain systems, with the first factor capturing the first spatial component of cortical transmitter systems (*ni1*), followed by more specific factors broadly representing serotonergic *(ni2)*, dopaminergic (*ni3, ni9*), and cholinergic systems (*ni5*) as well as brain metabolism (*ni4, ni6*) and immunity (*ni7, ni10*). Similarly, mRNA expression-derived factors entailed one general neuronal dimension (*ce1*) and several more specific excitatory and inhibitory neuronal (*ce4–10*) and glial factors (*ce2–3*).

### 2.2 Cross-sectional colocalization patterns show distinct lifespan trajectories

**Fig. 2:**
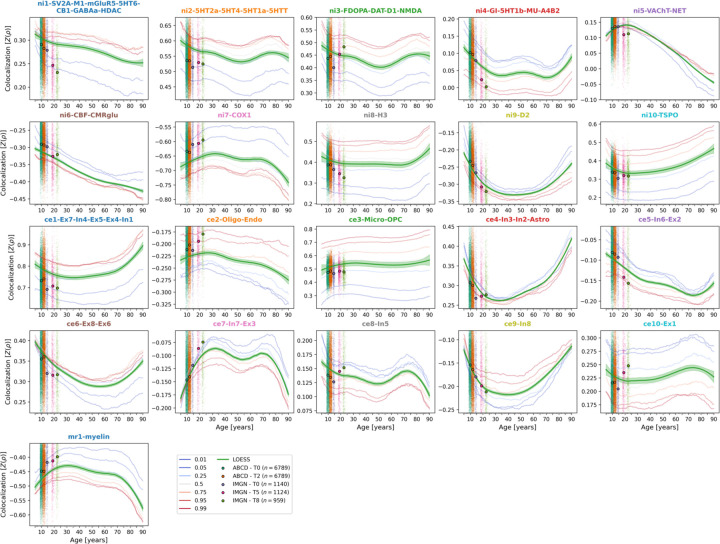
Colocalization between cross-sectional representative CT and multilevel brain systems across the lifespan Lifespan trajectories of colocalization between multilevel biological brain systems and cross-sectional CT. Z-transformed Spearman correlation coefficients are shown on the y axis, age on the x axis. Blue-to-red lines: percentiles of extracted modeled CT data (see legend). Note that these do not show actual percentiles of colocalization strengths. Green: LOESS line smoothed through the percentile data to highlight trajectories. Scatters: individual subjects from ABCD and IMAGEN cohorts at each timepoint with mean colocalization strength indicated by the larger dots. These serve to validate the observations based on modeled CT (i.e., strength and sign of the colocalizations). Abbreviations: CT = cortical thickness, MRI = magnetic resonance imaging, LOESS = locally estimated scatterplot smoothing, SV2A = synaptic vesicle glycoprotein 2A, M1 = muscarinic receptor 1, mGluR5 = metabotropic glutamate receptor 5, 5HT1a/1b/2a/4/6 = serotonin receptor 1a/2a/4/6, CB = cannabinoid receptor 1, GABAa = γ-aminobutyric acid receptor A, HDAC = histone deacetylase, 5HTT = serotonin transporter, FDOPA = fluorodopa, DAT = dopamine transporter, D1/2 = dopamine receptor 1/2, NMDA = N-methyl-D-aspartate glutamate receptor, GI = glycolytic index, MU = mu opioid receptor, A4B2 = α4β2 nicotinic receptor, VAChT = vesicular acetylcholine transporter, NET = noradrenaline transporter, CBF = cerebral blood flow, CMRglu = cerebral metabolic rate of glucose, COX1 = cyclooxygenase 1, H3 = histamine receptor 3, TSPO = translocator protein, Ex = excitatory neurons, In = inhibitory neurons, Oligo = oligodendrocytes, Endo = endothelial cells, Micro = microglia, OPC = oligodendrocyte progenitor cells, Astro = astrocytes.

First, we asked if CT patterns across cortex regions colocalized with spatial distributions of multilevel brain systems and how these colocalization patterns developed over the lifespan. After extraction of representative CT data from 5 to 90 years of age^2^ (Supp. 1.2; age distribution: Fig. S3; CT trajectories: S4A, Anim. S1), spatial Spearman correlation analyses with biological brain systems revealed diverse colocalization trajectories with a general pattern of strongest changes in early and late phases of life (Fig. 2). Colocalization strengths varied across the centiles of modeled CT, but temporal trajectories were consistent. Sex did not relevantly influence the trajectories (Fig. S5).

### 2.3 Lifespan CT development is explained by multilevel biological systems

Studying population-level and individual brain development inevitably requires looking at respective changes over time, rather than focusing only on cross-sectional data^37^. In that respect, we tested to which extent biological systems explained the *relative change* of representative CT across the lifespan and which systems showed the strongest associations. For each cortex region, relative CT *changes* over time were computed using a sliding window approach (5-year window length; Fig. S4B–C). Using linear regression models and strict permutation testing, we asked if the spatial patterns of CT changes over time were explained by multilevel brain systems, both combined (multivariate) and individually (univariate). To provide an estimate of overparameterization effects in multivariate analyses, regression analysis results are shown in relation to their respective permuted models.

**Fig. 3:**
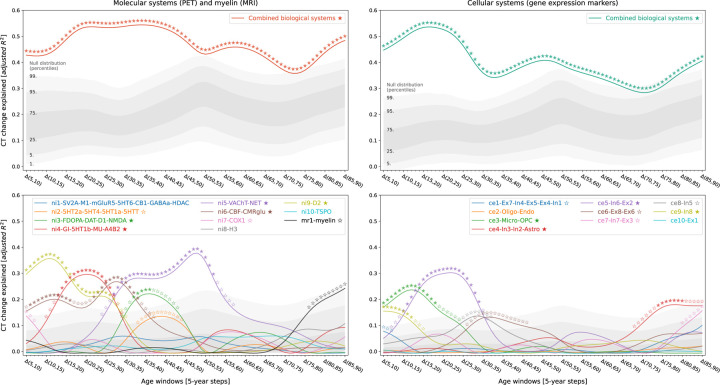
Representative lifespan CT change patterns explained by multilevel biological systems Associations between modeled lifespan CT change and multilevel brain systems separated by data sources (left vs. right). Colored lines show the amount of spatial CT change variance explained (y axis) by the combined biological systems (upper) or each system individually (lower) throughout the lifespan (x axis). Stars indicate significance of each regression model estimated with a permutation-based approach; filled: FDR-corrected across all tests shown in each panel of the plot; empty: nominal *p* < 0.05. To provide an estimate of the actual observed effect size, gray areas show the distributions of spatial CT change variance explained by permuted predictor maps (n = 10,000). For the lower panel, null results were combined across predictor maps. Abbreviations: CT = cortical thickness, PET = positron emission tomography, MRI = magnetic resonance imaging, FDR = false discovery rate, see Fig. 2 for abbreviations used in biological system names.

The combined biological systems at molecular and cellular levels explained up to 54% of the spatial variance in representative CT changes across the lifespan with peaks at about 20–35 (molecular) and 15–20 (cellular) years of age, respectively [false discovery-rate (FDR)-corrected; Fig. 3]. In univariate analyses, 9 of the 21 multilevel systems including major neurotransmitter systems (dopaminergic, cholinergic, noradrenergic), features of brain metabolism, neuron populations, as well as glia cells displayed significant associations with CT changes during at least one timestep. These systems explained 15–38% of CT change patterns with most systems showing peaks between 5 and 30 years of age (Fig. 3). Combining all 21 systems across biological levels explained up to 67% of CT changes at 15–25 years of age (Fig. S6). All findings were robust to changes in sliding window step size, modeled sex, and CT percentile (Fig. S6–7).

### 2.4 Distributions of dopaminergic neurotransmitter systems, glia cells, and inhibitory neurons account for the majority of explained CT development

Next, we focused on the 9 multilevel biological systems that best explained CT change patterns in univariate analyses (FDR-corrected). We sought to understand in detail how specific systems contributed to the overall explained CT change, while accounting for intercorrelation and shared spatial variance patterns between molecular and cellular levels. In the above analyses, we found the strongest cortical changes and subsequently the strongest associations with the multilevel brain system during the main neurodevelopmental period from childhood to young adulthood. Because of the particular relevance of this timespan also from a clinical viewpoint, we included CT change from 5 to 30 years as an additional time window for further testing.

**Fig. 4:**
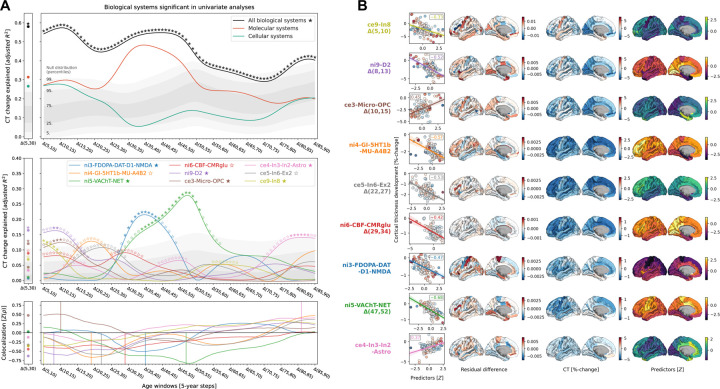
In-depth analysis of the molecular and cellular systems most relevant for explaining representative CT change patterns across the lifespan A: Modeled lifespan CT change explained by multilevel brain systems. See Fig. 3 for descriptions of global plot elements. Top: overall explained CT change variance, the two colored lines highlight contributions of molecular and cellular systems. Middle: System-wise contributions to the overall explained spatial variance. Note that, as the used total dominance statistic describes the average *R*^*2*^ associated with each predictor relative to the “full model” *R*^*2*^, the sum of the predictor-wise values at each timepoint in the middle plot equals the *R*^*2*^ values expressed in the upper panel. Bottom: Spearman correlations between CT change and multilevel brain systems to visualize the sign of the association patterns. See Fig. 3 for further details. B: Cortex-regional influences on explained CT change. Each row shows one of the 9 brain systems included in dominance analyses. Scatterplots: Correlation between CT change at the respective predictor’s peak timestep (y axis) and the predictor map, corresponding to panel A-bottom. The first brains show the residual difference maps calculated for each multilevel system. For illustration purposes, the second and third brains show CT change and the spatial distribution associated with the system. See Fig. S8 for all residual difference maps, Fig. S4C for all CT change maps, and Fig. S2G for all predictor maps. Abbreviations: CT = cortical thickness, see Fig. 2 for abbreviations used in biological system names.

The spatial distributions of the 9 selected molecular and cellular brain systems jointly explained 58% of CT changes from 5 to 30 years, peaking at 10–15 years of age (Fig. 4A, top). Using dominance analyses, we tested for the relative importance of these predictors, while accounting for shared variance^19,34^. All 9 brain systems contributed to the overall explained CT change during different life periods (nominal *p* < 0.05) with 6 systems surviving FDR-correction (Fig. 4A, middle; Anim. S2). During the main neurodevelopmental period, 3 of these 6 systems explained most of the spatial CT change patterns, representing estimates of dopaminergic receptors (*ni9-D2*; *R*^*2*^ = 16%; peek at 8–14 years; D2 = dopamine receptor 2), microglia and oligodendrocyte progenitor cells (*ce3-Micro-OPC*; *R*^*2*^ = 12%; 8–15 years; Micro = microglia, OPC = oligodendrocyte progenitor cells), as well as of somatostatin-expressing interneurons (*ce8-In8*; *R*^*2*^ = 12%; 5–14 years). Midlife CT maturation patterns were explained by 2 systems most strongly associated with dopaminergic and cholinergic neurotransmission (*ni3-FDOPA-DAT-D1-NMDA* and *ni5-VAChT-NET*; 29–56 years; FDOPA = fluorodopa, DAT = dopamine transporter, D1 = dopamine receptor 1, NMDA = N-methyl-D-aspartate glutamate receptor; VAChT = vesicular acetylcholine transporter, NET = noradrenaline transporter). Finally, late-life CT aging patterns were associated with a system mostly representing inhibitory neuron populations (*ce4-In3-In2-Astro*, 78–88 years; In = inhibitory neuron, Astro = astrocytes). Except for microglia and oligodendrocyte progenitor cells, all identified associations were negative, i.e., indicating a *stronger reduction* of CT in areas with *higher density* of the respective biological system.

#### 2.4.1 Medial occipitotemporal, sensorimotor, and cingulate cortices drive CT associations

All results reported here arise from the colocalization of whole-brain spatial patterns between CT data and biological brain systems. These spatial associations are likely dominated by some cortical regions relative to others. To identify regions specific to each biological system, we evaluated each system’s effect on the region-wise prediction errors of every multivariate model (one per each timestep). We found the most influential regions to be generally located in medial occipital, medial temporal, sensorimotor, and cingulate cortices (Supp. 1.3; Fig. 4B; Fig S8; Anim. S2).

#### 2.4.2 Factor-level associations transfer to original molecular and cellular brain atlases

Our focus on the latent spatial patterns underlying the original 49 brain atlases reduced predictor intercorrelation and increased statistical power. However, aiding interpretation and confirming validity of the factor-level systems, the original atlases that were most closely related to each factor-level system explained modeled CT change patterns to similar extents (Supp. 1.4; Fig. S9). We found further associations between *ni9-D2* and the D1 dopaminergic receptor as well as between *ni5-VAChT-NET* and the α4β2 nicotinic receptor. Indeed, for the latter, NET was of lesser importance, while the factor *ni3-FDOPA-DAT-D1-NMDA* was dominated by the NMDA receptor and the factor *ce3-Micro-OPC* was dominated by the microglia distribution. Residual regional differences showed similar patterns as observed with factor-level systems, with additional relevance of the medial frontal gyrus for the microglia atlas and lateral temporal gyri for D1/D2 receptors (Fig. S10).

### 2.5 Single-subject longitudinal data confirms the normative model-based findings

Next, we evaluated if the general approach and the model-based findings translated to single-subject data. A successful validation on the individual level would provide further evidence for the potential mechanistic relevance of the associated brain systems and support the use of normative reference models to non-invasively study subject-specific neurodevelopmental mechanisms. Considering the high relevance of the first life decades from both neurodevelopmental and clinical standpoints, we focused these analyses on childhood and adolescence.

Summarizing the model-based results pertaining to this period of life, we found that 6 biological systems explained over 50% of early life cortical development. D1/2 dopaminergic receptors, microglia, and somatostatin-expressing interneurons were most relevant during this time period, with medial and lateral temporal, medial occipital, and cingulate cortices being the most influential cortex regions. Transitioning to the individual level and independent data, we then assessed the extent to which these 6 brain systems explain (i) cohort-average and (ii) single-subject CT changes at different ages in two longitudinal neurodevelopmental cohorts. 6,789 subjects from the ABCD study^35^ were scanned at ~10 and 12 years of age and 985–1177 subjects from the IMAGEN cohort^36^ were scanned at ~14, 19, and 22 years (demographics and quality control: Supp. 1.5, Tab. S2, Fig. S11; Observed-vs.-predicted CT change patterns and correlations: Fig. S12–S13; Effects of site on CT and CT change: Tab. S3–4). Of note, ABCD baseline data (~10 years), but not ABCD follow-up data and the IMAGEN cohort, were included in the estimation of the Rutherford et al.^2^ normative model. We contrasted our findings to those obtained from individual-level normative CT predicted from each subject’s age and sex using the Rutherford et al. model^2^.

**Fig. 5:**
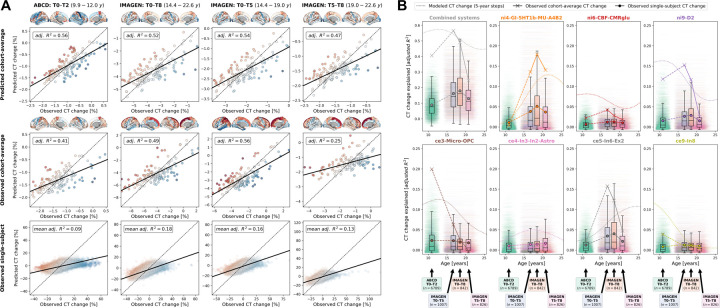
Validation of model-based results in ABCD and IMAGEN datasets A: Explained spatial CT change variance in ABCD and IMAGEN data. The overall model performance is illustrated as scatter plots contrasting predicted CT change (y axis) with observed CT change (x axis). Scatters: single brain regions, color-coded by prediction error. Continuous line: linear regression fit through the observations. Dashed line: theoretical optimal fit. Brains: prediction errors corresponding to scatters. Rows: upper = cohort-average predicted by the reference (“Braincharts”) model; middle: observed cohort-average; lower: observed single-subject values, one regression model was calculated for each subject, but the results were combined for illustration purposes. B: Explained spatial CT change variance with a focus on the individual multilevel systems. Subplots for the combined analysis and each individual multilevel system show: modeled CT change as presented in Fig. 4 (dotted line); observed cohort-average CT change (cross markers); and observed single-subject CT change (boxplots and dot markers). For each subject, one horizontal line at their individual *R*^*2*^ value ranges from their age at beginning and end of each time span. Boxplots show the distribution of individual values for each time span. Note that the first subplot (“Combined systems”) corresponds to the data presented in panel A. See Fig. S16–17 for detailed results. Abbreviations: CT = cortical thickness, adj. = adjusted, see Fig. 2 for abbreviations used in biological system names.

First, we confirmed that the colocalization between cross-sectional single-subject CT and biological systems mirrored the patterns observed for the modeled population-average (Fig. 2 & S14). Next, we evaluated the longitudinal CT change between study time points, looking at the 10–12 years timespan for ABCD and 14–22, 14–19, and 19–22 years timespans for IMAGEN. The 6 brain systems explained cohort-average CT changes to extents comparable with the reference model (minimum/maximum observed *R*^*2*^ = 25/56%, model-prediction *R*^*2*^ = 47/56%; Fig. 5A upper and center). These patterns translated to the single-subject level, explaining on average between 9 and 18% in individual CT changes with considerable variability (range *R*^*2*^ = 0–59%; Fig. 5A, lower; Fig. S15). Looking at system-wise contributions, we again found the model-based patterns to be reflected on both cohort-average and single-subject levels (Fig. 5B & Fig. S15–16). While the brain systems predicted to be most important (D1/2 and microglia) indeed explained significant amounts of CT change, two other systems, which primarily reflected aerobic glycolysis (*ni4*) and granule neurons (*ce5*), were equally dominant.

Finally, we tested if the regression models estimated to predict each subject’s *normative* CT change patterns generalized to each subject’s *observed* change patterns, i.e., we asked if a “one-size-fits-all” approach would have performed equally well. While these models on average indeed exceeded the predictive performance of permuted null models, they did not provide good fit for many individuals, thus highlighting the value of our individual differences-focused approach (Fig. S17). Additional sensitivity analyses showed that the explained CT changes (i) were not relevantly influenced by the reference model-based site-adjustment (Fig. S15), (ii) increased with longer follow-up duration within each time period (Fig. S18), (iii) varied by sex and study site in some tested time periods (Supp. 1.6; Fig. S19), and (iv) varied with individual atypical CT development as well as data quality (Supp. 1.7; Fig. S20).

## 3 Discussion

**Fig. 6:**
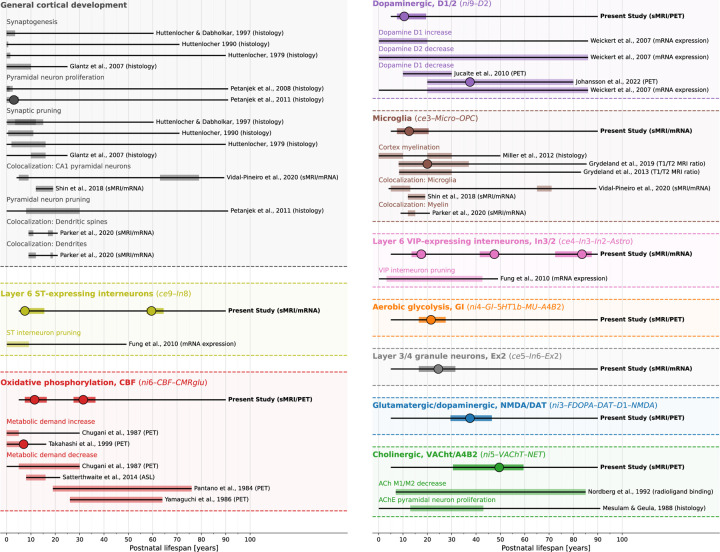
Summary of study findings in the context of prior literature Condensed visualization of the reported results (first line of each block) in context with related results of previous human studies investigating similar biological systems (below lines). Each header indicates one biological system or process, each thin black line overlaid by a colored bar indicates results from one study. If a study reported multiple results within the same system (e.g., from two different brain regions), bars were laid over each other. Thin black lines: overall time span investigated. Colored overlay: time period in which significant associations to CT (change) patterns were reported (nominal *p* < 0.05), independent of the sign of the association. Large dots: Timepoint of the maximum association. See also Fig. S21 and Tab. S5 for a more comprehensive overview including various topics. Abbreviations: ST = somatostatin, CR = calretinin, sMRI = structural MRI, CBF = cerebral blood flow, PET = positron emission tomography, ASL = arterial spin labeling, ACh(E) = acetylcholine (esterase), see Fig. 2 for abbreviations used in biological system names.

Factors shaping human brain morphology over the life span are poorly understood. Here we demonstrate that the complex patterns in which the human cerebral cortex develops and matures colocalize with specific biological systems on molecular and cellular levels. Our findings support roles of the dopaminergic system, microglia, somatostatin-expressing interneurons, brain metabolism, and granule neurons in early CT development, whereas cholinergic and dopaminergic neurotransmission are associated with CT changes across adulthood (Fig. 6). Our results not only have implications for the study of typical neurodevelopment, but also hold promise for the value of neurodevelopmental cross-modal association analyses for future clinical research applications.

We find the colocalization between developmental changes of cortex morphology and corresponding adult-derived neurotransmitter and cell type profiles to closely reflect neurodevelopmental processes across various biological systems (see Fig. 6, Fig. S21, and Tab. S5 for a descriptive overview). For example, while synaptogenesis and neuronal and glial proliferation continue into the first postnatal years, the second and third life decades are marked by a targeted reduction of neurons and cell components, likely reflecting functional specialization^38–46^. In line with our findings, dopamine D1 receptor activity was reported to peak in adolescence and young adulthood before declining steadily with age^47–49^. Considering the observed impact of the dopaminergic system on cortical development, this association warrants further study in context with dopaminergic pathomechanisms of neurodevelopmental disorders^50,51^. Our findings concerning somatostatin-expressing interneurons and microglia fit with prior reports showing somatostatin interneuron markers to decrease strongly within the first decade of life^46^ and microglial involvement in synaptic remodeling^4,12,13^ as well as myelination^52,53^, which has been shown to continue into the fourth decade of life^10,11,54–56^. Approaching adulthood, cortical development becomes less dynamic with most regions taking stable or steadily decreasing aging trajectories^1,2^. We find these phases to be reflected in spatial colocalization patterns in that most biological systems colocalize with CT changes in early cortex development. Only the cholinergic system consistently predicts cortical changes throughout adulthood, potentially pointing to its role in healthy and pathological aging^57^. Considering the lack of early biomarkers of accelerated aging this association warrants further investigation^58^.

Normative modeling of large-scale neuroimaging data has received considerable attention as a tool to translate research into clinical applications^1,2,16,59^. We show that if used as a reference for typical brain development, combining normative models of brain regional features with spatial colocalization approaches can facilitate discovery of physiological mechanisms underlying specific conditions. Going beyond this group-level discovery approach, we demonstrate the feasibility of neurodevelopmental spatial colocalization analyses in single subjects by mapping individual-level deviations from normative trajectories to specific biological systems. In view of this ability of molecular and cellular brain systems to explain *typical* developmental and maturational patterns of the cortex, studying how these findings translate to *atypical* neurodevelopment^60^ may be a fruitful path for future research. From a clinical perspective, mapping deviating brain developmental patterns to potentially underlying biological processes promises value not only for biomarker discovery but also for identification of therapeutic targets to correct deviations from normative trajectories.

Importantly, spatial association analyses as applied here do not impose causality and, thus, the reported associations only provide indirect evidence for involvement of specific biological systems in cortical development and therewith guidance to future causal studies of specific processes. Our analyses are also limited by the heterogeneity of the brain atlases which were derived from independent adult populations of varying age and sex, processed with different strategies, and in part derived from postmortem data. Similar restrictions apply to the normative CT model which is largely based on the WEIRD (Western, Educated, Industrialized, Rich, and Democratic)^61^ population^2,62^. The contribution of these factors needs to be quantified in future research. Nonetheless, the high replicability of the observed associations, despite the noise introduced by both limitations, rather strengthens the robustness of our findings.

To summarize, patterns of spatial colocalization between macroscale brain structure and its underlying brain-organizational levels provide *in vivo* insight into healthy and pathological processes that are otherwise difficult or impossible to study. This approach has already provided valuable information on typical neurodevelopment^3^ and pathophysiological mechanisms underlying neurological and psychiatric disorders^19,34,63,64^. The now occurring transition to the individual level allows to place these biologically interpretable MRI markers in their broader system-level context, ranging from genetics and epigenetics to behavior and psychopathology^18,32,33^.

## 4 Method

### 4.1 Overview

Multi-level brain atlases were retrieved from open sources, parcellated, and dimensionality reduced. Modeled CT (change) data were extracted from the Rutherford et al.^2^ reference model (from here on termed “Braincharts” model) and ABCD/IMAGEN datasets. First, we assessed the trajectories of colocalization between modeled *cross-sectional* cortex-wide CT patterns and multi-level brain systems. We then tested how these systems explain modeled *longitudinal* CT change patterns jointly and individually in multivariate and univariate regression frameworks. To account for spatial variance shared between molecular and cellular levels, and to distinguish the specific contributions of multilevel systems to explaining CT change, we submitted the systems showing the strongest univariate associations to dominance and brain-regional analyses. To validate the model-based results and transfer them to the single-subject level, we tested if cohort- and individual-level CT development in the ABCD and IMAGEN samples was explained by specific biological systems as predicted from our model-based findings.

### 4.2 Software

Multimodal brain atlases were retrieved and processed from/with *neuromaps*^20^, *abagen*^65^, *JuSpace*^18^, or author sources. Analyses of associations between CT and cortical atlases were conducted using *JuSpyce 0.0.2*^66^ in a *Python 3.9.11* environment (Lotter and Dukart, 2022). *JuSpyce* (https://github.com/LeonDLotter/JuSpyce) is a toolbox allowing for flexible assessment and significance testing of associations between multimodal neuroimaging data, relying on imaging space transformations from *neuromaps*^20^, brain surrogate map generation from *brainSMASH*^67^, and several routines from *Nilearn*^68^, *scipy*^69^, *NiMARE*^70^, *statsmodels*, *numpy*, and *pandas*. Visualizations were created using *matplotlib*^71^, *seaborn*^72^, and *surfplot*^73^. The *PCNtoolkit*^74,75^ was used to generate modeled CT data, as well as site-adjusted/predicted CT data and deviation scores for ABCD and IMAGEN subjects.

### 4.3 Ethics

No new human data were acquired for this study. Ethical approval for collection and sharing of the used human neuroimaging and behavioral data (brain atlases, Braincharts model, ABCD and IMAGEN datasets) were provided by local ethics committees who reviewed the original projects. Detailed information is available in the cited sources. Use of the ABCD data is registered at the NDA database at http://dx.doi.org/10.15154/1528657. The responsible IMAGEN investigator is T. Banaschewski.

### 4.4 Data sources and processing

#### 4.4.1 Atlases of multi-level brain systems

Multi-level brain atlases (Fig. S1) were separated into two broad categories according to their source modality. Sample characteristics and data sources are provided in Tab. S1.

The *neuroimaging* (“*ni-*”) dataset consisted of group-average nuclear imaging atlases (neurotransmitter receptors, brain metabolism and immunity, synaptic density, and transcriptomic activity) and an MRI-based myelin atlas^18–23^. Maps were (i) transformed from fsLR (metabolism and myelin maps) or Montreal Neurological Institute space (all others) to fsaverage5 space using registration fusion^20,76^, (ii) parcellated in 74 cortical regions per hemisphere^77^, and (iii) *Z*-standardized across parcels within each atlas.

*Cell type* (“*ce-*”) atlases were built by (i) retrieving genetic cell type markers identified by Lake et al.^17^ via single-nucleus RNA sequencing in human brain tissue from the PsychENCODE dataset^78^, (ii) extracting Allen Human Brain Atlas mRNA expression values^7^ for each Destrieux parcel and each marker gene using *abagen*^65^ (default settings, data mirrored across hemispheres, Supp. 2.1), (iii) *Z*-standardizing the data across parcels within each gene, and (iv) taking the uniform average of the data across genes within each cell type.

We reduced the dimensionality of the atlas datasets to decrease multicollinearity in multivariate regression analyses. As the nuclear imaging and mRNA expression data likely differed strongly in terms of confounds and signal-to-noise ratio, and to study molecular- and cellular-level effects separately, data sources were not mixed during dimensionality reduction. To retain interpretability, we used factor analysis for dimensionality reduction (minimum residuals, promax rotation). All unrotated factors that explained ≥ 1% of variance of each dataset were retained. We chose the oblique rotation method as the resulting factor intercorrelation would be expected from non-independent biological systems. Resulting predictors were named by assigning each original atlas to the factor it loaded on the most (nuclear imaging: *ni1–n*; mRNA expression: *ce1–n*; MRI, only the myelin atlas, no dimensionality reduction: *mr1*).

#### 4.4.2 Braincharts CT model

The *Braincharts* reference model was estimated on 58,836 subjects from 82 sites (50% training/testing split; 51% female; age range 2.1–100 years; age distribution: Fig. S3). Detailed information on the included samples, CT estimation, and modeling procedure was provided by Rutherford et al.^2^. Notably, while ABCD baseline data were included in the model estimation, ABCD follow-up and IMAGEN data were not. Briefly, T1-weighted MRI data were obtained from the original cohorts and FreeSurfer 6.0^79^ was used to extract parcel-wise CT data. Image quality was ensured based on FreeSurfer’s Euler characteristic^80^ and manual quality control of 24,354 images^35^. CT development was modeled separately for each Destrieux parcel using warped Bayesian linear regression models predicting CT from age, sex, and site as fixed effect. The applied methodology was developed for use in large datasets, can model nonlinear and non-Gaussian effects, accurately accounts for confounds in multisite datasets, and allows for estimation of site batch effects in previously unseen data^2,81–83^.

We extracted Braincharts CT data separately for females and males for each of 148 cortical parcels for 171 timepoints (5–90 years with 0.5-year steps) and 7 percentiles (1^st^, 5^th^, 25^th^, 50^th^, 75^th^, 95^th^, and 99^th^). We focused on CT data from the age of 5 years onwards as the used FreeSurfer pipeline was not adjusted for very young ages^2^. For colocalization analyses, the extracted modeled CT data were used as is. For model-based (pseudo-)longitudinal analyses, we calculated the relative CT change *∆CT* from year *i* to year *j* based on the median (50^th^ percentile) sex-average CT data as $\text{Δ}CT_{\left(i,j\right)}=\frac{CT_{j}-CT_{i}}{CT_{i}}$. Lifespan CT change was then calculated using a sliding window with 1-year steps and 5-year length from 5 to 90 years as Δ*CT*_(*i*,*j*)_, *i* ∈ [5..85], *j* = *i* + 5.

#### 4.4.3 ABCD and IMAGEN CT data

Processed and parcellated CT data from the Adolescent Brain Cognitive Development (ABCD) cohort^35^ was taken directly from the ABCD 4.0 release. Baseline (T0, ~10 years) and 2-year follow-up (T2) structural MRI data were processed using FreeSurfer 7.1.1. Details were provided by Casey et al.^35^ and in the release manual (http://dx.doi.org/10.15154/1523041). For the IMAGEN cohort^36^, T1-weighted MRI data at baseline (T0, ~14 years) and at one or two follow-up scans (T5, ~19, and T8, ~22 years) were retrieved and processed with FreeSurfer’s standard pipeline (7.1.1). Following Rutherford et al.^2^, for quality control we relied on an Euler-like metric, i.e., the total number of surface defects as provided by FreeSurfer. We excluded subjects that exceeded a threshold of 𝑄3 + 𝐼𝑄𝑅 × 1.5 calculated in each sample across timepoints^80,84^ or failed the manual quality ratings provided in the ABCD dataset. One ABCD study site (MSSM) stopped data collection during baseline assessment and was excluded. We utilized the Braincharts model to harmonize CT data of the two datasets across sites (ABCD: n = 20; IMAGEN: n = 8) and to derive individual deviation scores to be used only in sensitivity analyses. Site effects were estimated in healthy subsamples of both dataset’s baseline data (n = 20 per site, 50% female) distributed evenly across baseline age ranges. These subjects including their follow-up data, and all subjects with data for less than two study time points, were excluded from further analyses. As the ABCD baseline data were used in training the Braincharts model, we conducted sensitivity analyses on the non-adjusted data to estimate potential overfitting effects.

Colocalization analyses were calculated on the site-adjusted and original CT values at each timepoint. For longitudinal analyses, the relative CT change between each time point within each cohort was calculated as above (ABCD: T0–T2; IMAGEN: T0–T8, T0–T5, and T5–T8).

### 4.5 Null map-based significance testing

Spatial associations between brain maps can be assessed in correlative analyses in the sense of testing for cortex- or brain-wide alignment of the distributions of two variables A (e.g., CT) and B (e.g., a neurotransmitter receptor)^10,18,19,85^. Effect sizes (e.g., correlation coefficients) resulting from correlating A and B convey interpretable meaning. However, parametric *p* values do not, as they are influenced by the rather arbitrary number of “observations” (between thousands of voxels/vertices and a few parcels) and spatial autocorrelation in the brain data^86^. Null model-based inference approaches circumvent this problem by comparing the observed effect size to a null distribution of effect sizes obtained by correlating the original brain map A with a set of permuted brain maps generated from B to derive empirical *p* values. From several approaches proposed to preserve or reintroduce spatial autocorrelation patterns in the null maps^86^, we relied on the variogram-based method by Burt et al.^67^ as implemented in *JuSpyce* via *BrainSMASH*^20,66,67^.

### 4.6 Discovery analyses based on the Braincharts model

#### 4.6.1 Lifespan colocalization trajectories

To characterize lifespan trajectories of colocalization between cross-sectional CT and multi-level brain systems, we calculated Spearman correlations between each brain atlases and modeled CT data at each extracted time point and percentile. Smoothed regression lines (locally estimated scatterplot smoothing) were estimated on data from all percentiles combined to highlight developmental trajectories. As the resulting developmental patterns were largely similar across sexes, we performed the main analyses on female-male averaged CT data and reported sex-wise results in the supplementary materials.

#### 4.6.2 Prediction of CT change

The main objective of this study was to determine the extent and the temporal patterns to/in which multi-level brain systems could explain CT development and lifespan change. To achieve this goal, we designed a framework in which we “predicted” stepwise relative CT change from one or more brain atlases in multivariate or univariate regression analyses. The amount of CT variance explained *R*^*2*^ was used as the main outcome measure (adjusted in multivariate analyses). Exact one-sided *p* values were calculated by generating a constant set of 10,000 null maps for each multi-level brain atlas and comparing observed *R*^*2*^ values to *R*^*2*^ null distributions obtained from 10,000 regression analyses using the null maps as predictors.

To determine the general extent to which CT development could be explained, we performed one multilinear regression per lifespan timestep (81 models) using (i) all neuroimaging and (ii) all mRNA expression-based atlases. In an additional analysis, we assessed the result combining all atlases irrespective of modality. The resulting *p* values were FDR-corrected across all models separately per modality. To quantify individual atlas-wise effects and identify specific biological systems of potential relevance to CT development, we performed univariate regression analyses per timestep and atlas (21 × 81 models), again correcting for multiple comparisons using FDR correction within each modality. In sensitivity analyses, we assessed the effects of CT percentile (1^st^ and 99^th^), sex (female and male separately), and window length (1-year, 2-year). As above, the results were consistent across sexes, thus all main analyses were reported for sex-average CT data and the following model-based analyses were performed only on sex-average data.

#### 4.6.3 System-wise contributions to explained CT change

Aiming to identify when and how biological systems contributed to explaining CT change, we retained only those brain atlases that significantly explained CT development individually (FDR correction) and conducted dominance analyses “predicting” CT change from this joint set of atlases. Dominance analysis aims to quantify the relative importance of each predictor in a multivariate regression. The *total dominance* statistic is calculated as the average contribution of a predictor *x* to the total *R*^*2*^ across all possible subset models of a multivariate regression and can here be interpreted as the extent to which CT development during a certain time period is explained by *x* in presence of the whole set of predictors *X* and as a fraction of the extent to which CT development is explained by set *X*^19,34,87^. Following from this, in our models, the sum of the atlas-level *R*^*2*^ at a given timespan equals the total *R*^*2*^ at this time point. Significance of dominance analyses was determined as described above by generating null distributions and estimating empirical *p* values for both, the “full model” multivariate *R*^*2*^ and the predictor-wise total dominance *R*^*2*^. Finally, Spearman correlations between CT change and multi-level brain atlases were conducted to indicate the directionality of associations.

Dominance analyses were conducted at each timestep and, to highlight the main postnatal developmental period between child and adulthood, on the CT development across this entire period defined as Δ*CT*_(5,30)_ (82 models). Resulting *p* values were corrected across the whole analysis (full model and atlas-wise: 82 + 82 × 9 *p* values).

#### 4.6.4 Brain-regional influences on CT change association patterns

To estimate the importance of individual brain regions for the associations between CT change and brain atlases, we relied on the atlas-wise *residual differences* across brain-regions as unitless measures of the influence of individual cortex regions on the dominance analysis results. The residual difference of prediction errors Δ*PE* for each predictor *x* out of the predictor set *X* was calculated as $\text{Δ}PE=\left|PE_{X\backslash \left\{x\right\}}\right|-\left|PE_{X}\right|$. The results were visualized on surface maps for descriptive interpretation.

#### 4.6.5 Relationships between dimensionality-reduced and original multi-level atlases

Assessing whether the factor-level atlases represented the original multi-level atlases according to the applied atlas-factor-association scheme, we performed dominance analyses per *factor-level atlas* using the strongest associated *original atlases* as predictors. The latter were defined as the five atlases that loaded the most on each factor if the absolute loading exceeded 0.3. FDR correction was performed across all models per factor-level atlas.

### 4.7 Validation analyses based on ABCD and IMAGEN single-subject data

#### 4.7.1 Developmental colocalization trajectories

First, we tested whether colocalization patterns between multi-level atlases and single-subject cross-sectional CT followed the predictions of the Braincharts model. Spearman correlations were calculated between each subject’s CT values and each atlas at all available timepoints, for both site-adjusted CT data and the data prior to site-effect-correction.

#### 4.7.2 Explained CT development patterns on cohort- and single-subject levels

Following, we assessed how the brain systems that significantly explained modeled CT development during the period covered by ABCD and IMAGEN data (9–25 years) performed in single-subject longitudinal data. Dominance analyses were performed in two steps. First, for each of the four investigated time spans (ABCD: ~10–12; IMAGEN: ~14–22, ~14–19, 19–22 years), one dominance analysis was calculated to predict the *cohort-average* CT change pattern from multi-level brain systems. Second, dominance analyses were calculated in the same fashion, but *for every subject*. For comparison, analyses were repeated on CT change patterns as predicted by the Braincharts model from each subject’s age and sex. For cohort-average dominance analyses, exact *p* values were estimated as described for the stepwise model-based analyses. For individual-level analyses, instead of estimating p values for each subject, we tested whether the mean *R*^*2*^ values of the full models and each predictor observed in each cohort and time span were significantly higher than was estimated in 1,000 null-analyses with permuted atlas data. Finally, we repeated subject-level analyses on the original CT change data prior to site-effect-correction and on the longitudinal change of deviation Z scores as returned by the Braincharts model^2^.

Finally, we evaluated how the subject-level regression models predicting CT change patterns from biological systems generalized from the subject-level normative CT change patterns to the actual observed CT change. For that, we applied the regression model parameters estimated on each subject’s normative CT change patterns to each subject’s observed CT change and evaluated model fit as the subject-level correlation between predicted and observed CT change. To estimate the effect size, results were contrasted to null analyses in which each regression model was estimated using 1,000 permuted multilevel brain maps. Further sensitivity analyses were conducted to estimate how CT change predictions were affected by follow-up duration, sex, study site, “normativity” of CT and CT change patterns [correlation between predicted and observed CT (change), average Braincharts CT deviation (change), count of extreme deviation (change)], and data quality (number of surface defects). Subject-level full model *R*^*2*^ values were compared by sex and study site using analyses of covariances corrected for follow-up duration (and sex). All other variables were correlated with full model *R*^*2*^ values using Spearman correlations.

## 5 Data availability

All scripts and data supporting our analyses are available in a GitHub repository (https://github.com/LeonDLotter/CTdev/), except for original data and derivatives from the ABCD and IMAGEN datasets that cannot be shared openly (https://abcdstudy.org/; https://imagen-project.org/). The Braincharts model is available from: https://github.com/predictive-clinical-neuroscience/braincharts.

## Supplementary Material

Supplement 1

Supplement 2

Supplement 3

Supplement 4
