# Human cortex development is shaped by molecular and cellular brain systems

**DOI:** 10.1101/2023.05.05.539537

**Published:** 2023-10-04

**Authors:** Leon D. Lotter, Amin Saberi, Justine Y. Hansen, Bratislav Misic, Casey Paquola, Gareth J. Barker, Arun L. W. Bokde, Sylvane Desrivières, Herta Flor, Antoine Grigis, Hugh Garavan, Penny Gowland, Andreas Heinz, Rüdiger Brühl, Jean-Luc Martinot, Marie-Laure Paillère, Eric Artiges, Dimitri Papadopoulos Orfanos, Tomáš Paus, Luise Poustka, Sarah Hohmann, Juliane H. Fröhner, Michael N. Smolka, Nilakshi Vaidya, Henrik Walter, Robert Whelan, Gunter Schumann, IMAGEN Consortium, Frauke Nees, Tobias Banaschewski, Simon B. Eickhoff, Juergen Dukart

**Affiliations:** 1 Institute of Neuroscience and Medicine, Brain & Behaviour (INM-7), Research Centre Jülich; Jülich, Germany.; 2 Institute of Systems Neuroscience, Medical Faculty, Heinrich Heine University; Düsseldorf, Germany.; 3 Max Planck School of Cognition; Stephanstrasse 1A, 04103 Leipzig, Germany.; 4 Otto Hahn Research Group for Cognitive Neurogenetics, Max Planck Institute for Human Cognitive and Brain Sciences; Leipzig, Germany.; 5 McConnell Brain Imaging Centre, Montréal Neurological Institute, McGill University; Montréal, QC, Canada.; 6 Department of Neuroimaging, Institute of Psychiatry, Psychology & Neuroscience, King’s College London; London, United Kingdom.; 7 Discipline of Psychiatry, School of Medicine and Trinity College Institute of Neuroscience, Trinity College Dublin; Dublin, Ireland.; 8 Centre for Population Neuroscience and Precision Medicine (PONS), Institute of Psychiatry, Psychology & Neuroscience, SGDP Centre, King’s College London; London, United Kingdom.; 9 Institute of Cognitive and Clinical Neuroscience, Central Institute of Mental Health, Medical Faculty Mannheim, Heidelberg University; Square J5, Mannheim, Germany.; 10 Department of Psychology, School of Social Sciences, University of Mannheim; 68131 Mannheim, Germany.; 11 NeuroSpin, CEA, Université Paris-Saclay; F-91191 Gif-sur-Yvette, France.; 12 Departments of Psychiatry and Psychology, University of Vermont; 05405 Burlington, Vermont, USA.; 13 Sir Peter Mansfield Imaging Centre School of Physics and Astronomy, University of Nottingham; University Park, Nottingham, United Kingdom.; 14 Department of Psychiatry and Psychotherapy CCM, Charité – Universitätsmedizin Berlin, corporate member of Freie Universität Berlin, Humboldt-Universität zu Berlin, and Berlin Institute of Health; Berlin, Germany.; 15 Physikalisch-Technische Bundesanstalt (PTB); Braunschweig and Berlin, Germany.; 16 Ecole Normale Supérieure Paris-Saclay, Université Paris-Saclay, Université paris Cité, INSERM U1299 “Trajectoires Développementales & Psychiatrie”; Centre Borelli CNRS UMR9010, Gif-sur-Yvette, France.; 17 AP-HP Sorbonne Université, Department of Child and Adolescent Psychiatry, Pitié-Salpêtrière Hospital; Paris, France.; 18 Department of Psychiatry, EPS Barthélémy Durand; Etampes, France.; 19 Departments of Psychiatry and Neuroscience, Faculty of Medicine and Centre Hospitalier Universitaire Sainte-Justine, University of Montreal; Montréal, QC, Canada.; 20 Departments of Psychiatry and Psychology, University of Toronto; Toronto, Ontario, Canada.; 21 Department of Child and Adolescent Psychiatry and Psychotherapy, University Medical Centre Göttingen; von-Siebold-Str. 5, 37075, Göttingen, Germany.; 22 Department of Child and Adolescent Psychiatry and Psychotherapy, Central Institute of Mental Health, Medical Faculty Mannheim, Heidelberg University; Square J5, 68159 Mannheim, Germany.; 23 Department of Psychiatry and Neuroimaging Center, Technische Universität Dresden; Dresden, Germany.; 24 Centre for Population Neuroscience and Stratified Medicine (PONS), Department of Psychiatry and Neuroscience, Charité Universitätsmedizin Berlin; Berlin, Germany.; 25 School of Psychology and Global Brain Health Institute, Trinity College Dublin; Dublin, Ireland.; 26 Centre for Population Neuroscience and Precision Medicine (PONS), Institute for Science and Technology of Brain-inspired Intelligence (ISTBI), Fudan University; Shanghai, China.; 27 Institute of Medical Psychology and Medical Sociology, University Medical Center Schleswig-Holstein, Kiel University; Kiel, Germany.

**Keywords:** Neurodevelopment, Cortex, Cortical thickness, Normative modeling, Longitudinal, Nuclear imaging, Neurotransmitters, Neuronal cell types, Dominance analysis

## Abstract

Human brain morphology undergoes complex changes over the lifespan. Despite recent progress in tracking brain development via normative models, current knowledge of underlying biological mechanisms is highly limited. We demonstrate that human cerebral cortex development unfolds along patterns of molecular and cellular brain organization, traceable from population-level to individual developmental trajectories. During childhood and adolescence, cortex-wide spatial distributions of dopaminergic receptors, inhibitory neurons, glial cell populations, and brain-metabolic features explain up to 50% of variance associated with regional cortical thickness trajectories. Adult cortical change patterns are best explained by cholinergic and glutamatergic neurotransmission. These relationships are supported by developmental gene expression trajectories and translate to longitudinal data from over 8,000 adolescents, explaining up to 59% of developmental change at population- and 18% at single-subject level. Integrating multilevel brain atlases with normative modeling and population neuroimaging provides a biologically meaningful path to understand typical and atypical brain development in living humans.

## 1. Introduction

The human cerebral cortex develops in complex patterns^1–3^, giving rise to our cognitive abilities^4^. Biologically, these morphological changes are likely driven by developmental processes on different organizational levels. Cellular processes, e.g., myelination, synaptic remodeling (“pruning”), as well as neuronal and glial proliferation, have been discussed as the main drivers of cortical thickness (CT) development^5–8^. However, the mechanisms that drive lifetime CT changes might extend beyond cellular densities to more functional properties, such as metabolism, immune responses, and neurotransmitter signaling. Crucially, cellular densities and functional properties – hereafter collectively referred to as “(multilevel) neurobiological markers” – are not uniform across the cortex, but show distinct spatial distributions^9,10^. Assuming that CT changes over the lifespan are shaped by activity, development, or degeneration of cell populations and molecular processes, it is to be hypothesized that the spatiotemporal patterns of CT development colocalize with non-pathological adult spatial distributions of the respective neurobiological marker^11,12^. Increased colocalization of CT changes with an individual marker at a given developmental period would then support a role of the associated cell population or process in respective CT changes^5,13^. Supporting this notion, spatial patterns of CT development as measured with magnetic resonance imaging (MRI) are correlated with adult distributions of glial cells, pyramidal neurons, and neuronal cell components^11,12,14–17^. To derive cortical atlases of neural cell populations or their components, prior research has mostly relied on postmortem gene expression data from the Allen Brain Atlas^18^. However, such ex vivo data may only poorly represent the in vivo expression patterns for many genes^19^.

Neurodevelopmental disorders are associated with both deviations in brain structure^20–22^ and dysfunction of several neurotransmitter systems^23,24^, but suffer from a lack of reproducible biomarkers and little clinical translation of neuroimaging research^25^. It stands to reason that a better understanding of the processes underlying typical neurodevelopment can also shed light on potential neuropathology. Recently published large-scale normative models of human structural brain development^1,3^ represent an important step in the study of both population-level typical and individual atypical neurodevelopment^26,27^. Similarly, our understanding of brain organization was significantly advanced by the availability of modern in vivo nuclear imaging atlases^28,29^, explaining disordered brain structure to greater extents as compared to MRI-based brain structural and functional metrics^30^.

**Fig. 1:**
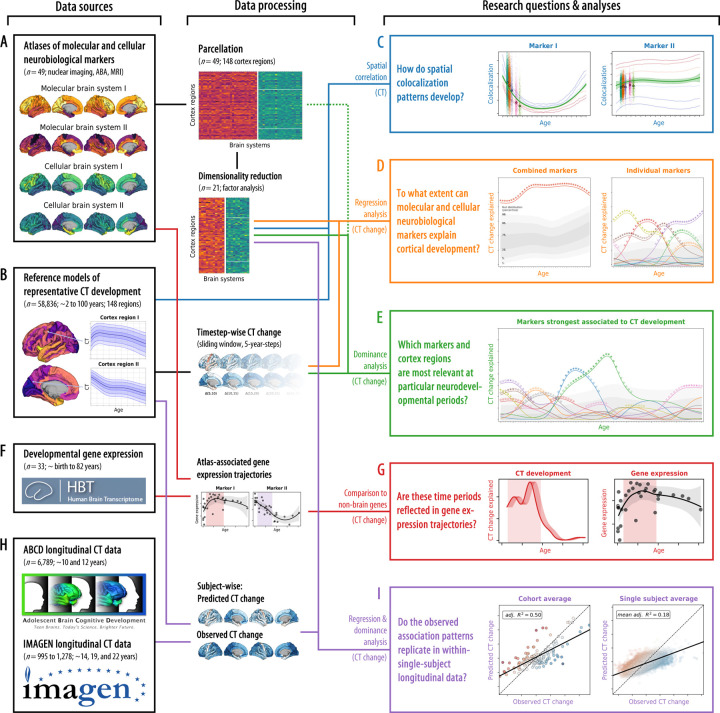
Study overview The workflow of the present study, from data sources (left side) to data processing and analysis method (middle) to the research questions and results (right side). **(A)** A collection of postmortem cellular and in vivo molecular brain atlases was parcellated and dimensionality reduced. **(B)** “Representative” population-median CT data was extracted from a normative model. **(C)** We calculated the colocalization between multilevel neurobiological markers and CT at each point throughout the lifespan. **(D)** We evaluated how combined and individual neurobiological markers could explain lifespan CT change. **(E)** The strongest associated markers were examined in detail, accounting for shared spatial patterns. **(F)** A developmental gene expression dataset was used to generate trajectories of gene expression associated with each multilevel neurobiological marker. **(G)** Periods in which CT change was significantly explained were validated in developmental gene expression data. **(H)** Single-subject longitudinal data was extracted from two developmental cohorts. **(I)** Findings based on the normative model were validated in single-subject data. Abbreviations: CT = cortical thickness, ABA = Allen Brain Atlas, MRI = magnetic resonance imaging.

Our current knowledge on biological factors that guide typical human cortex development is severely limited by practical obstacles. Yet this knowledge is necessary to understand atypical neurodevelopment and develop targeted biomarkers and treatments. Multimodal neuroimaging-based spatial association approaches can provide a window into specific biological mechanisms, but until now were limited to postmortem data and the cellular level. Combining these approaches with to date’s availability of large-scale normative models and *in vivo* derived molecular brain atlases constitutes the next major step in the imaging-based study of human brain development (Fig. 1). If translated to the level of the individual subject, it can serve as the foundation for future neuroimaging-based yet biologically interpretable biomarkers to be tested for their clinical potential^28,31,32^.

## 2. Results

### 2.1. Mapping neurobiological markers to cortical development

**Fig. 2:**
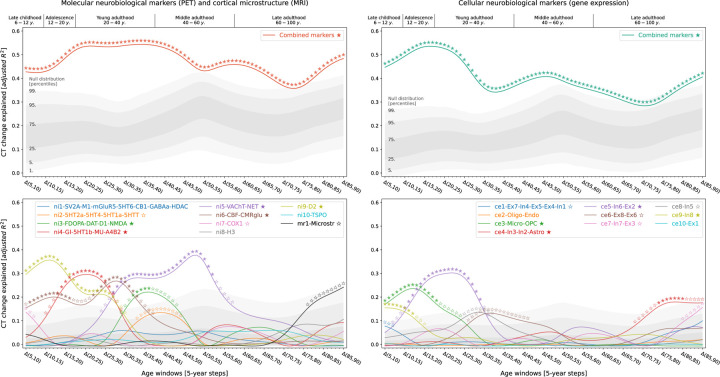
Modeled lifespan CT change patterns explained by multilevel neurobiological markers Associations between modeled lifespan CT change and neurobiological markers separated by data sources **(left vs. right)**. Developmental periods covered by this study as defined by Kang et al. are shown on top. Time periods were aligned to the center of each CT change step (e.g., Δ(5,10) = 7.5). **Colored lines** show the amount of spatial CT change variance explained **(y axis)** by the combined markers **(upper)** or each marker individually **(lower)** throughout the lifespan **(x axis)**. **Stars** indicate significance of each regression model estimated with a permutation-based approach; ★: FDR-corrected across all tests shown in each panel of the plot; ☆: nominal *p* < 0.05. To provide an estimate of the actual observed effect size, **gray areas** show the distributions of spatial CT change variance explained by permuted marker maps (*n* = 10,000). For the lower panel, null results were combined across marker maps. See Fig. S4C for all CT change maps, and Ext. Data Fig. 2 for all predictor maps. **Abbreviations**: CT = cortical thickness, PET = positron emission tomography, MRI = magnetic resonance imaging, FDR = false discovery rate, SV2A = synaptic vesicle glycoprotein 2A, M1 = muscarinic receptor 1, mGluR5 = metabotropic glutamate receptor 5, 5HT1a/1b/2a/4/6 = serotonin receptor 1a/2a/4/6, CB = cannabinoid receptor 1, GABAa = γ-aminobutyric acid receptor A, HDAC = histone deacetylase, 5HTT = serotonin transporter, FDOPA = fluorodopa, DAT = dopamine transporter, D1/2 = dopamine receptor 1/2, NMDA = N-methyl-D-aspartate glutamate receptor, GI = glycolytic index, MU = mu opioid receptor, A4B2 = α4β2 nicotinic receptor, VAChT = vesicular acetylcholine transporter, NET = noradrenaline transporter, CBF = cerebral blood flow, CMRglu = cerebral metabolic rate of glucose, COX1 = cyclooxygenase 1, H3 = histamine receptor 3, TSPO = translocator protein, Microstr = cortical microstructure, Ex = excitatory neurons, In = inhibitory neurons, Oligo = oligodendrocytes, Endo = endothelial cells, Micro = microglia, OPC = oligodendrocyte progenitor cells, Astro = astrocytes.

In this work, we explored if and to what extent cortical distributions of neural cell populations and molecular processes can explain spatiotemporal patterns of CT change throughout the lifespan. For this, we gathered 49 postmortem and in vivo brain atlases mapping neuronal and glial cell populations^33,34,18^, cortical microstructure^29^, as well as neurotransmitter receptors, brain metabolism and immunity^28,29,35^ in independent healthy adult samples (Fig. 1A; sources and processing: Ext. Data Tab. 1, Suppl. Text S1.1.1 and 2; surface plots: Ext. Data Fig. 1; temporal stability during adulthood: Text S1.1.3 and Fig. S1). The analytic approach taken here establishes associations between temporospatial CT (change) patterns and brain atlases based on the similarity of whole-brain spatial patterns. Intercorrelation arising from spatial patterns shared between atlases on either cellular or molecular levels was reduced by factor analyses applied independently to cellular and molecular markers (see Methods). This process resulted in 10 “factor-level” nuclear imaging maps (*ni1–10*), 10 gene expression cell marker maps (*ce1–10*), and an MRI-derived marker of cortical microstructure (T1/T2 ratio; *mr1*) which were named based on the most closely related original atlases (Ext. Data Fig. 2; Text S1.1.4; Fig. S2). Factor solutions were successfully validated against permuted brain maps (Text S1.1.5). These factor-level maps represented biologically meaningful entities with the first factor capturing the first spatial component of cortical transmitter systems (*ni1*), followed by more specific factors broadly representing serotonergic *(ni2)*, dopaminergic (*ni3, ni9*), and cholinergic systems (*ni5*) as well as brain metabolism (*ni4, ni6*) and immunity (*ni7, ni10*). Similarly, mRNA expression-derived factors entailed one general neuronal dimension (*ce1*) and several more specific excitatory and inhibitory neuronal (*ce4–10*) and glial factors (*ce2–3*).

In the following, we report on how these multilevel neurobiological markers colocalize and explain CT change patterns between 5 and 90 years of age (Fig 1B), spanning developmental periods from “middle and late childhood” to “late adulthood” as defined previously^9,1^ (see following Figs.). CT trajectories for 148 cortex regions were derived from a normative model of CT development^3^ estimated from over 58,000 subjects (referred to as “representative” or “modeled” CT; Fig. 1B; Text S1.2; age distribution: Fig. S3; CT trajectories: Fig. S4A and Animation S1).

First, we tested if representative *cross-sectional* CT at each given time point in life was distributed across the cortex in patterns reflecting the distributions of specific neurobiological markers (Fig. 1C)^12^. To further understand the observed developmental associations, we fitted regression models predicting the spatial patterns of modeled longitudinal CT *change* from neurobiological markers. The outcome was quantified as the overall and marker-wise explained variance *R*^*2*^, interpretable as the percentage to which CT change patterns can be explained from multilevel markers^29,30^. We first assessed the combined and individual relevance of all 21 neurobiological markers for cortical development (D). After identifying a subset of important markers, we evaluated their role in explaining modeled CT changes while accounting for shared variance (E). Next, we utilized developmental gene expression data (F) to validate and further specify our imaging-based findings (G). Last, we transferred our approach to longitudinal CT data from approximately 8,000 adolescents^36,37^ (H) to demonstrate that time period-specific association patterns identified using the normative model translate to the individual subject level (I).

### 2.2. Cross-sectional CT shows diverse colocalization trajectories

**Fig. 3:**
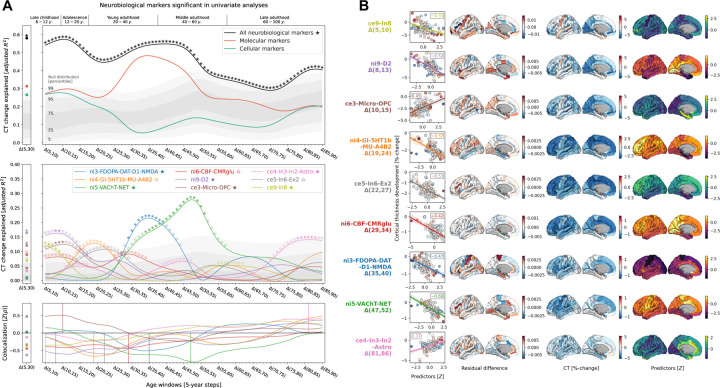
In-depth analysis of the neurobiological markers most relevant for explaining modeled CT change patterns across the lifespan **(A)** Modeled lifespan CT change explained by multilevel neurobiological markers. See Fig. 2 for descriptions of global plot elements. **Top**: overall explained CT change variance, the two colored lines highlight contributions of molecular and cellular markers. **Middle**: Marker-wise contributions to the overall explained spatial variance. Note that, as the used total dominance statistic describes the average *R*^*2*^ associated with each predictor relative to the “full model” *R*^*2*^, the sum of the predictor-wise values at each timepoint in the middle plot equals the *R*^*2*^ values expressed in the upper panel. **Bottom**: Spearman correlations between CT change and multilevel markers to visualize the sign of the association patterns. **(B)** Regional influences on explained CT change. **Each row** shows one of the 9 markers included in dominance analyses. **Scatterplots**: Correlation between CT change at the respective predictor’s peak timestep (**y axis**) and the predictor map, corresponding to panel A-bottom. The **first surface** shows the residual difference maps calculated for each marker, highlighting the most influential regions on CT change association effects. For illustration purposes, the **second and third surface** show CT change and the spatial distribution associated with the marker. See Fig. S8 for all residual difference maps, Fig. S4C for all CT change maps, and Ext. Data Fig. 2 for all predictor maps. **Abbreviations**: CT = cortical thickness, see Fig. 2 for abbreviations used in neurobiological marker names.

Structural patterns resulting from the relative distribution of CT across cortical regions vary depending on the time point in life^38^. Developmental changes of these patterns might mirror the contribution of a certain cellular or molecular process to CT changes at a given time point. Using spatial Spearman correlations between each multilevel neurobiological marker and modeled CT at each timepoint, we revealed diverse colocalization trajectories with a general pattern of strongest changes from childhood to young adulthood (up to approximately 30 years) as well as in late adulthood (from 60 years onwards; Ext. Data Fig. 3). Colocalization strengths varied across the CT percentiles extracted from the normative model, but temporal trajectories were consistent. Sex did not relevantly influence the trajectories (Fig. S5).

### 2.3. Neurobiological markers explain CT change

Studying population-level and individual brain development inevitably requires looking at respective changes over time, rather than focusing only on cross-sectional data^39^. We now asked to which extent different neurobiological markers explained relative *change* of representative CT across the lifespan and which markers showed the strongest associations (Figs. S4B and S4C). Multivariate regression analyses predicting CT change across 5-year periods throughout the lifespan (sliding window with 1-year-steps) showed that the combined, either molecular- or cellular-level, markers explained up to 54% of the spatial variance in representative CT changes with peaks during young adulthood (molecular, 20–35 years) and adolescence (cellular, 15–20 years) [false discovery-rate (FDR)-corrected; Fig. 2, top]. Individually, 9 of the 21 multilevel neurobiological markers explained up to 15–38% of representative CT change patterns, with most markers showing peaks up to young adulthood, i.e., between 5 and 30 years of age (FDR-corrected; Fig. 2, bottom). These 9 markers represented major neurotransmitters (dopaminergic, glutamatergic, cholinergic, noradrenergic), features of brain metabolism, neuron populations, and glial cells. Combining all 21 markers across biological levels explained up to 67% of CT changes during the adolescence to adulthood transition (Fig. S6). All findings were robust against correction for baseline CT as well as changes in sliding window step size, modeled sex, and CT percentile (Figs. S6 and S7).

### 2.4. Specific neurobiological markers drive explained CT change

Next, we sought to understand in detail how the 9 significant neurobiological markers contributed to overall explained CT change while accounting for correlation and shared spatial variance patterns between molecular and cellular levels. Given that we found both the strongest *CT changes* and *CT associations* during the period from childhood to young adulthood and given the particular clinical relevance of this timespan, we included CT change from 5 to 30 years as an additional time window for further testing. Using dominance analyses^40,29,30^, we found that the 9 FDR-significant molecular and cellular markers jointly explained 58% of CT change patterns from 5 to 30 years, peaking at the transition from childhood to adolescence (10–15 years; Fig. 3A, top). All 9 neurobiological markers contributed to the overall explained CT change during different life periods (nominal *p* < 0.05) with 6 markers surviving FDR correction (Fig. 3A, middle; Animation S2). During childhood and adolescence, 3 of these 6 markers explained most of the spatial CT change patterns, representing estimates of dopaminergic receptors (*ni9*; *R*^*2*^ = 16%; peek at 8–14 years), microglia and oligodendrocyte progenitor cells (*ce3*; *R*^*2*^ = 12%; 8–15 years), as well as of somatostatin-expressing interneurons (*ce8*; *R*^*2*^ = 12%; 5–14 years). CT change patterns during young and middle adulthood were explained by 2 neurobiological markers broadly associated with the major – i.e., dopaminergic, glutamatergic, cholinergic, and noradrenergic – neurotransmitter systems (*ni3* and *ni5*; 29–56 years). Finally, late adulthood CT aging patterns were associated with a marker representing inhibitory neuron populations and astrocytes (*ce4*, 78–88 years). Except for microglia and oligodendrocyte progenitor cells, all identified associations were negative, i.e., indicating a *stronger reduction* of CT in areas with *higher density* of the respective biological marker.

### 2.5. Specific cortical regions drive CT change associations

The spatial associations reported here are likely dominated by some cortical regions relative to others. By evaluating the impact of iteratively excluding each region from the multivariate models, we found that the most influential regions differed depending on the markers. For example, cellular markers associated to childhood and adolescence CT development (*ce9*: somatostatin-expressing interneurons and *ce3*: microglia) were driven by premotor, cuneus, and frontopolar areas, whereas the association to dopaminergic receptors during this period (*ni9*) was more influenced by primary visual, mid-cingulate, and insular regions. While associations between CT change during young and middle adulthood and cholinergic neurotransmission (*ni5*) exhibited a similar patterns, adult colocalization to dopaminergic neurotransmission (*ni3*) was strongly influenced by sensorimotor areas (Fig. 3B; Text S1.3; Fig. S8; Animation S2).

### 2.6. Factor-level markers reflect original brain atlases

**Fig. 4:**
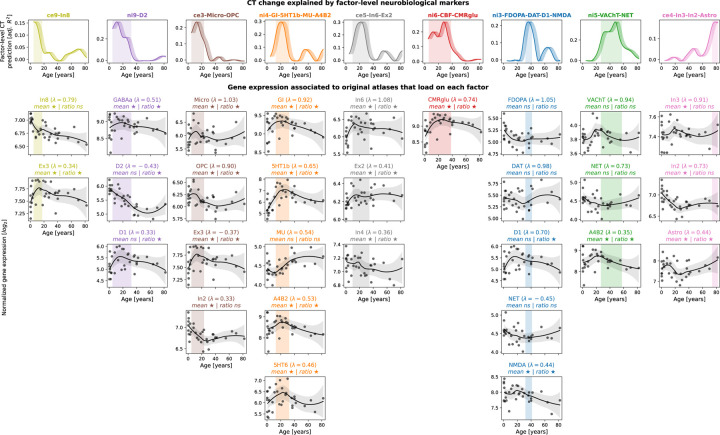
Validation of CT model-based results in developmental gene expression data **First row:** CT change explained by individual neurobiological markers, exactly corresponding to the univariate results in Fig. 2. X values are aligned to the fist year of each tested CT change time period (e.g., “Δ(5,10)” is aligned to 5 years on the x-axis). **Shades following each line** visualize other possible alignments (e.g., “Δ(5,10)” aligned to 6, 7, 8, 9, or 10 years). **Vertical shaded boxes** indicate the time period in which CT change was explained significantly (FDR-corrected). **Following rows:** Normalized log_2_-transformed gene expression trajectories for maximum 5 original atlases that loaded on the factor-level neurobiological marker with λ > |0.3| (cf. Ext. Data Fig. 4). Gene expression for each marker was derived from associated individual genes or from averaging across gene sets. **Grey dots** indicate average neocortical expression of individual subjects. **Black lines and shades** show locally estimated scatterplot smoothing (LOESS) curves with 95% confidence intervals. Associations were tested for by averaging the LOESS data within and outside of each respective time period and comparing mean and ratio against similar data randomly sampled from non-brain genes. ★: FDR-corrected across all tests; ☆: nominal *p* < 0.05. **Abbreviations**: CT = CT change, adj. = adjusted, FDR = false discovery rate, ns = not significant, see Fig. 2 for abbreviations used in neurobiological marker names.

Thus far, we focused on a lower dimensional representation of neurobiological markers, which reduced predictor intercorrelation and increased statistical power, as compared to using the original 49 brain atlases. Nevertheless, we found that original atlases that were most closely related to each factor explained CT change patterns to a similar extent as the factor-level models, aiding interpretation and supporting the validity of the factor-level approach (Ext. Data Fig. 4; Text S1.4; Fig. S9). All univariate spatial associations between CT change and the tested original atlases reached nominal significance (*p* < 0.05). Separate dominance analyses for each factor-level neurobiological marker with only strongly loading original atlases as predictors confirmed contributions of specific original atlases to the factor’s peak explained variance: somatostatin-expressing interneurons, dopaminergic D1 and D2 receptors, as well as glucose metabolism and aerobic glycolysis accounted for most of the associated markers’ peak effects during childhood and adolescence (*ce9*, *ni9*, *ni4*, and *ni6*). Peak effects during young and middle adulthood were mostly accounted for by α4β2 nicotinic receptors and the acetylcholine transporter (*ni5*) as well as the glutamatergic NMDA receptor (*ni3*; Ext. Data Fig. 4).

### 2.7. CT change associations supported by developmental gene expression

Next, we turned to developmental gene expression^9^ to confirm that the biological processes we found associated with cortical development were indeed upregulated in the cortex during the identified developmental period^41^. From a human postmortem dataset (*n* = 33, age range 0.33–82.05 years, see Kang et al.^9^ for details), we estimated gene expression trajectories across the neocortex corresponding to each original brain atlas relevant for the final 9 factor-level neurobiological markers (cf. Ext. Data Fig. 4). For cell-type atlases, we averaged normalized gene expression values across the respective marker genes^33,34^. For molecular markers, we selected genes corresponding to each protein(-compound), in addition to two sets of genes associated with brain metabolism^42^ (Ext. Data Tab. 1). To pose as little assumptions on the sparse data as possible, we compared each gene/gene set during its respective developmental period with a control set of non-brain genes, testing (i) if the gene/gene set showed higher mean expression and (ii) if it showed a “peak” in its trajectory, quantified as a higher ratio of expression during versus outside the developmental period. As expected, most genes/gene sets showed higher mean expression and/or higher expression ratios indicating that they were active in cortical tissue or had their individual peak expression during the respective developmental period (FDR-corrected; Figs. 4 and S10). Notably, for the two molecular markers explaining CT maturation in adulthood (*ni3*: glutamatergic/ dopaminergic and *ni5*: cholinergic/ noradrenergic), we found evidence for associations only with dopaminergic D1 and glutamatergic NMDA receptors for *ni3* as well as with the cholinergic α4β2 receptor for *ni5*.

### 2.8. Multilevel neurobiological markers explain individual CT trajectories

The above analyses successfully demonstrated that specific neurobiological markers account for a large proportion of variance arising from modeled CT change patterns. During the neurodevelopmental period from childhood to young adulthood, 6 markers accounted for about 50% of the total variance with D1/2 dopaminergic receptors, microglia, and somatostatin-expressing interneurons taking the largest share. Relevance of all these 6 markers during their respective associated neurodevelopmental periods could be confirmed in independent gene expression data. However, a sole focus on population CT change, i.e., median predictions from the normative model^3^, does not allow for inferences about individual-level neurodevelopment, which is the mandatory prerequisite for potential clinical applications. A successful validation on the individual level can also provide further evidence for the potential mechanistic relevance of the identified neurobiological markers and supports the use of normative models to non-invasively study neurodevelopmental mechanisms.

**Fig. 5:**
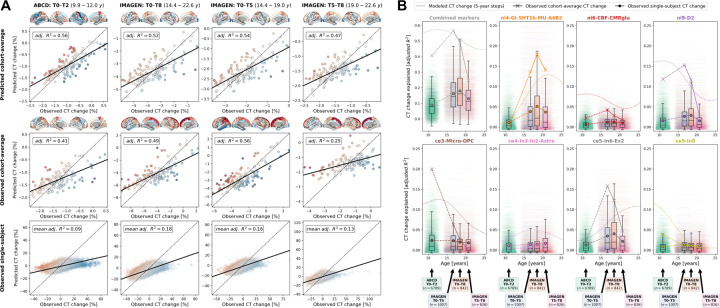
Validation of model-based results in ABCD and IMAGEN datasets (**A**) Explained spatial CT change variance in ABCD and IMAGEN data. The overall model performance is illustrated as scatter plots contrasting predicted CT change (**y axis**) with observed CT change (**x axis**). **Scatters**: single brain regions, **color-coded** by prediction error. **Continuous line**: linear regression fit through the observations. **Dashed line**: theoretical optimal fit. **Brains**: prediction errors corresponding to scatters. **Rows**: upper = cohort-average predicted by the reference (“Braincharts”) model; **middle**: observed cohort-average; **lower**: observed single-subject values, one regression model was calculated for each subject, but the results were combined for illustration purposes. (**B**) Explained spatial CT change variance with a focus on the individual multilevel markers. **Subplots** for the combined analysis and each individual multilevel marker show: modeled CT change as presented in Fig. 3 (**dotted line**); observed cohort-average CT change (**cross markers**); and observed single-subject CT change (**boxplots and dot markers**). For each subject, one **horizontal line** at their individual *R*^*2*^ value ranges from their age at beginning and end of each time span. **Boxplots** show the distribution of individual values for each time span. Note that the first subplot (“Combined markers”) corresponds to the data presented in panel A. See Ext. Data Fig. 5 and Fig. S15 for detailed results. **Abbreviations**: CT = cortical thickness, adj. = adjusted, see Fig. 2 for abbreviations used in neurobiological marker names.

**Fig. 6:**
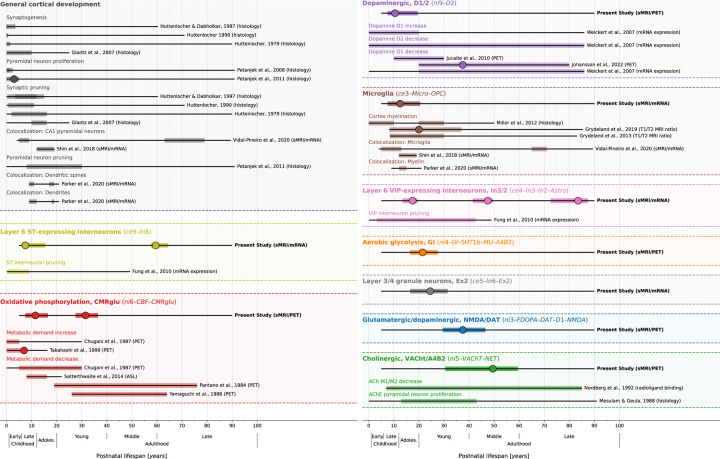
Summary of study findings in the context of prior literature Condensed visualization of the reported results (**first line of each block**, highlighed are markers that showed consistent results) in context with related results of previous human studies investigating similar biological processes or cell populations (**lines below**). Each **header** indicates one neurobiological marker, each thin black line overlaid by a colored bar indicates results from one study. If a study reported multiple results pertaining to the same process (e.g., from two different brain regions), bars were laid over each other. **Thin black lines**: overall time span investigated. **Colored overlay**: time period in which significant associations to CT (change) patterns were reported (nominal *p* < 0.05), independent of the sign of the association. **Large dots**: Timepoint of the maximum association. See also Fig. S20 and Tab. S4 for a more comprehensive overview including various topics. **Abbreviations**: ST = somatostatin, CR = calretinin, sMRI = structural MRI, CBF = cerebral blood flow, PET = positron emission tomography, ASL = arterial spin labeling, ACh(E) = acetylcholine (esterase), see Fig. 2 for abbreviations used in neurobiological marker names.

Using 2-to-8-year longitudinal data from two large cohorts^36,37^, covering the neurodevelopmental period from late childhood to young adulthood (ABCD: *n* = 6,789; IMAGEN: *n* = 985–1117; Demographics and quality control: Text S1.5, Tab. S1, Fig. S11; Observed-vs.-predicted CT change patterns and correlations: Figs. S12 and S13; Effects of site on CT and CT change: Tabs. S2 and S3), we first confirmed that the colocalization between cross-sectional single-subject CT and neurobiological markers mirrored the patterns observed for the modeled population-average (Figs. S5 and S14). In line with these findings, the cohort-average relative change of CT across study timespans (10–12, 14–22, 14–19, and 19–22 years) was explained to extents comparable with the normative model (minimum/maximum observed *R*^*2*^ = 25/56%, model-prediction *R*^*2*^ = 47/56%; Fig. 5A upper and middle). These patterns translated to the single-subject level, explaining on average between 9 and 18% in individual CT changes with considerable variability (range *R*^*2*^ = 0–59%; Fig. 5A, lower; Ext. Data Fig. 5). Looking at individual marker-wise contributions, we again found the model-based patterns to be reflected on both cohort-average and single-subject levels (Fig. 6B; Ext. Data Fig. 5; Fig. S15). While the neurobiological markers predicted to be most important (D1/2 and microglia) indeed explained significant amounts of CT change, two other markers, which primarily reflected aerobic glycolysis (*ni4*) and granule neurons (*ce5*), were equally dominant. Sensitivity analyses showed that CT change predictions (i) generalized from the normative data to individual subjects with above-chance performance but were a poor fit for many individuals, underscoring our focus on individual differences (Text S1.6; Fig S16), (ii) were not relevantly influenced by the reference model-based site-adjustment (Ext. Data Fig. 5; Fig. S15), (iii) increased with longer follow-up duration within each time period (Fig. S17), (iv) varied by sex and study site in some tested time periods (Text S1.7; Fig. S18), and (v) varied with individual atypical CT development as well as data quality (Text S1.8; Fig. S19).

## 3. Discussion

Patterns of spatial colocalization between macroscale brain structure and the underlying neurobiology provide *in vivo* insight into healthy and pathological processes that are otherwise inaccessible to human studies. We find that the colocalization between developmental changes of cortex morphology and corresponding adult-derived neurotransmitter receptor, brain metabolism, and cell type profiles closely reflects neurodevelopmental processes across various biological levels (see Figs. 6, S20, and Tab. S4 for a descriptive overview). While synaptogenesis and neuronal and glial proliferation continue into the first postnatal years, the second and third life decades are marked by a targeted reduction of neurons and cell components, likely reflecting functional specialization^9,43–50^. In line with our findings, dopamine D1 receptor activity was reported to peak in adolescence and young adulthood before declining steadily with age^51–53^. Our results concerning somatostatin-expressing interneurons fit with prior reports showing a marked decrease of somatostatin interneuron markers within the first decade of life^50^. Similarly in line with our findings, microglia have been implicated in synaptic remodeling^6,12,16^ and in myelination^54,55^, which has been shown to continue into young adulthood^11,15,56–58^. Approaching adulthood, cortical development becomes less dynamic with most regions taking stable or steadily decreasing aging trajectories^1,3^. We find these phases to be reflected in spatial colocalization patterns in that most neurobiological markers colocalize with CT changes in early cortex development. Only the cholinergic system consistently predicts cortical changes throughout young and late adulthood, potentially pointing to its role in healthy and pathological aging^59^.

Normative modeling of large-scale neuroimaging data has received considerable attention as a tool to translate basic research into clinical applications^1,3,27,60^. We show that if used as a reference for typical brain development, combining normative models of brain regional features with spatial colocalization approaches can facilitate discovery of physiological mechanisms underlying specific conditions. Going beyond this group-level discovery approach, we demonstrate the feasibility of spatial colocalization analyses in single subjects by mapping individual-level deviations from normative trajectories to specific neurobiological markers. In view of this ability of molecular and cellular neurobiological markers to explain *typical* developmental and maturational patterns of the cortex, studying how these findings translate to *atypical* neurodevelopment^61^ is a promising path for future research. Establishing a robust mapping between deviating brain developmental patterns and underlying biological processes provides value not only for biomarker discovery but also for identification of potential therapeutic targets. As promising candidates for clinical translation, we identify the dopaminergic system^23,62^ and microglial cell populations^63^ for early development, as well as the cholinergic system in context of pathological aging^64^.

Importantly, spatial association analyses as applied here do not impose causality and, thus, the reported associations only provide indirect evidence for involvement of specific neurobiological markers in cortical maturation and therewith potential guidance to future causal studies of specific processes. The analyses are also limited by the heterogeneity of the brain atlases which were derived from independent adult populations of varying age and sex, processed with different strategies, and in part – as was the developmental gene expression data – derived from *postmortem* samples^19^. Similar restrictions apply to the normative CT model which is largely based on the WEIRD (Western, Educated, Industrialized, Rich, and Democratic^65^) population^3,66^. The contribution of these factors needs to be quantified in future research. Nonetheless, the high replicability of the observed associations despite the noise introduced by these limitations rather strengthens the robustness of our findings.

## 4. Methods

### 4.1. Ethics

No new human data were acquired for this study. Ethical approval for usage of publicly available and restricted-access databanks including human demographic, behavioral, and neuroimaging data has been granted by the Heinrich-Heine-University, Düsseldorf, Germany. Specific approval for collection and sharing of the used data (brain atlases, Braincharts model, Human Brain Transcriptome, ABCD, and IMAGEN) were provided by local ethics committees; detailed information is available in the cited sources. Use of the ABCD data is registered at the NDA database at http://dx.doi.org/10.15154/1528657. The responsible IMAGEN investigator is T. Banaschewski.

### 4.2. Software

Multimodal brain atlases were retrieved and processed from/with *neuromaps*^35^, *abagen*^67^, *JuSpace*^28^, or author sources. Analyses of associations between CT and cortical atlases were conducted using *JuSpyce 0.0.2*^68^ in a *Python 3.9.11* environment^68^. *JuSpyce* (https://github.com/LeonDLotter/JuSpyce) is a toolbox allowing for flexible assessment and significance testing of associations between multimodal neuroimaging data, relying on imaging space transformations from *neuromaps*^35^, brain surrogate map generation from *brainSMASH*^69^, and several routines from *Nilearn*^70^, *scipy*^71^, *NiMARE*^72^, *statsmodels*, *numpy*, and *pandas*. Visualizations were created using *matplotlib*^73^, *seaborn*^74^, and *surfplot*^75^. The *PCNtoolkit*^76,77^ was used to generate modeled CT data, as well as site-adjusted/predicted CT data and deviation scores for ABCD and IMAGEN subjects.

### 4.3. Data sources and processing

#### 4.3.1. Atlases of molecular and cellular neurobiological markers

Multilevel neurobiological atlases (Ext. Data Fig. 1) were separated into two broad categories according to their source modality. Sample characteristics and data sources are provided in Ext. Data Tab. 1.

The *neuroimaging* (“*ni-*”) dataset consisted of group-average nuclear imaging atlases (neurotransmitter receptors, brain metabolism and immunity, synaptic density, and transcriptomic activity) and an MRI-based marker of cortical microstructure (T1/T2 ratio; Text S1.1.1)^28,29,35,78–81^. Maps were (i) transformed from fsLR (metabolism and T1/T2) or Montreal Neurological Institute space (all others) to fsaverage5 space using registration fusion^35,82^, (ii) parcellated in 74 cortical regions per hemisphere^83^, and (iii) *Z*-standardized across parcels within each atlas.

*Cell type* (“*ce-*”) atlases were built by (i) retrieving genetic cell type markers identified by Lake et al.^33^ and Darmanis et al.^34^ via single-nucleus RNA sequencing in human brain tissue from the PsychENCODE dataset^84^, (ii) extracting Allen Human Brain Atlas mRNA expression values^18^ for each Destrieux parcel and each marker gene using *abagen*^67^ (default settings, data mirrored across hemispheres, Text S1.1.2), (iii) *Z*-standardizing the data across parcels within each gene, and (iv) taking the uniform average of the data across genes within each cell type.

We reduced the dimensionality of the atlas datasets to decrease multicollinearity in multivariate regression analyses. As the nuclear imaging and mRNA expression data likely differed strongly in terms of confounds and signal-to-noise ratio, and to study molecular- and cellular-level effects separately, data sources were not mixed during dimensionality reduction. To retain interpretability, we used factor analysis for dimensionality reduction (minimum residuals, promax rotation). All unrotated factors that explained ≥ 1% of variance of each dataset were retained. We chose the oblique rotation method as the resulting factor intercorrelation would be expected from non-independent biological processes or cell populations. Resulting predictors were named by assigning each original atlas to the factor it loaded on the most (nuclear imaging: *ni1–n*; mRNA expression: *ce1–n*; MRI: only microstructural marker, no dimensionality reduction: *mr1*). In an additional analysis, we ensured that the factor solution estimated on the original brain atlases explained more variance in the original dataset than factor analyses estimated on permuted brain maps (see Text 1.1.5).

#### 4.3.2. Braincharts CT model

The *Braincharts* reference model was estimated on 58,836 subjects from 82 sites (50% training/testing split; 51% female; age range 2.1–100 years; age distribution: Fig. S3). Detailed information on the included samples, CT estimation, and modeling procedure was provided by Rutherford et al.^3^. Notably, while ABCD baseline data were included in the model estimation, ABCD follow-up and IMAGEN data were not. Briefly, T1-weighted MRI data were obtained from the original cohorts and FreeSurfer 6.0^85^ was used to extract parcel-wise CT data. Image quality was ensured based on FreeSurfer’s Euler characteristic^86^ and manual quality control of 24,354 images^3,36^. CT development was modeled separately for each Destrieux parcel using warped Bayesian linear regression models predicting CT from age, sex, and site as fixed effect. The applied methodology was developed for use in large datasets, can model nonlinear and non-Gaussian effects, accurately accounts for confounds in multisite datasets, and allows for estimation of site batch effects in previously unseen data^3,87–89^.

We extracted Braincharts CT data separately for females and males for each of 148 cortical parcels for 171 timepoints (5–90 years with 0.5-year steps) and 7 percentiles (1^st^, 5^th^, 25^th^, 50^th^, 75^th^, 95^th^, and 99^th^). We focused on CT data from the age of 5 years onwards as the used FreeSurfer pipeline was not adjusted for very young ages^3^. For colocalization analyses, the extracted modeled CT data were used as is. For model-based (pseudo-)longitudinal analyses, we calculated the relative CT change ΔCT from year i to year j based on the median (50^th^ percentile) sex-average CT data as $\text{Δ}CT_{\left(i,j\right)}=\frac{CT_{j}-CT_{i}}{CT_{i}}$. Lifespan CT change was then calculated using a sliding window with 1-year steps and 5-year length from 5 to 90 years as $\text{Δ}CT_{\left(i,j\right)}$, $i\in \left[5..85\right]$, j=i+5.

#### 4.3.3. ABCD and IMAGEN CT data

Processed and parcellated CT data from the Adolescent Brain Cognitive Development (ABCD) cohort^36^ was taken directly from the ABCD 4.0 release. Baseline (T0, ~10 years) and 2-year follow-up (T2) structural MRI data were processed using FreeSurfer 7.1.1. Details were provided by Casey et al.^36^ and in the release manual (http://dx.doi.org/10.15154/1523041). For the IMAGEN cohort^37^, T1-weighted MRI data at baseline (T0, ~14 years) and at one or two follow-up scans (T5, ~19, and T8, ~22 years) were retrieved and processed with FreeSurfer’s standard pipeline (7.1.1). Following Rutherford et al.^3^, for quality control we relied on an Euler-like metric, i.e., the total number of surface defects as provided by FreeSurfer. We excluded subjects that exceeded a threshold of Q3+IQR×1.5 calculated in each sample across timepoints^86,90^ or failed the manual quality ratings provided in the ABCD dataset. One ABCD study site (MSSM) stopped data collection during baseline assessment and was excluded. We utilized the Braincharts model to harmonize CT data of the two datasets across sites (ABCD: n = 20; IMAGEN: n = 8) and to derive individual deviation scores to be used only in sensitivity analyses. Site effects were estimated in healthy subsamples of both dataset’s baseline data (n = 20 per site, 50% female) distributed evenly across baseline age ranges. These subjects including their follow-up data, and all subjects with data for less than two study time points, were excluded from further analyses. As the ABCD baseline data were used in training the Braincharts model, we conducted sensitivity analyses on the non-adjusted data to estimate potential overfitting effects.

Colocalization analyses were calculated on the site-adjusted and original CT values at each timepoint. For longitudinal analyses, the relative CT change between each time point within each cohort was calculated as above (ABCD: T0–T2; IMAGEN: T0–T8, T0–T5, and T5–T8).

### 4.4. Null map-based significance testing

Spatial associations between brain maps can be assessed in correlative analyses in the sense of testing for cortex- or brain-wide alignment of the distributions of two variables A (e.g., CT) and B (e.g., a neurotransmitter receptor)^11,28,29,91^. Effect sizes (e.g., correlation coefficients) resulting from correlating A and B convey interpretable meaning. However, parametric *p* values do not, as they are influenced by the rather arbitrary number of “observations” (between thousands of voxels/vertices and a few parcels) and spatial autocorrelation in the brain data^92^. Null model-based inference approaches circumvent this problem by comparing the observed effect size to a null distribution of effect sizes obtained by correlating the original brain map A with a set of permuted brain maps generated from B to derive empirical *p* values. From several approaches proposed to preserve or reintroduce spatial autocorrelation patterns in null maps^92^, we relied on the variogram-based method by Burt et al.^69^ as implemented in *JuSpyce* via *BrainSMASH*^35,68,69^.

### 4.5. Discovery analyses based on the Braincharts model

#### 4.5.1. Lifespan colocalization trajectories

To characterize lifespan trajectories of colocalization between cross-sectional CT and multilevel neurobiological markers, we calculated Spearman correlations between each brain atlas and modeled CT data at each extracted time point and percentile. Smoothed regression lines (locally estimated scatterplot smoothing) were estimated on data from all percentiles combined to highlight developmental trajectories. As the resulting developmental patterns were largely similar across sexes, we performed the main analyses on female-male averaged CT data and reported sex-wise results in the supplementary materials.

#### 4.5.2. Prediction of CT change

The main objective of this study was to determine the extent to, and the temporal patterns in which, multilevel neurobiological marker could explain CT development and lifespan change. To achieve this goal, we designed a framework in which we “predicted” stepwise relative CT change from one or more brain atlases in multivariate or univariate regression analyses. The amount of CT variance explained *R*^*2*^ was used as the main outcome measure (adjusted in multivariate analyses). Exact one-sided *p* values were calculated by generating a constant set of 10,000 null maps for each brain atlas and comparing observed *R*^*2*^ values to *R*^*2*^ null distributions obtained from 10,000 regression analyses using the null maps as predictors.

To determine the general extent to which CT development could be explained, we performed one multilinear regression per lifespan timestep (81 models) using (i) all neuroimaging and (ii) all mRNA expression-based atlases. In an additional analysis, we assessed the result combining all atlases irrespective of modality. The resulting *p* values were FDR-corrected across all models and atlas source modalities. To quantify individual atlas-wise effects and identify specific neurobiological markers of potential relevance to CT development, we performed univariate regression analyses per timestep and atlas (21 × 81 models), correcting for multiple comparisons using FDR correction within each modality. In sensitivity analyses, we assessed effects of correcting for baseline CT (regression of cross-sectional CT at year x from CT change between year x and year y), adjusting CT percentile (1^st^ and 99^th^), sex (female and male separately), and window length (1-year, 2-year). As above, the results were consistent across sexes, thus all main analyses were reported for sex-average CT data and the following model-based analyses were performed only on sex-average data.

#### 4.5.3. Marker-wise contributions to explained CT change

Aiming to identify when and how neurobiological markers contributed to explaining CT change, we retained only those brain atlases that significantly explained CT development individually (FDR correction) and conducted dominance analyses “predicting” CT change from this joint set of atlases. Dominance analysis aims to quantify the relative importance of each predictor in a multivariate regression. The *total dominance* statistic is calculated as the average contribution of a predictor *x* to the total *R*^*2*^ across all possible subset models of a multivariate regression and can here be interpreted as the extent to which CT development during a certain time period is explained by *x* in presence of the whole set of predictors *X* and as a fraction of the extent to which CT development is explained by set *X*^29,30,40^. Following from this, in our models, the sum of the atlas-level *R*^*2*^ at a given timespan equals the total *R*^*2*^ at this time point. Significance of dominance analyses was determined as described above by generating null distributions and estimating empirical *p* values for both, the “full model” multivariate *R*^*2*^ and the predictor-wise total dominance *R*^*2*^. Finally, Spearman correlations between CT change and multilevel brain atlases were conducted to indicate the directionality of associations.

Dominance analyses were conducted at each timestep and, to highlight the main postnatal developmental period between child and adulthood, on the CT development across this entire period defined as $\text{Δ}CT_{\left(5,30\right)}$ (82 models). Resulting *p* values were corrected across the whole analysis (full model and atlas-wise: 82 + 82 × 9 *p* values).

#### 4.5.4. Brain-regional influences on CT change association patterns

To estimate the importance of individual brain regions for the associations between CT change and brain atlases, we relied on the atlas-wise *residual differences* across brain-regions as unitless measures of the influence of individual cortex regions on the dominance analysis results. The residual difference of prediction errors ΔPE for each predictor x out of the predictor set X was calculated as $\text{Δ}PE=\left|PE_{X\backslash \left\{x\right\}}\right|-\left|PE_{X}\right|$. The results were visualized on surface maps for descriptive interpretation.

#### 4.5.5. Relationships between dimensionality-reduced and original multilevel atlases

Assessing whether the factor-level atlases represented the original multilevel atlases according to the applied atlas-factor-association scheme, we performed (i) dominance analyses and (ii) univariate regressions per *factor-level atlas* using only the strongest associated *original atlases* as predictors. The latter were defined as the five atlases that loaded the most on each factor if the absolute loading exceeded 0.3. FDR correction was performed independently for dominance analyses and univariate regressions across all tests.

### 4.6. Validation analyses based on developmental gene expression data

#### 4.6.1. Data sources and (null) gene set construction

Normalized developmental gene expression data for *n* = 17,565 genes was downloaded from the Human Brain Transcriptome database (https://hbatlas.org/pages/data); the original dataset was published by Kang et al.^9^. As of the postnatal focus of our study, we included only subjects after birth, resulting in *n* = 33, aged between 0.33 and 82.05 years. The original data was sampled across multiple cortical regions and, in some cases, both hemispheres per subject. However, because a maximum of only 11 cortex regions was sampled, we decided to average the data per subject across hemispheres and neocortical areas (cf. Kang et al.).

We identified the original brain atlases that loaded most strongly on each factor-level multilevel neurobiological marker (cf. section 4.6.5). Each of these original atlases was represented in the genetic data through a single gene or a set of genes (Ext. Data Tab. 1); in case of gene sets, gene expression data was averaged across genes. For most nuclear imaging maps, we selected the genes or gene sets that coded for, or was associated with, the respective tracer target. For brain metabolism maps, we took two gene sets associated with aerobic and anaerobic glycolysis from Goyal et al.^42^. We did not have a gene set for the CBF (cerebral blood flow) map. For cell type maps, we took the original gene sets from which the maps were generated^33,34^.

For permutation-based significance testing (see below), we created *n* = 10,000 null gene expression datasets by randomly selecting genes or same-sized gene sets from *n* = 2,154 non-brain genes (https://www.proteinatlas.org/humanproteome/brain/human+brain, “Not detected in brain”).

#### 4.6.2. Associations to temporal patterns of explained CT development

The following process was used to test for associations between CT change explained by neurobiological markers and developmental gene expression trajectories: (i) We fitted a smoothed regression line (locally estimated scatterplot smoothing) to the gene expression data associated with each gene/ gene set as well as to the respective null gene expression datasets. (ii) For each neurobiological marker from section 4.6.3, we identified the time period in which it explained CT change significantly (FDR-corrected). (iii) For each of the (null) gene expression trajectories associated with the current neurobiological marker, we calculated the average gene expressions during and outside of the significant time period. (iv) We separately compared the mean and the ratio of gene expression during vs. outside the significant time period between the observed and null gene expression data to derive empirical one-sided *p* values for the association between each multilevel neurobiological marker and each associated gene/gene set. (v) FDR-correction was applied across all tests at once. A significantly higher mean expression would indicate increased cortical expression of a marker during the tested time period as compared to non-brain genes. An increased ratio would broadly indicate that the marker’s gene expressions peaks during the tested time period.

### 4.7. Validation analyses based on ABCD and IMAGEN single-subject data

#### 4.7.1. Developmental colocalization trajectories

First, we tested whether colocalization patterns between multilevel atlases and single-subject cross-sectional CT followed the predictions of the Braincharts model. Spearman correlations were calculated between each subject’s CT values and each atlas at all available timepoints, for both site-adjusted CT data and the data prior to site-effect-correction.

#### 4.7.2. Explained CT development patterns on cohort- and single-subject levels

Following, we assessed how neurobiological markers that significantly explained modeled CT development during the period covered by ABCD and IMAGEN data (9–25 years) performed in single-subject longitudinal data. Dominance analyses were performed in two steps. First, for each of the four investigated time spans (ABCD: ~10–12; IMAGEN: ~14–22, ~14–19, 19–22 years), one dominance analysis was calculated to predict the *cohort-average* CT change pattern from neurobiological markers. Second, dominance analyses were calculated in the same fashion, but *for every subject*. For comparison, analyses were repeated on CT change patterns as predicted by the Braincharts model from each subject’s age and sex. For cohort-average dominance analyses, exact *p* values were estimated as described for the stepwise model-based analyses. For individual-level analyses, instead of estimating p values for each subject, we tested whether the mean *R*^*2*^ values of the full models and each predictor observed in each cohort and time span were significantly higher than was estimated in 1,000 null-analyses with permuted atlas data. Finally, we repeated subject-level analyses on the original CT change data prior to site-effect-correction and on the longitudinal change of deviation Z scores as returned by the Braincharts model^3^.

Finally, we evaluated how the subject-level regression models predicting CT change patterns from neurobiological markers generalized from the subject-level normative CT change patterns to the actual observed CT change. For that, we applied the regression model parameters estimated on each subject’s normative CT change patterns to each subject’s observed CT change and evaluated model fit as the subject-level correlation between predicted and observed CT change. To estimate the effect size, results were contrasted to null analyses in which each regression model was estimated using 1,000 permuted multilevel brain maps. Further sensitivity analyses were conducted to estimate how CT change predictions were affected by follow-up duration, sex, study site, “normativity” of CT and CT change patterns [correlation between predicted and observed CT (change), average Braincharts CT deviation (change), count of extreme deviation (change)], and data quality (number of surface defects). Subject-level full model *R*^*2*^ values were compared by sex and study site using analyses of covariances corrected for follow-up duration (and sex). All other variables were correlated with full model *R*^*2*^ values using Spearman correlations.

## Extended Data

**Ext. Data Fig. 1:**
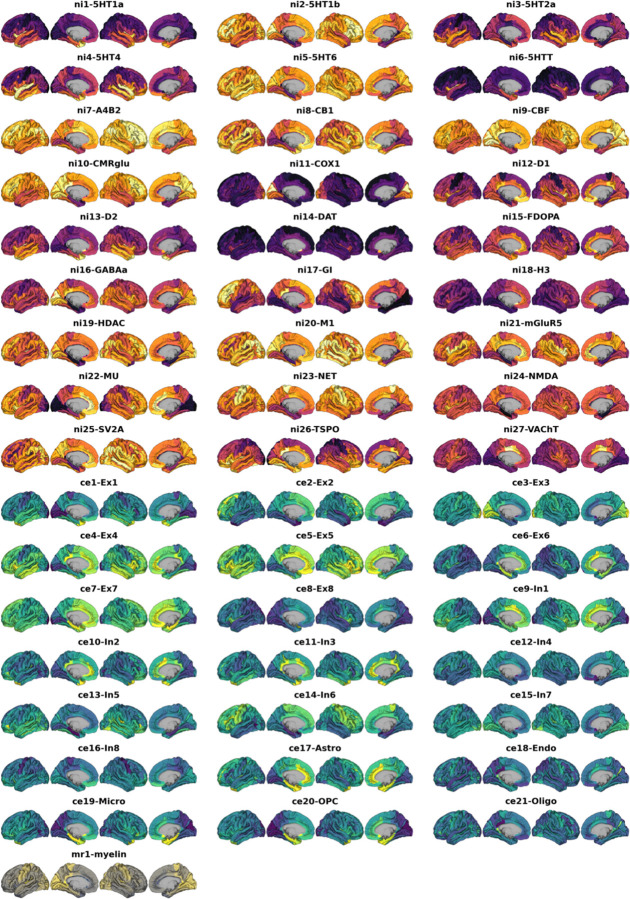
Multimodal nuclear imaging and neural cell type atlases Multimodal atlases after transformation to FreeSurfer space and parcellation into 148 cortical parcels (Destrieux parcellation). Nuclear imaging maps are colored **orange-violet**, gene expression maps **yellow-green**, and the microstructural map is colored **yellow-gray**. See Ext. Data Tab. 1 for individual descriptions and sources.

**Ext. Data Fig. 2:**
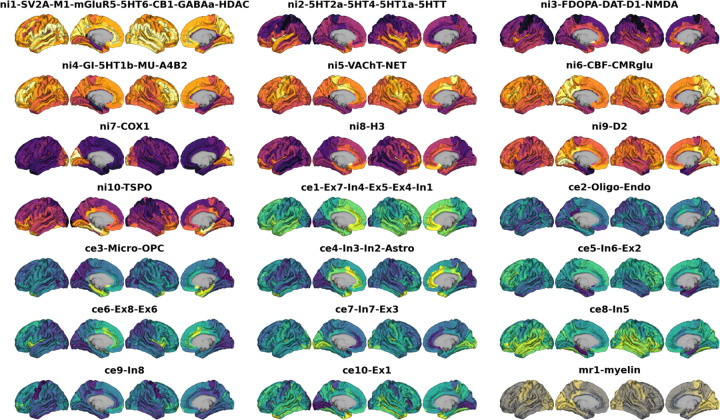
Multilevel neurobiological markers after factor analysis Parcellated brain atlases after dimensionality reduction mapped to the cortex. Nuclear imaging factors are colored **orange-violet**, gene expression factors **yellow-green**, and the microstructural map is colored **yellow-gray**.

**Ext. Data Fig. 3:**
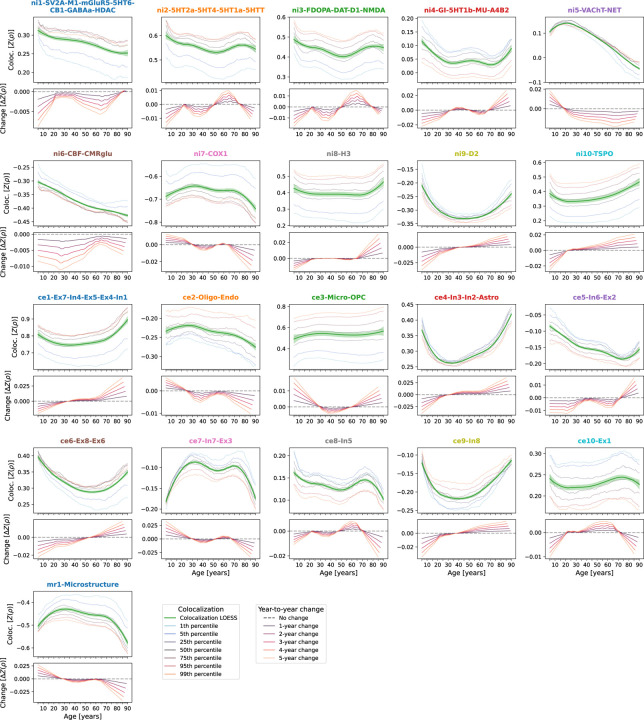
alization between cross-sectional representative CT and multilevel neurobiologial markers across the lifespan **Coloc** Lifespan trajectories of colocalization between multilevel neurobiological brain markers and cross-sectional CT. For each marker, the **upper panel** shows the colocalization trajectory: Z-transformed Spearman correlation coefficients are shown on the **y axis**, age on the **x axis**; **blue-to-orange lines** indicate percentiles of modeled CT data (see legend, note that these do not show actual percentiles of colocalization strengths); the **green line** (LOESS = locally estimated scatterplot smoothing) was smoothed through the percentile data to highlight trajectories. The **lower panel** shows year-to-year changes **(y axis)** derived from the LOESS line in the upper plot. See Fig. S5A for results including ABCD and IMAGEN subjects.

**Ext. Data Fig. 4:**
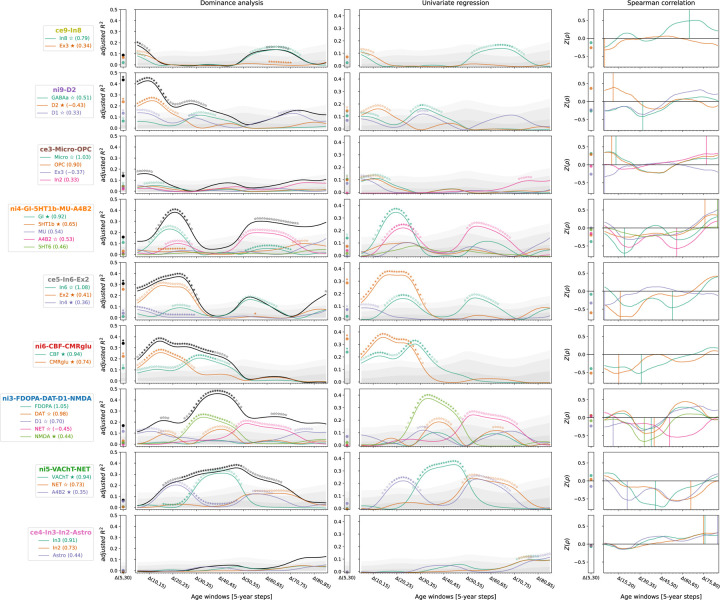
Individual dominance analyses using original multilevel atlases To determine if the factor-level predictors appropriately captured the original multimodal atlases, sets of spatial association analyses were calculated, predicting CT change across the lifespan from the original maps most closely associated to each factor. For each factor, the 5 original atlases that loaded most strongly on the factor were selected if their loading exceeded a threshold of 0.3. The **first column** shows the result of stepwise dominance analyses (**black line** = combined *R*^*2*^), the **second column** shows independent single linear regressions, and the **third column** depicts the colocalization pattern between CT change and original predictors to illustrate the sign of the spatial association. **Gray shades** in the first two columns show the null distributions associated with the individual factor-level *R*^*2*^ values. **Abbreviations**: See Figs. 2 and 3.

**Ext. Data Fig. 5:**
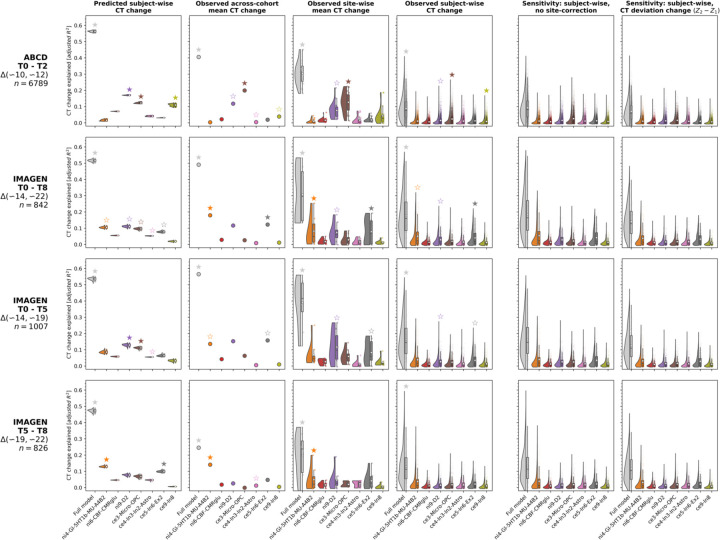
Detailed results of ABCD and IMAGEN validation analyses Explained CT change in ABCD and IMAGEN cohorts across 4 study time periods (**rows**) in different analysis settings. **Raincloud** plots and **scatters** show distributions of *R*^*2*^ values resulting from cohort- or subject-wise dominance analyses. The **leftmost gray elements** depict the full model explained variance, the **right-sided colored elements** show the predictor-wise estimated *total dominance* statistics. For each subject- or cohort-wise analysis, the sum of predictor-wise *R*^*2*^ values equals the full model value. **Stars** indicate statistical significance determined based on null distributions of R^2^ values as estimated by rerunning regression analyses with predictor null maps (FDR-correction within each analysis/panel). See Fig. S15 for equivalent plots showing Spearman correlations. **(Column 1)** prediction result based on CT change as predicted by the Braincharts model. **(Column 2)** average CT change across each cohort. **(Column 3)** average CT change across each site within each cohort. **(Column 4)** CT change in single subjects. **(Column 5 and 6)** sensitivity analyses on subject-level data; **(5)** values without site-correction to estimate effects of overfitting, and **(6)** change between deviation Z scores as estimated by the normative model.

**Ext. Data Tab. 1:** Characteristics and data sources of the multilevel brain atlases **Upper**: Nuclear imaging maps from different primary data sources. The column *Tracer/ Incl. markers* shows the used tracers. **Middle**: Microstructural map, derived from T1/T2 ratio. **Lower**: Neural cell-types constructed from Allen Brain Atlas gene expression data. Here, the column *Tracer/ Incl. markers* shows “gene markers available in Allen Brain Atlas / gene markers available”. The column *N* refers to the number of subjects used to obtain the data. The columns *Genes/ Gene sets* list the genes used for developmental gene expression analyses as well as the numbers of genes that were available in the processed gene expression dataset. All references are cited in the main text or supplementary materials. **Abbreviations**: SD = standard deviation, MRI = magnetic resonance imaging, Incl. = included.

Target system	Name	Target/Information	Tracer/ Incl. markers	N	Male [%]	Age [years]				Source	Genes/ Gene sets	
						mean	SD	min	max		Genes	Incl. markers
**Nuclear imaging**												
**Serotonin**	**5HT1a**	5-HT1a receptor	[11C]CUMI-101	8	37.50	28.40	8.80	n.a.	n.a.	Beliveau et al., 2017	HTR1A	1 / 1
	**5HT1b**	5-HT1b receptor	[11C]P943	65	n.a.	33.70	9.70	n.a.	n.a.	Gallezot et al., 2010	HTR1B	1 / 1
	**5HT2a**	5-HT2a receptor	[11C]Cimbi-36	29	51.72	22.60	2.70	n.a.	n.a.	Beliveau et al., 2017	HTR2A	1 / 1
	**5HT4**	5-HT4 receptor	[11C]SB207145	59	69.49	25.90	5.30	n.a.	n.a.	Beliveau et al., 2017	HTR4	1 / 1
	**5HT6**	5-HT6 receptor	[11C]GSK215083	30	n.a.	36.60	9.00	n.a.	n.a.	Radhakrishnan et al., 2018	HTR6	1 / 1
	**5HTT**	Serotonin transporter	[11C]DASB	100	29.00	25.10	5.80	n.a.	n.a.	Beliveau et al., 2017	SLC6A4	1 / 1

**Dopamine**	**FDOPA**	Dopamine synthesis	[18F]fluorodopa	12	n.a.	n.a.	n.a.	n.a.	n.a.	Garcia-Gomez et al., 2018	DDC	1 / 1
	**D1**	D1 receptor	[11C]SCH23390	13	46.00	33.00	13.00	n.a.	n.a.	Kaller et al., 2017	DRD1	1 / 1
	**D2**	D2 receptor	[11C]FLB457	55	n.a.	32.50	9.70	n.a.	n.a.	Sandiego et al., 2015	DRD2	1 / 1
	**DAT**	Dopamine transporter	[123I]-FP-CIT	174	n.a.	61.00	11.00	n.a.	n.a.	Dukart et al., 2018	SLC6A3	1 / 1

**Acetylcholine**	**A4B2**	α4β2 nicotinic receptor	[F18]flubatine	30	n.a.	33.50	10.70	n.a.	n.a.	Hillmer et al., 2016	CHRNA4, CHRNB2	2 / 2
	**M1**	Muscarinic receptor 1	[11C]LSN3172176	24	n.a.	40.50	11.70	n.a.	n.a.	Naganawa et al., 2021	CHRM1	1 / 1
	**VAChT**	Vesicular acetylcholine transporter	[18F]FEOBV	18	n.a.	66.80	6.80	n.a.	n.a.	Aghourian et al., 2017	SLC18A3	1 / 1

**Glutamate**	**mGluR5**	Metabotropic receptor 5	[11C]ABP688	73	n.a.	19.90	3.04	n.a.	n.a.	Smart et al., 2019	GRM5	1 / 1
	**NMDA**	NMDA receptor	[18F]GE-179	29	72.00	41.00	13.00	n.a.	n.a.	Galovic et al., 2021	GRIN1, GRIN2A–D	5 / 5

**GABA**	**GABAa**	GABA-A receptor	[11C]flumazenil	10	100.00	26.60	7.00	n.a.	n.a.	Kaulen et al., 2022	GABRA1–6, B1–3, G1–3, D, E, P,Q	16 / 16

**Noradrenaline**	**NET**	Noradrenaline transporter	S,S-[11C]O-MRB	77	n.a.	33.40	9.20	n.a.	n.a.	Ding et al., 2010	SLC6A2	1 / 1

**Endorphins**	**MU**	Mu opioid receptor	[11C]carfentanil	204	64.71	32.30	10.80	20.00	59.00	Kantonen et al., 2020	OPRM1	1 / 1

**Cannabinoid**	**CB1**	Cannabinoid receptor 1	[11C]OMAR	77	n.a.	30.00	8.90	n.a.	n.a.	Normandin et al., 2015	CNR1	1 / 1

**Histamine**	**H3**	Histamine receptor 3	[11C]GSK189254	8	n.a.	31.70	9.00	n.a.	n.a.	Gallezot et al., 2017	HRH3	1 / 1

**Synaptic density**	**SV2A**	Synaptic vesicle glycoprotein 2A	[11C]UCB-J	10	70.00	36.00	10.00	n.a.	n.a.	Finnema et al., 2016	SV2A	1 / 1

**Gene transcription**	**HDAC**	Histone deacetylase	[11C]Martinostat	8	50.00	28.60	7.60	18.00	44.00	Wey et al., 2016	HDAC1–11	11 / 11

**Immune activity**	**COX1**	Cyclooxygenase 1	[11C]PS13	11	63.64	30.82	6.69	23.00	42.00	Kim et al., 2020	PTGS1	1 / 1
	**TSPO**	Translocator protein	[11C]PBR28	6	33.33	57.80	8.10	n.a.	n.a.	Lois et al., 2018	TSPO	1 / 1

**Metabolism**	**CBF**	Cerebral blood flow	[15O]H2O								*n.a.*	*n.a.*
	**CMRglu**	Cerebral metabolic rate of glucose	[18F]FDG	33	42.42	25.40	2.60	20.00	33.00	Vaishnavi et al., 2010	Goyal et al., 2014	36 / 38
	**GI**	Glycolytic index	[15O]H2O / [15O]O2								Goyal et al., 2014	113 / 116
**MR imaging**												
**Cortical microstructure**	**Microstr**	T1/T2 MRI ratio	T1wT2w	417	46.28	n.a.	n.a.	22.00	37.00	Human Connectome Project		*n.a.*

**mRNA expression**												
**Excitatory neurons**	**Ex1**	Cortical projection neuron; L2/3	17 / 18									16 / 18
	**Ex2**	Granule neuron; L3/4	9 / 11									11 / 11
	**Ex3**	Granule neuron; L4	9 / 9									8 / 9
	**Ex4**	Subcortical projection neuron; L4	15 / 17									16 / 17
	**Ex5**	Subcortical projection neuron; L4–6	21 / 21									12 / 21
	**Ex6**	Subcortical projection neuron; L5/6	34 /38									36 /38
	**Ex7**	Corticothalamic projection neuron	6 / 6									4 / 6
	**Ex8**	Corticothalamic projection neuron; L6	54 / 64									54 / 64
	
**Inhibitory neurons**	**In1**	VIP+, RELN+, NDNF+; L1/2	2 / 2									2 / 2
	**In2**	VIP+, RELN-, NDNF-; L6	7 / 7	<= 6	83.33	42.50	13.38	24.00	57.00	Lake et al., 2016; Darmanis et al., 2015; Allen Brain Atlas	Original gene sets from Lake et al., 2016 and Darmanis et al., 2015	6 / 7
	**In3**	VIP+, RELN+, NDNF-; L6	17 / 19									17 / 19
	**In4**	VIP-, RELN+, NDNF+; L1–3	11 / 11									10 / 11
	**In5**	CCK+, NOS1+, CALB2+; L2/3	7 / 11									10 / 11
	**In6**	PVALB+, CRHBP; L4/5	5 / 6									5 / 6
	**In7**	SST+, CALB1+, NPY+; L5/6	10 / 13									11 / 13
	**In8**	SST+, NOS1+; L6	3 / 4									4 / 4
	
**Glia cells**	**Astro**	Astrocytes	37 / 40									37 / 40
	**Endo**	Endothelial cells	76 / 82									78 / 82
	**Micro**	Microglia	21 / 25									24 / 25
	**OPC**	Oligodendrocyte progenitor cells	40 / 53									50 / 53
	**Oligo**	Oligodendrocytes	35 / 35									32 / 35

## Supplementary Material

Supplement 1

Supplement 2

Supplement 3

Supplement 4
