# Supplementary figures and images for: Regional patterns of human cortex development correlate with underlying neurobiology
Source: bioRxiv. 2024 Aug 10:2023.05.05.539537. Originally published 2023 May 5. Preprint. [Version 4] doi: 10.1101/2023.05.05.539537 (PMC10187287; doi:10.1101/2023.05.05.539537)

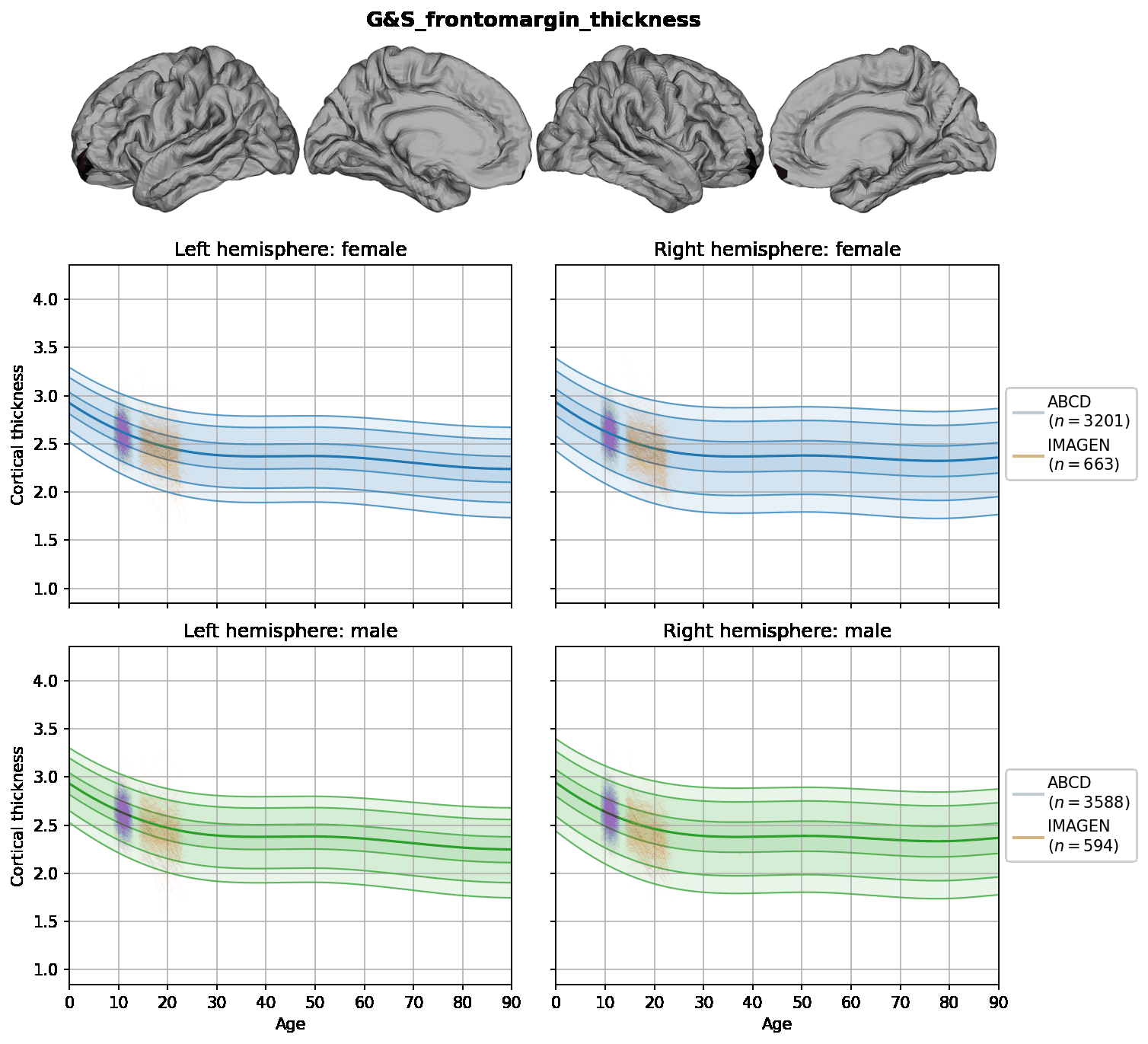

Supplement: Supplement 2 [file media-2.gif]

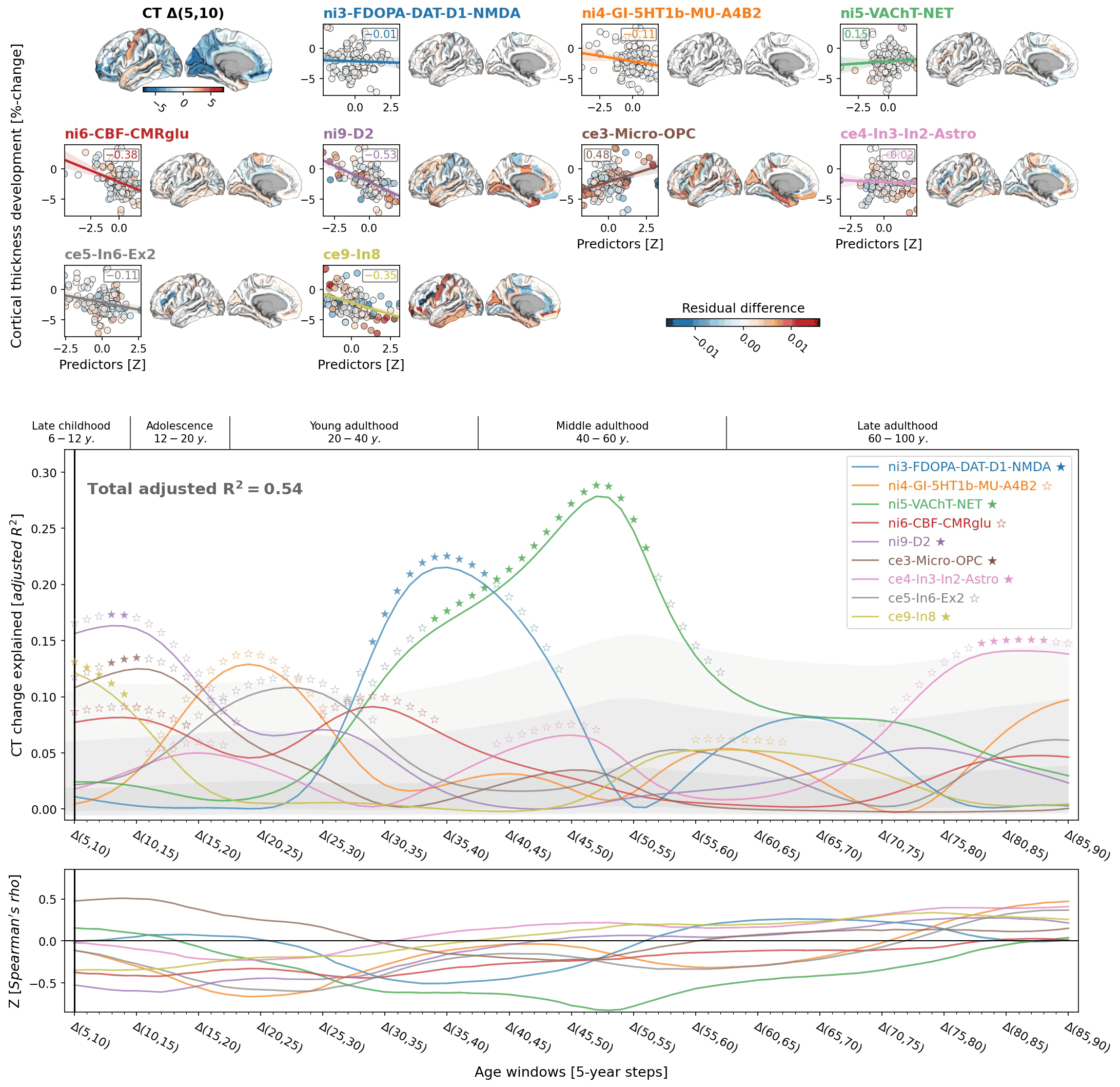

Supplement: Supplement 3 [file media-3.gif]
