# Supplementary material for: Human cortex development is shaped by molecular and cellular brain systems
Source: bioRxiv. 2023 May 5:2023.05.05.539537. Preprint. [Version 1] doi: 10.1101/2023.05.05.539537 (PMC10187287; doi:10.1101/2023.05.05.539537)
Supplement: Supplementary file 1 [file media-1.pdf]

Supplementary Materials

Human cortex development is shaped by molecular and cellular brain systems

Leon D. Lotter*, Amin Saberi, Justine Y. Hansen, Bratislav Misic, Gareth J. Barker, Arun L.W. Bokde, Sylvane Desrivières, Herta Flor, Antoine Grigis, Hugh Garavan, Penny Gowland, Andreas Heinz, Rüdiger Brühl, Jean-Luc Martinot, Marie-Laure Paillère, Eric Artiges, Dimitri Papadopoulos Orfanos, Tomáš Paus, Luise Poustka, Sarah Hohmann, Juliane H. Fröhner, Michael N. Smolka, Nilakshi Vaidya, Henrik Walter, Robert Whelan, Gunter Schumann, IMAGEN Consortium, Frauke Nees, Tobias Banaschewski, Simon B. Eickhoff, and Juergen Dukart

*Corresponding author: l.lotter@fz-juelich.de, leondlotter@gmail.com

This document contains:

Supplementary Text 1.1–2.1

Supplementary Figures S1–S21

Supplementary References

Supplementary Tables S1-S5 are provided in a separate Excel file.

Table of Contents

Abbreviations: 2

1. Supplementary Results 4

1.1. Molecular and cellular atlases of biological brain systems 4

1.2. Modeled CT data 4

1.3. Brain-regional contributions to CT association patterns 5

1.4. Evaluation of original in comparison to dimensionality-reduced multilevel atlases 5

1.5. ABCD and IMAGEN cohort demographics and quality control 6

1.6. Effects of sex and site on explained CT change 7

1.7. Effects of reference model predictive performance, subject-level deviations, and data quality on explained CT change 7

2. Supplementary Methods 8

2.1. Processing of Allen Human Brain Atlas mRNA expression data 8

3. Supplementary Figures 10

Fig. S1: Multimodal nuclear imaging and neural cell type atlases 11

Fig. S2: Dimensionality reduction of multimodal atlases 12

Fig. S3: Age distributions of the Braincharts cohort 14

Fig. S4: Lifespan cortical thickness development 15

Fig. S5: Influence of sex on spatial colocalization between multimodal predictors and lifespan cortical thickness change 16

Fig. S6: Joint multivariate regression result in different analysis cases 18

Fig. S7: Influence of sex, sliding window length, and cortical thickness reference percentile on main regression results 19

Fig. S8: Contribution of individual brain regions to explained lifespan cortical thickness change.......20

Fig. S9: Individual dominance analyses using original multimodal atlases prior to dimensionality reduction 21

Fig. S10: Cortical distributions of residual differences, cortical thickness changes, and average values of the original atlases associated to each selected predictor 22

Fig. S11: Euler number-based quality control of ABCD and IMAGEN data 23

Fig. S12: Observed and predicted ABCD and IMAGEN cohort-average cortical thickness change.....24

Fig. S13: Region-wise correlation between predicted and observed cortical thickness change 25

Fig. S14: Spatial colocalization between cross-sectional ABCD and IMAGEN cortical thickness data and multimodal brain systems.....27

Fig. S15: Detailed explained variance results of dominance analyses predicting ABCD and IMAGEN CT change from multilevel brain systems.....29

Fig. S16: Detailed Spearman correlation results showing colocalization patterns between ABCD and IMAGEN CT change and multimodal predictors.....31

Fig. S17: Generalization of CT change prediction models trained on normative single-subject data to observed data.....32

Fig. S18: Effects of follow-up duration on total explained cortical thickness change.....33

Fig. S19: Effects of sex and site on total explained cortical thickness change variance.....34

Fig. S20: Effects of predictive performance, subject-level atypical development, and surface reconstruction quality on explained cortical thickness.....35

Fig. S21: Summary of previous literature on processes involved in human brain development and maturation.....37

4. Supplementary References.....38

Abbreviations:

CT	cortical thickness
MRI	magnetic resonance imaging
mRNA	messenger ribonucleic acid
ABA	Allen brain atlas
MNI	Montreal Neurological Institute
FDR	false-discovery rate
ABCD	Adolescent Cognitive Brain and Development
ANCOVA	analysis of covariance
ni	nuclear imaging-derived brain atlas
ce	cell marker-derived brain atlas
mr	MRI-derived brain atlas
SV2A	synaptic vesicle glycoprotein 2A
M1	muscarinic receptor 1
mGluR5	metabotropic glutamate receptor 5
5HT1a/1b/2a/4/6	serotonin receptor 1a/2a/4/6

CB	cannabinoid receptor 1
GABAa	γ-aminobutyric acid receptor A
HDAC	histone deacetylase
5HTT	serotonin transporter
FDOPA	fluorodopa
DAT	dopamine transporter
D1/2	dopamine receptor 1/2
NMDA	N-methyl-D-aspartate glutamate receptor
GI	glycolytic index
MU	mu opioid receptor
A4B2	α4β2 nicotinic receptor
VAChT	vesicular acetylcholine transporter
NET	noradrenaline transporter
CBF	cerebral blood flow
CMRglu	cerebral metabolic rate of glucose
COX1	cyclooxygenase 1
H3	histamine receptor 3
TSPO	translocator protein
Ex	excitatory neurons
In	inhibitory neurons
Oligo	oligodendrocytes
Endo	endothelial cells
Micro	microglia
OPC	oligodendrocyte progenitor cells
Astro	astrocytes

1. Supplementary Results

1.1. Molecular and cellular atlases of biological brain systems

As indicators of biological systems potentially underlying CT changes, we collected 27 *in vivo* nuclear imaging atlases (20 neurotransmitter systems, cerebral glucose uptake, blood flow, aerobic glycolysis, synaptic density, transcriptomic activity, and two atlases capturing brain immune function), an MRI-derived atlas of cortical myelination, and 21 atlases of neuronal and glial cell types generated from Allen Brain Atlas mRNA expression data based on marker genes identified in adult human brain tissue (Tab. S1; Fig S1 & S2A) (Aghourian et al., 2017; Beliveau et al., 2017; Ding et al., 2010; Dukart et al., 2018; Finnema et al., 2016; Gallezot et al., 2010, 2017; Galovic et al., 2021; Gómez et al., 2018; Hawrylycz et al., 2012; Hillmer et al., 2016; Kaller et al., 2017; Kantonen et al., 2020; Kaulen et al., 2022; Kim et al., 2020; Lake et al., 2016; Lois et al., 2018; Markello et al., 2022; Naganawa et al., 2021; Normandin et al., 2015; Radhakrishnan et al., 2018; Sandiego et al., 2015; Smart et al., 2019; Vaishnavi et al., 2010; Wey et al., 2016). We performed factor analyses to reduce multicollinearity in the following regression analyses while retaining interpretability. All unrotated factors that explained at least 1% of variance of each dataset (nuclear imaging vs. neural cell types) were retained, resulting in 10 nuclear imaging factors and 10 cell type factors (named *ni1*–*ni10*, and *ce1*–*ce10*; Fig. S2B–E). After promax rotation, nuclear imaging factors explained 90.9% and cell type factors explained 86.9% of each respective dataset. Adding the MRI-derived myelin atlas (*mr1*), 21 factors were used as "predictors" for the following analyses (Fig. 2A).

1.2. Modeled CT data

Reference CT values across the lifespan for 148 cortex regions were extracted from the *Braincharts* normative model published by Rutherford et al. (2022) (5–90 years with 0.5-year steps; separate female and male data from approximately 58,000 subjects; 1st, 5th, 25th, 50th, 75th, 95th, and 99th model percentile, age distribution: Fig. S3). As expected, lifespan cortical thickness development showed a general trend towards cortical thinning, with different trajectories across brain regions. While absolute values differed slightly by sex, the general trajectories and relative development were highly similar, so that we decided to average the across sexes for all main analyses (Fig. S4A, Anim. S1).

Cross-sectional colocalization analyses were based on the modeled CT data at each extracted timepoint. For analyses aiming to explain modeled longitudinal CT change patterns, we

calculated timepoint-to-timepoint relative CT change of 50th percentile data within a sliding window approach (5–90-year range, 5-year length, and 1-year steps). During the child-to-adulthood period, precentral and temporal gyri showed the strongest relative increase of CT while the remaining cortex showed thinning patterns. Generally, the strongest CT changes across the lifespan (mostly thinning) occurred in the first and last third of life (Fig. S4B–C).

1.3. Brain-regional contributions to CT association patterns

We evaluated brain-regional contributions to the overall explained CT change by calculating the residual difference for each brain atlas as the difference in prediction errors resulting from a multivariate regression with and without the brain atlas included as predictor. Generally, medial occipital, medial temporal, sensorimotor, and cingulate cortices influenced the explained CT change patterns strongly. For *ce9-In8*, the premotor cortex, cuneus, and multiple frontopolar sulci were the most influential regions. *Ce3-MicroOPC* showed a similar pattern, while *ni9-D2* showed the strongest residual differences in the middle cingulate cortex, precuneus, insula, and temporal pole. The two metabolism factors (*ni4* and *ni6*) showed less pronounced patterns with generally occipitotemporal regions having the strongest influences. The two brain systems relevant for midlife CT change, *ni3-FDOPA-DAT-D1-NMDA and ni5-VAChT-NET*, again displayed a high relevance of lateral and medial somatosensory cortices as well as the precuneus, middle to anterior cingulate, and medial temporal regions. Fig. 4 shows the respective patterns at each system's maximum explained CT change, Anim. S2 illustrates how the observed patterns develop longitudinally, and Fig. S9 provides an overview for the complete lifespan.

1.4. Evaluation of original in comparison to dimensionality-reduced multilevel atlases

To demonstrate that the factor-level atlases were appropriately representing the original multi-level brain atlases, we performed one additional set of dominance analyses for each factor-level atlas, using the 5 original atlases with the highest factor loadings if the absolute loading exceeded > 0.3. Except for *ce4-In3-In2-Astro*, all factors explained CT change significantly (nominal $p < 0.05$). In all cases, the total explained CT change R^2 peaks arising from each original atlas set occurred at the same time in life as observed for the factor-level atlases. For some factor-level atlases, we discovered that their peak contribution to explained CT change was driven by a certain original atlas, while other original atlases were of lesser relevance. For *ni3-FDOPA-DAT-D1-NMDA*, we showed that the midlife peak was mostly driven by NMDA and DAT. Other strongly loading atlases – DOPA, D1, and NET – explained more CT change before 25 and after 50 years.

The contribution to early explained CT change of *ni4-GI-5HT1b-MU-A4B2* was driven by GI, capturing aerobic glycolysis. For *ni5-VACht-NET*, VAChT indeed accounted for most of the CT change explained by the factor-level atlas during midlife. Furthermore, the A4B2 nicotinic receptor contributed to this factor. The relevance of *ni9-D2* for early explained CT change was indeed driven by the D2 receptor, however the D1 receptor additionally loaded on the factor and accounted for around 15% of explained early CT change. For *ce3-Micro-OPC*, the microglia distribution contributed more strongly, although explaining less CT change in comparison to other predictors. Finally, *ce5-In6-Ex2* was dominated by Ex2 (layer 3/4 granule neurons), and we observed an additional contribution of Ex3 (layer 4 granule neurons) to *ce9-In8*. Fig. S10 illustrates the detailed results, Fig. S11 shows the peak region-wise residual differences for each original atlas.

1.5. ABCD and IMAGEN cohort demographics and quality control

We obtained single-subject CT data from the ABCD (Casey et al., 2018) and the IMAGEN (Schumann et al., 2010) cohort studies to validate our findings. Data quality was ensured based on the manual ratings included in the ABCD dataset and on FreeSurfer's "number of surface defects" metric. Tab. S2 lists age and sex distributions for both cohorts and each timepoint. Fig. S12 shows the quality control metric distributions.

Regarding the ABCD dataset, initially, baseline data for 11,760 subjects and 2-year follow-up data for 7,829 subjects was available. After dropping one study site without longitudinal data and subjects with missing CT data, 11,716 and 7,818 datasets were retained. Subjects with low data quality were excluded, leading to 10,697 and 6,789 subjects. 420 subjects (20 per study site) that only had baseline data were used to adapt the Braincharts reference model to the study sites and to obtain site-adjusted CT values as well as individual deviations from the 50th model percentile (Bayer et al., 2021; Rutherford et al., 2021). Only subjects with longitudinal data were included in analyses, resulting in a final dataset of n = 6,789.

Concerning the IMAGEN dataset, 4,990 observations from 2,158 subjects were initially available. For 3,975 observations from 1,528 subjects, structural MRI data was available and successfully FreeSurfer-processed. After exclusion of subjects for quality reasons, 3,732 observations from 1,522 subjects were retained. Baseline data from 160 subjects (20 per site) was used for model adaptation and these subjects were excluded from analyses completely, resulting in a dataset of n = 1362 subjects to be used in analyses.

1.6. Effects of sex and site on explained CT change

After having assessed how CT development on the single-subject level was explained from multi-level brain systems, we evaluated whether the amount to which it was explained, varies with sex and study site. ANCOVA models corrected for follow-up duration showed significantly more explained CT change in males as compared to females only in the IMAGEN dataset (timespans T0–T5 and T0–T8), but not in the ABCD data. Furthermore, we observed effects of site on explained CT change in ANCOVA models corrected for sex and follow-up duration in both datasets (all timespans). Effects of study site were also observed for cross-sectional CT as well as CT change (Tab. S3–4) in several cortex regions. Given that the main goal of the current analysis was to establish the feasibility of capturing associations between CT development and multi-level brain systems on the individual level, clarification of the sources of sex- and site-effects will be a task for future investigations. Fig. S19 visualizes the group differences shows ANCOVA results.

1.7. Effects of reference model predictive performance, subject-level deviations, and data quality on explained CT change

We conducted correlational analyses to provide general indications of which factors influenced the extents to which CT change was explained in single-subject data. First, CT change patterns of subjects who had more "normative" CT patterns at *baseline* (i.e., stronger correlation between observed and Braincharts-predicted baseline CT) were not consistently better explained. In contrast, subjects whose CT change patterns were more in line with the *change* patterns predicted by the Braincharts model showed higher explained CT change. Similarly, while we did not observe consistent relationships between metrics capturing how a subject deviated from the model-predictions (count of deviation Z-scores > 2 and average absolute Z scores) and explained CT change, the longitudinal change in deviation metrics showed an association in all IMAGEN timespans: As expected, most subjects did not show strong changes in their deviations between timepoints, but subjects who showed *more or stronger deviations* at follow-up as compared to baseline tended towards *more explained CT change*. Whether such patterns could represent a potentially pathophysiological involvement of a certain biological system in neurodevelopment remains to be investigated. Finally, we observed that less CT change was explained in subjects with more surface defects (i.e., worse reconstruction quality) at follow-up (ABCD and IMAGEN) or at baseline (ABCD only). Fig. S20 shows the reported association patterns and provides Spearman correlation statistics.

2. Supplementary Methods

2.1. Processing of Allen Human Brain Atlas mRNA expression data

Regional microarray expression data were obtained from 6 post-mortem brains (1 female, age range 24.0–57.0 years, mean age 42.50 ± 13.38 years) provided by the Allen Human Brain Atlas (https://human.brain-map.org) (Hawrylycz et al., 2012). Data were processed with the abagen toolbox (version 0.1.3; https://github.com/rmarkello/abagen) (Markello et al., 2021) using a 148-region surface-based atlas in fsaverage5 space (Destrieux et al., 2010).

First, microarray probes were reannotated using data provided by Arnatkevičiūtė et al. (2019); probes not matched to a valid Entrez ID were discarded. Next, probes were filtered based on their expression intensity relative to background noise (Quackenbush, 2002), such that probes with intensity less than the background in $\geq 50\%$ of samples across donors were discarded, yielding 31,569 probes. When multiple probes indexed the expression of the same gene, we selected and used the probe with the most consistent pattern of regional variation across donors [i.e., differential stability (Hawrylycz et al., 2015)], calculated with:

$$\Delta_S(p) = \frac{1}{\binom{N}{2}} \sum_{i=1}^{N-1} \sum_{j=i+1}^{N} \rho\left[B_i(p), B_j(p)\right]$$

where p is Spearman's rank correlation of the expression of a single probe, p, across regions in two donors B_i and B_j, and N is the total number of donors. Here, regions correspond to the structural designations provided in the ontology from the Allen Human Brain Atlas. The MNI coordinates of tissue samples were updated to those generated via non-linear registration using the Advanced Normalization Tools (ANTs; https://github.com/chrisfilo/alleninf). To increase spatial coverage, tissue samples were mirrored bilaterally across the left and right hemispheres [R2018N]. Samples were assigned to brain regions by minimizing the Euclidean distance between the MNI coordinates of each sample and the nearest surface vertex. Samples where the Euclidean distance to the nearest vertex was more than 2 standard deviations above the mean distance for all samples belonging to that donor were excluded. To reduce the potential for misassignment, sample-to-region matching was constrained by hemisphere and gross structural divisions [i.e., cortex, subcortex/brainstem, and cerebellum, such that e.g., a sample in the left cortex could only be assigned to an atlas parcel in the left cortex; (Arnatkevičiūtė et al., 2019)]. All tissue samples not assigned to a brain region in the provided atlas were discarded. Inter-subject variation was

addressed by normalizing tissue sample expression values across genes using a robust sigmoid function (Fulcher et al., 2013):

$$x_{norm} = \frac{1}{1 + exp - \left(\frac{(x - \langle x \rangle)}{IQR_x}\right)}$$

where $\langle x \rangle$ is the median and IQR_x is the normalized interquartile range of the expression of a single tissue sample across genes. Normalized expression values were then rescaled to the unit interval:

$$x_{scaled} = \frac{x_{norm} - \min(x_{norm})}{\max(x_{norm}) - \min(x_{norm})}$$

Gene expression values were then normalized across tissue samples using an identical procedure. Samples assigned to the same brain region were averaged separately for each donor and then across donors, yielding a regional expression matrix with 148 rows, corresponding to brain regions, and 15,633 columns, corresponding to the retained genes.

3. Supplementary Figures

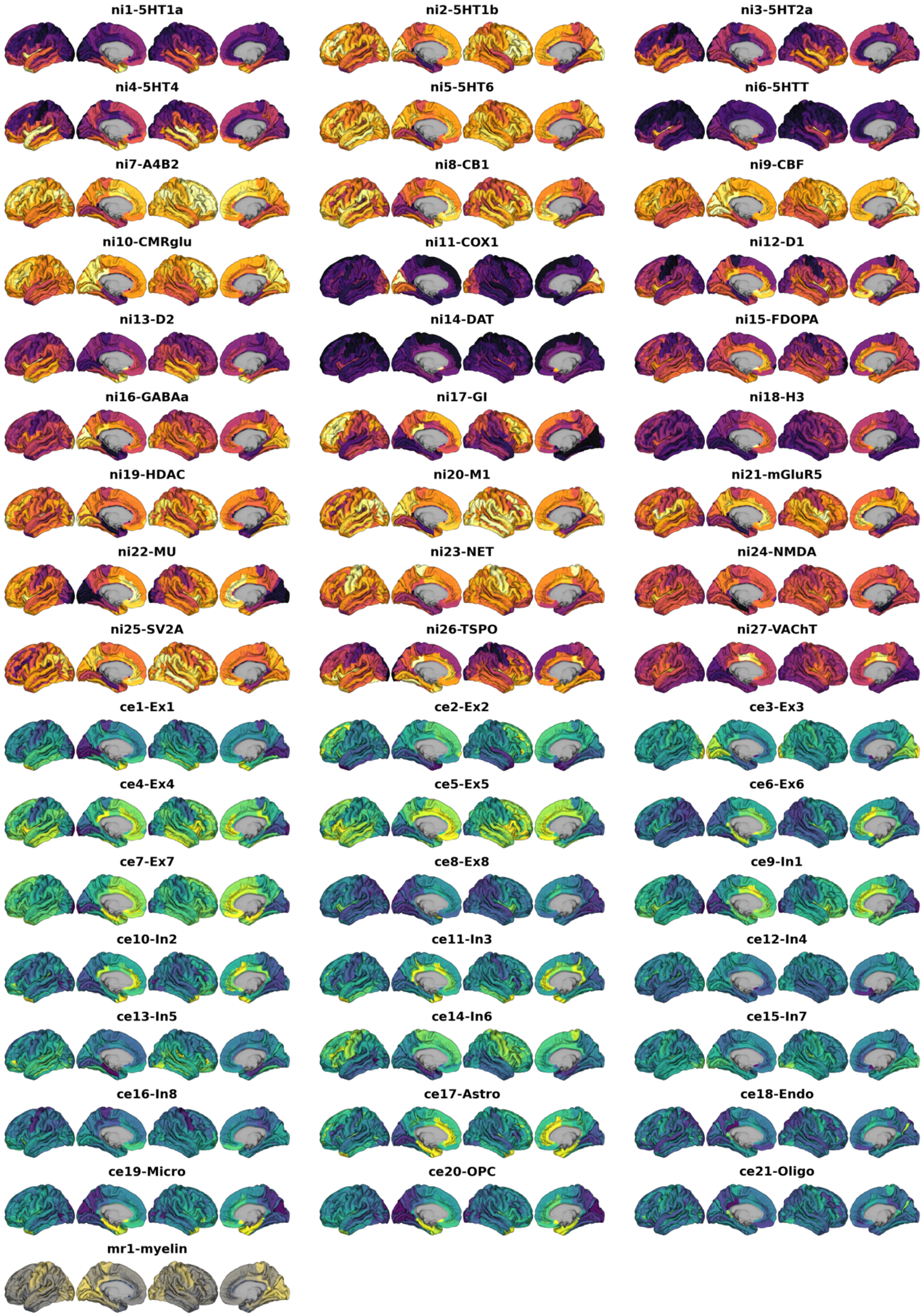

Fig. S1: Multimodal nuclear imaging and neural cell type atlases

Multimodal atlases after transformation to FreeSurfer space and parcellation into 148 cortical parcels (Destrieux parcellation). Nuclear imaging maps are colored orange-violet, gene expression maps yellow-green, and the myelin map is colored yellow-grey. See Table S1 for individual descriptions and sources.

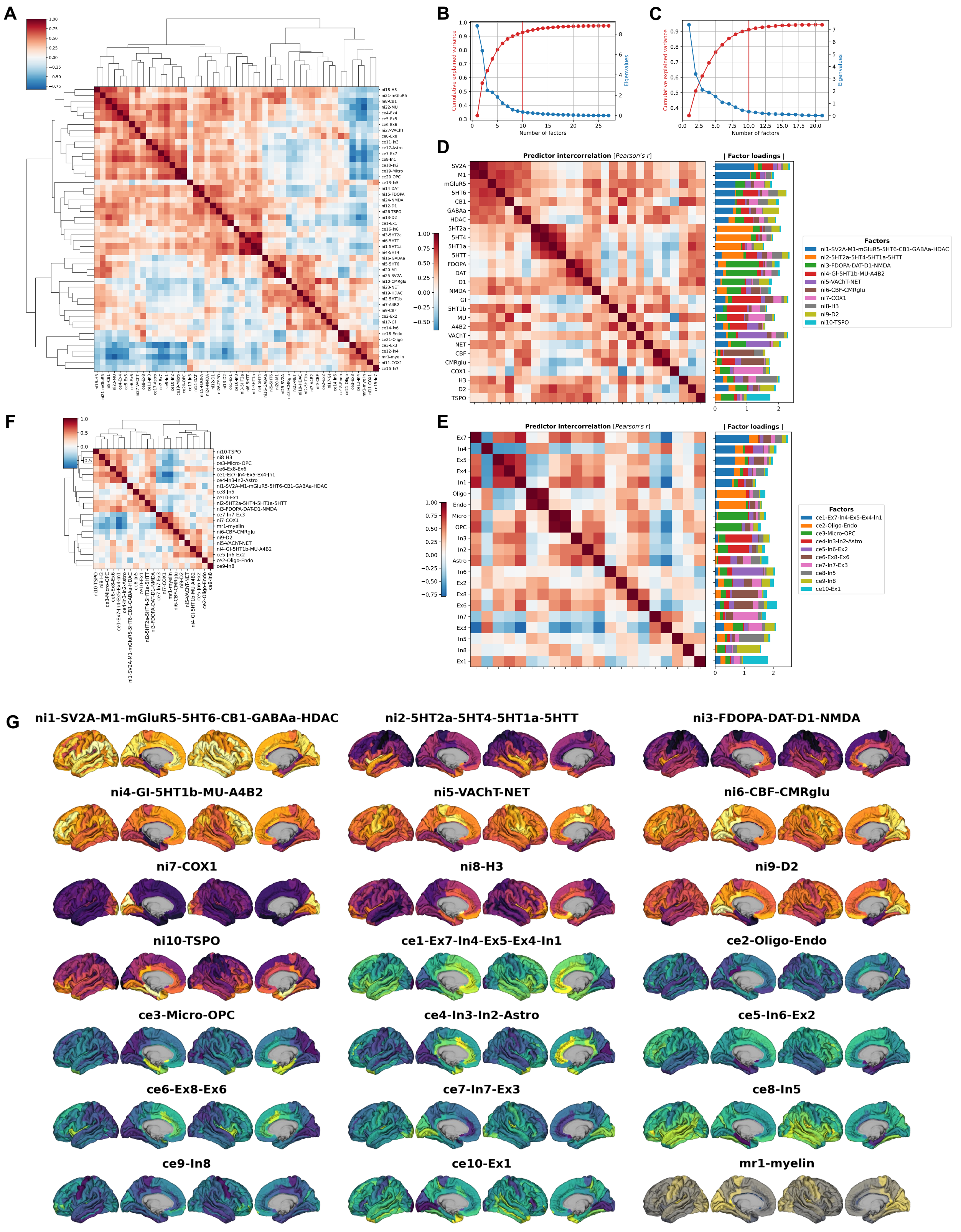

Fig. S2: Dimensionality reduction of multimodal atlases

A: Spearman correlation matrix of original multimodal atlases. B & C: Cumulative explained variance (red) and eigenvalues (blue) of unrotated factors in a minimum residual factor analysis on the nuclear imaging atlases (B) and mRNA expression atlases (C). The red vertical line marks the threshold of factors explaining at least 1% of variance.

D & E: Factors extracted from the nuclear imaging (D) and mRNA expression (E) datasets after promax rotation. The heatmaps show Pearson correlations between the original atlases before factor analysis. Stacked barplots show factor loadings for each original atlas on each factor. Factor names were derived from assigning each original atlas to the factor it loaded on most so that each original atlas appears exactly once in the overall factor names. F: Spearman correlation matrix of the 20 derived factors and the myelin atlas. G: Parcellated brain atlases after dimensionality reduction mapped to the cortex (see Fig. S1 for all original atlases). Nuclear imaging factors are colored orange-violet, gene expression factors yellow-green, and the myelin map is colored yellow-grey.

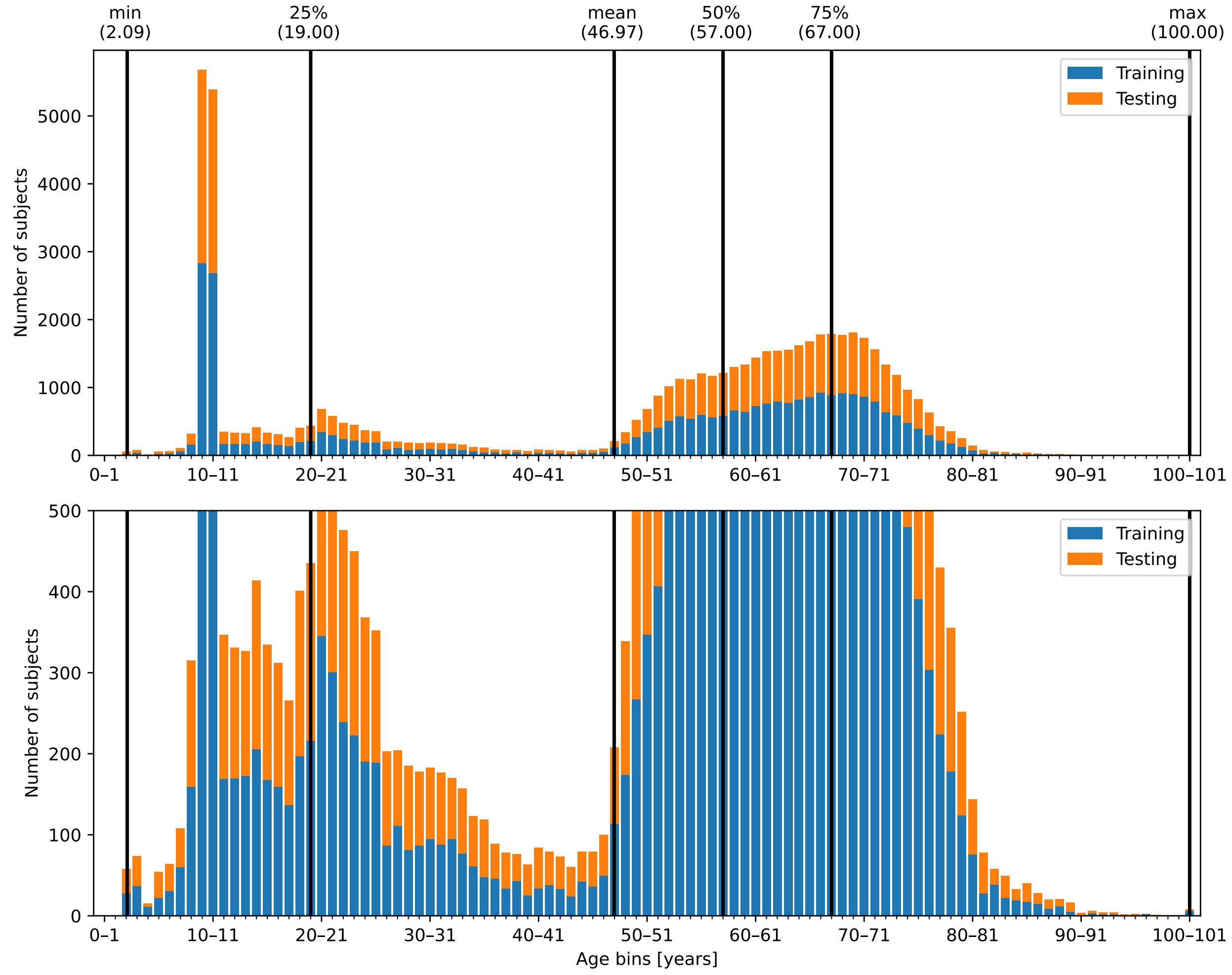

Fig. S3: Age distributions of the Braincharts cohort

Age distribution in Braincharts model, displayed as stacked histograms (blue: training data, orange: testing data) with 1-year bins (bin "0–1": ≥ 0 to < 1 years, bin "1–2": ≥ 1 to < 2 years, …). The lower panel is a copy of the upper panel with scaled y axis to visualize smaller bins. Black vertical lines show descriptive statistics for the whole dataset. The sharp peak at 9 to 11 years is caused by the ABCD study dataset, the smoother peak at 50 to 75 years is related to the UK Biobank study.

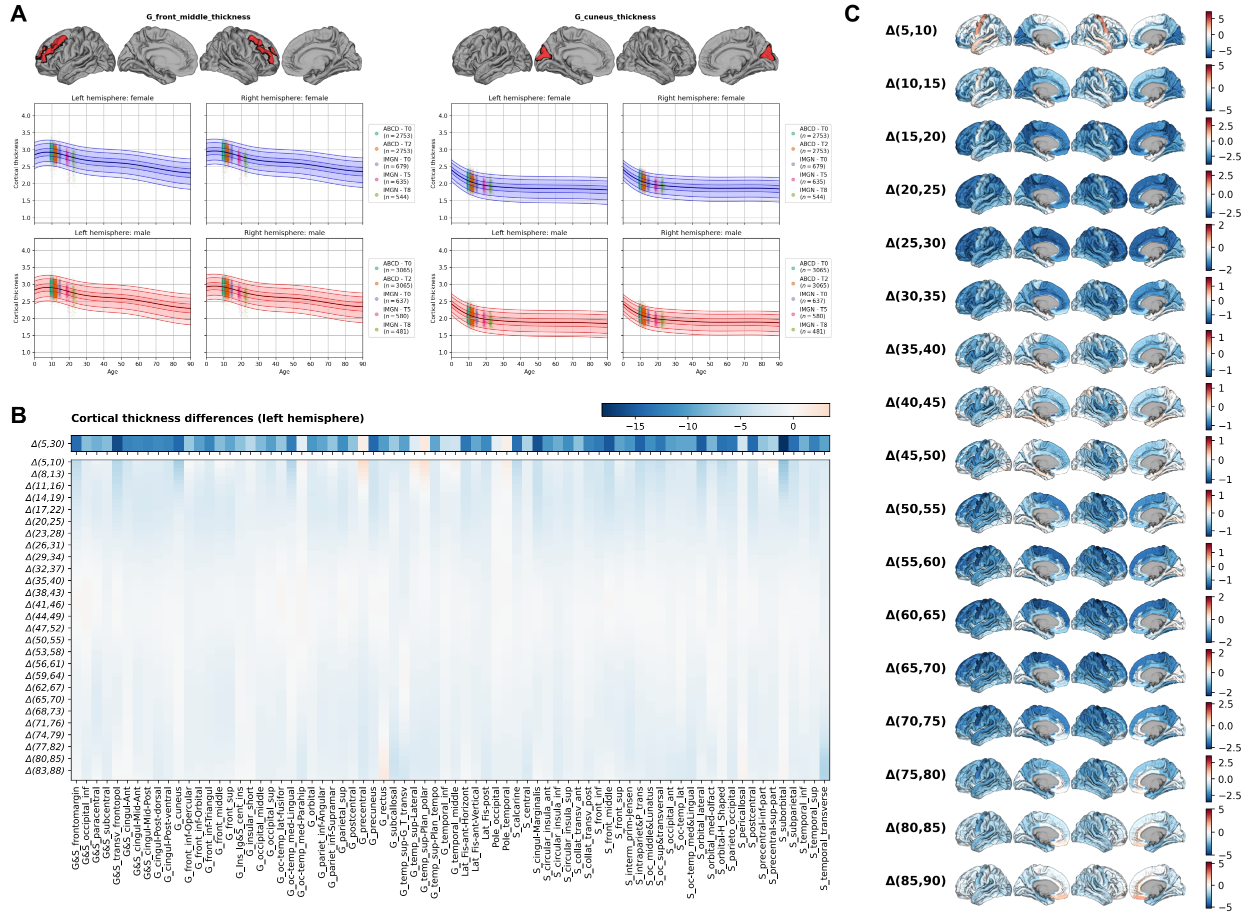

Fig. S4: Lifespan cortical thickness development

Cortical thickness data extracted from the reference model by Rutherford et al. (2022) and the IMAGEN cohort (Schumann et al., 2010). A: Examplary developmental trajectories of cortical thickness of two single bilateral brain regions for females (blue) and males (red). The darkest line represents the median, the brighter lines show the .01th, .05th, .25th as well as the .75th, .95th, and .99th centiles. Scatters show ABCD and IMAGEN subjects after adaptation to the Braincharts model at each study time points. See Animation S1 for all brain regions. B: Left-hemispheric region-wise relative cortical thickness differences in percent-change from 5 to 30 years (upper row) and from 5 to 90 years, estimated using a sliding window with 1-year steps and 5-year window length (lower subfigure). C: Cortical thickness development in 5-year steps, plotted are region-wise percent-change values.

A: Female

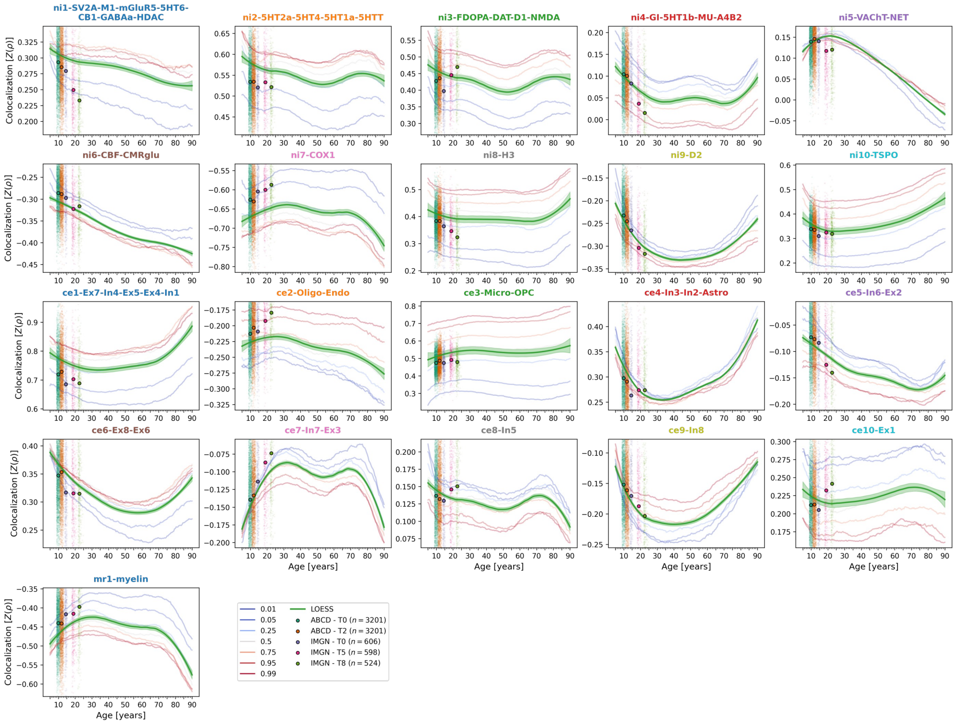

B: Male

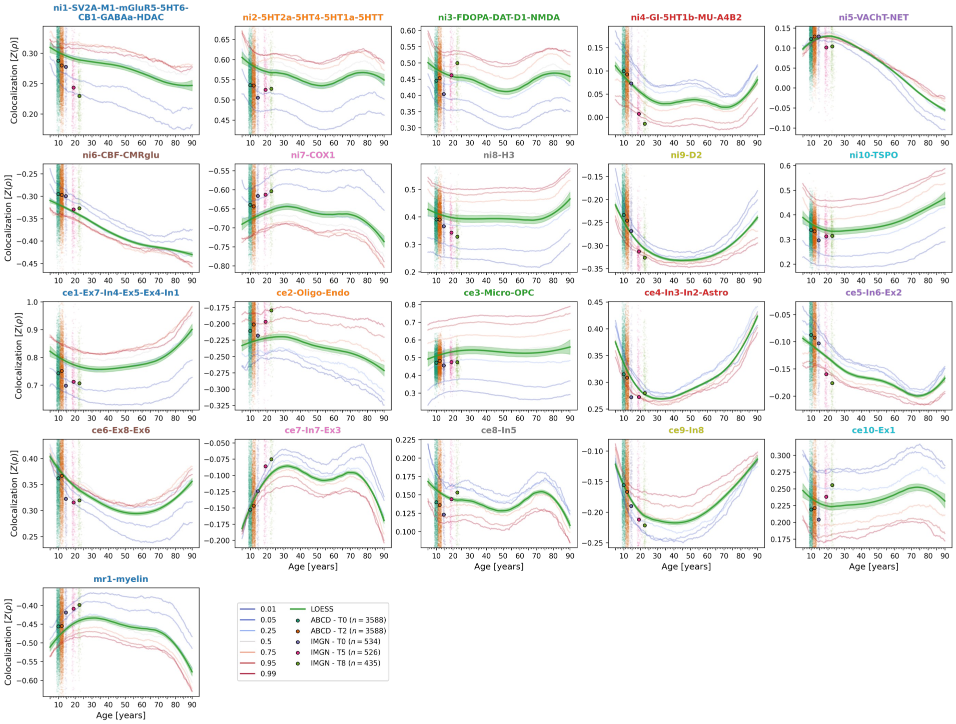

Fig. S5: Influence of sex on spatial colocalization between multimodal predictors and lifespan cortical thickness change

A: Development of CT-predictor colocalization in female data. B: Colocalization in male data. Please refer to Fig. 2 for descriptions of the panel elements.

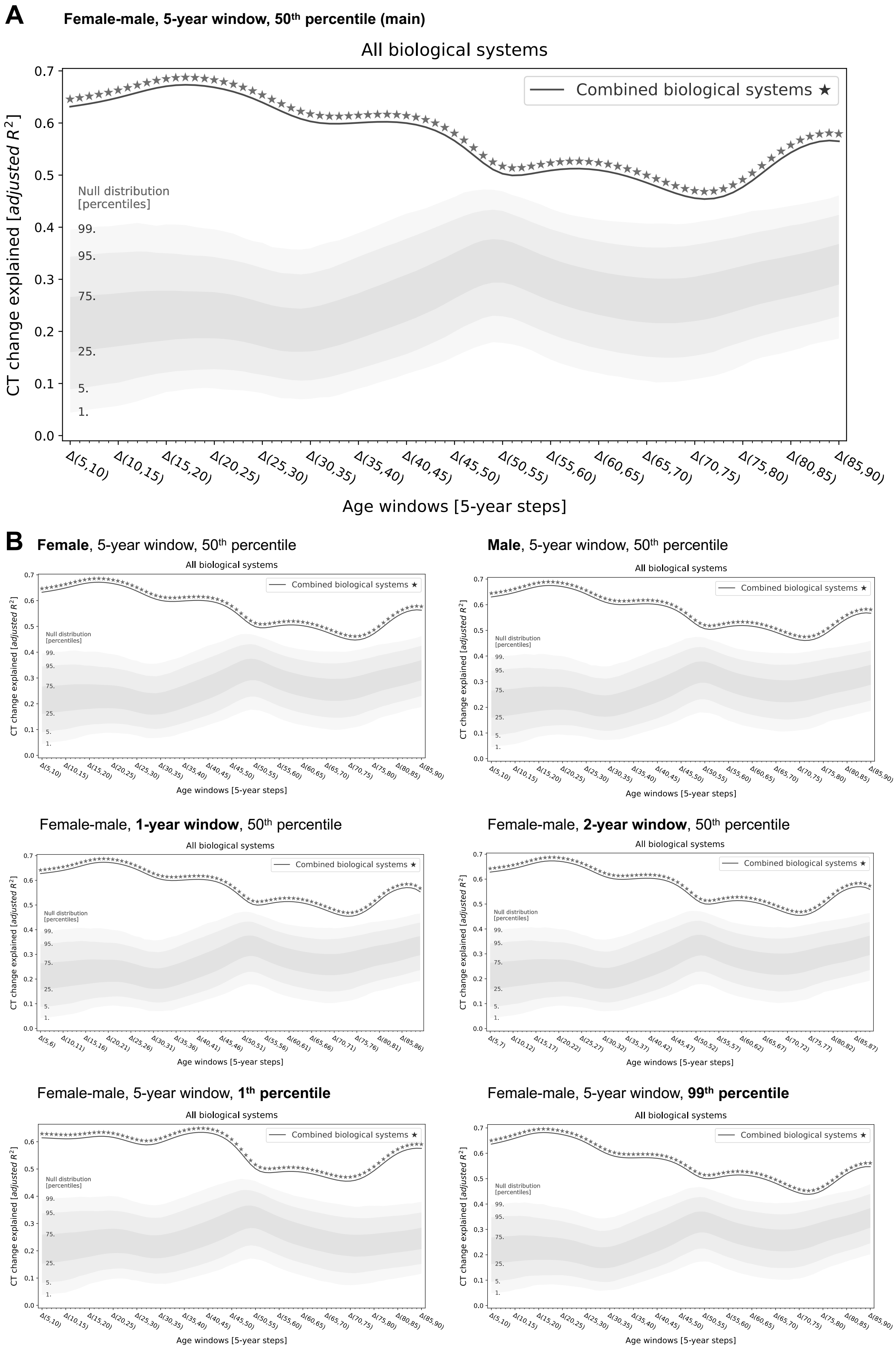
A
Female-male, 5-year window, 50th percentile (main)
All biological systems
Combined biological systems ★
Null distribution [percentiles]
99.
95.
75.
25.
5.
1.
CT change explained [adjusted R^2]
Age windows [5-year steps]
B
Female, 5-year window, 50th percentile
Male, 5-year window, 50th percentile
Female-male, 1-year window, 50th percentile
Female-male, 2-year window, 50th percentile
Female-male, 5-year window, 1th percentile
Female-male, 5-year window, 99th percentile

Fig. S6: Joint multivariate regression result in different analysis cases

A: Multivariate regression on cortical thickness change across the lifespan using all predictors, neuroimaging and mRNA expression combined. B: "Joint" multivariate regression results for the following cases: (i) only male or female cortical thickness reference data, (ii) sliding window length of 1 or 2 years instead of 5 years, and (iii) first or 99th instead of 50th percentile cortical thickness reference data. Please refer to Fig. 3 for descriptions of the panel elements.

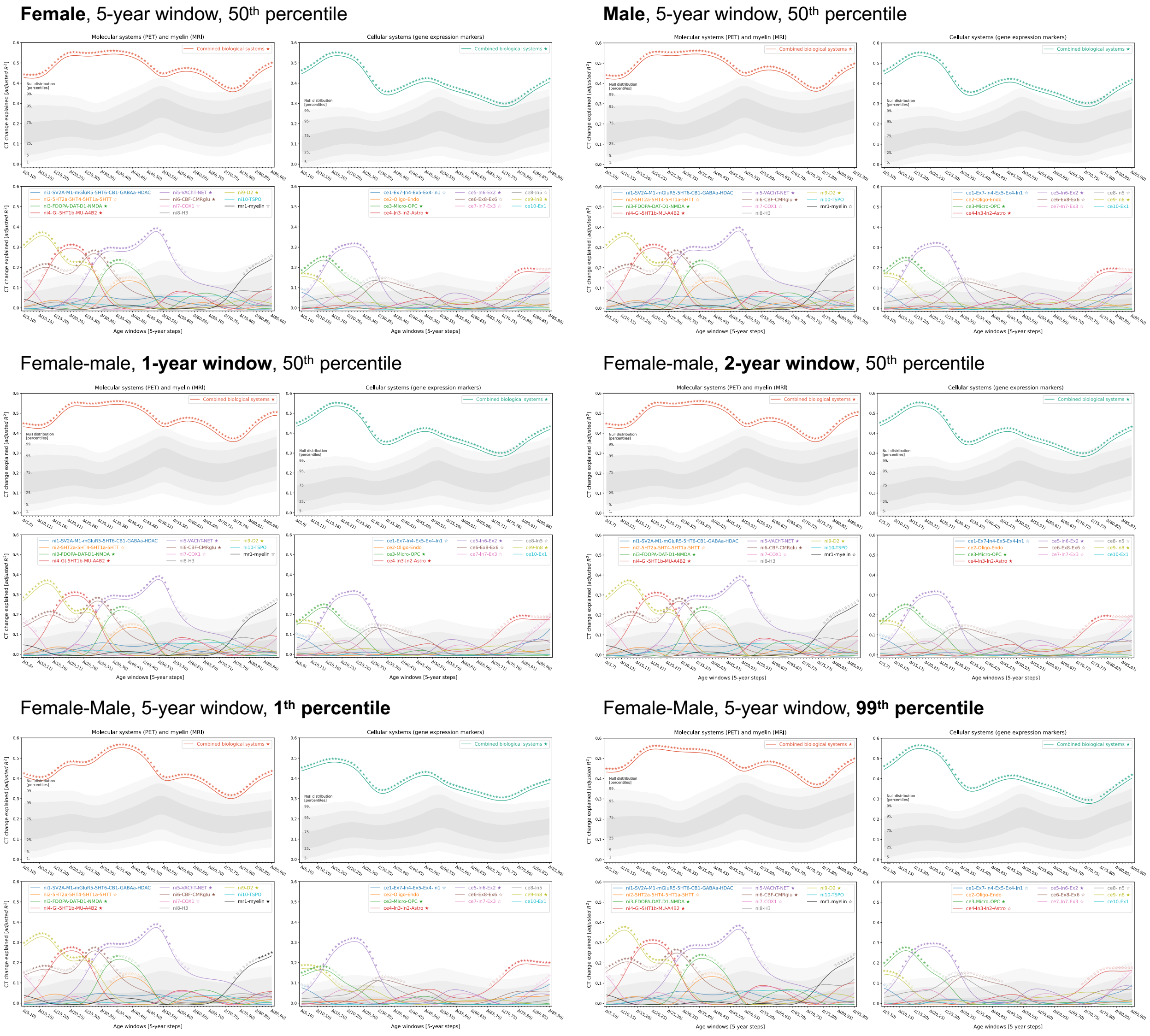

Fig. S7: Influence of sex, sliding window length, and cortical thickness reference percentile on main regression results

Univariate and multivariate regression results for the following cases: (i) only male or female cortical thickness reference data, (ii) sliding window length of 1 or 2 years instead of 5 years, and (iii) first or 99th instead of 50th percentile cortical thickness reference data. Please refer to Fig. 3 for descriptions of the panel elements.

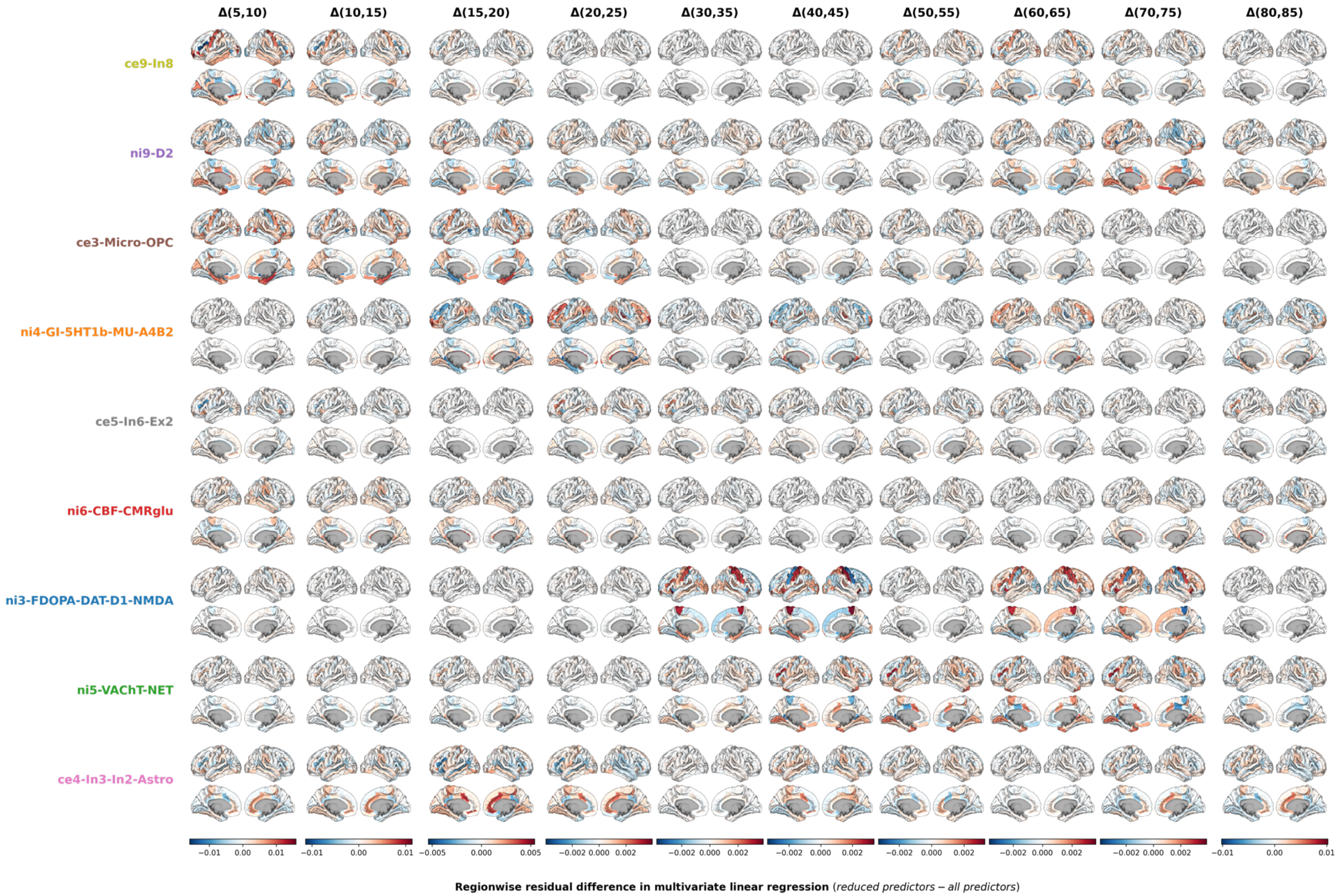

Fig. S8: Contribution of individual brain regions to explained lifespan cortical thickness change

Parcellated brain plots show residual differences as estimated in the main dominance analyses. Residual differences were calculated for each predictor x as the difference between prediction errors resulting from a multivariate linear regression with all 7 predictors included and the prediction errors under exclusion of x.

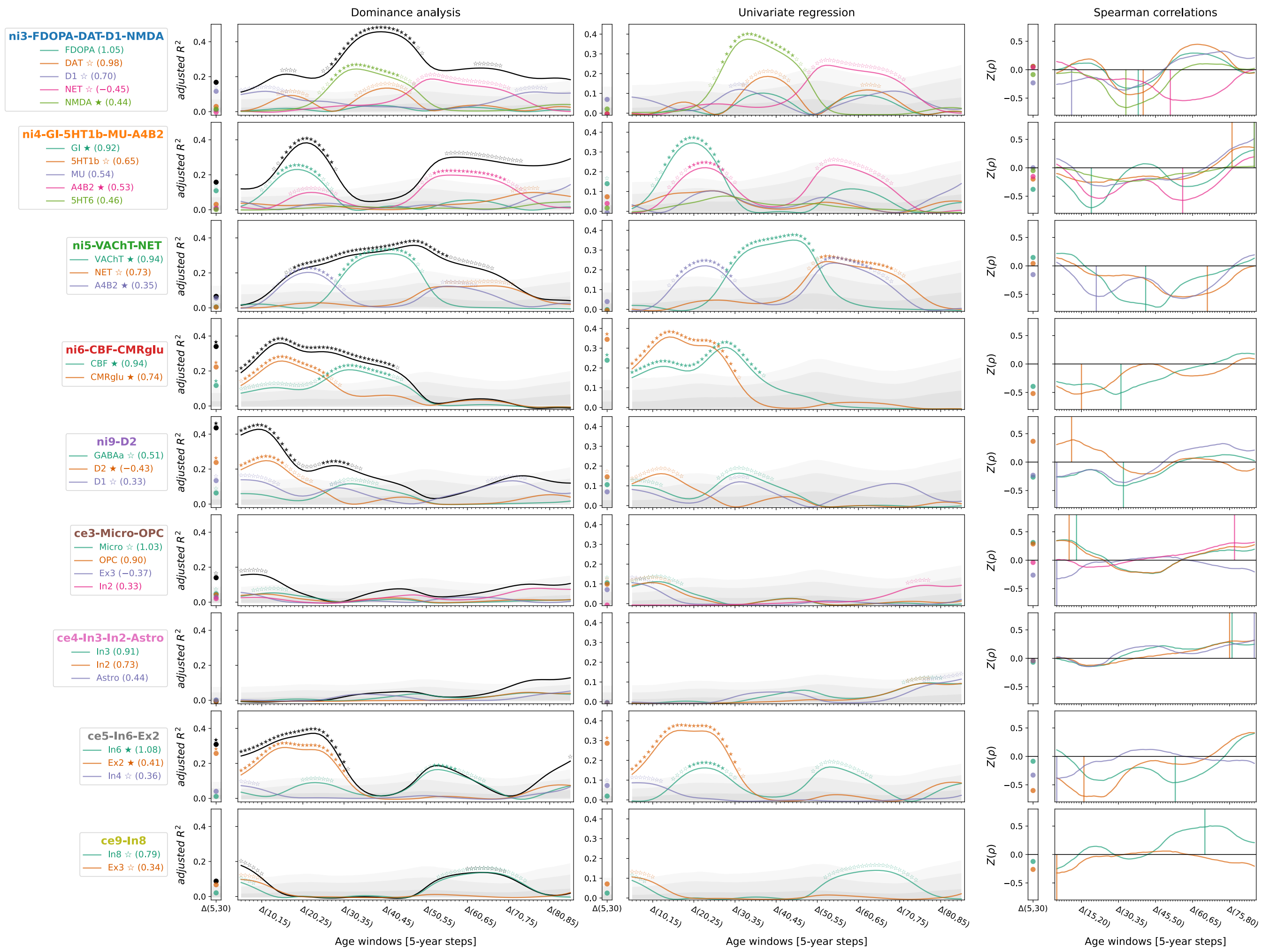

Fig. S9: Individual dominance analyses using original multimodal atlases prior to dimensionality reduction

To determine if the factor-level predictors appropriately captured the original multimodal atlases, sets of spatial association analyses were calculated, predicing CT change across the lifespan from the original maps most closely associated to each factor. For each factor, the 5 original atlases that loaded most strongly on the factor were selected given that their loading exceeded a threshold of 0.3. The first column shows the result of step-wise dominance analyses (black line = combined R^2), the second column shows independent single linear regressions, and the third column depicts the colocalization pattern between CT change and original predictors to illustrate the sign of the spatial association. Gray shades in the first two columns show the null distributions associated with the individual system-level R^2 values.

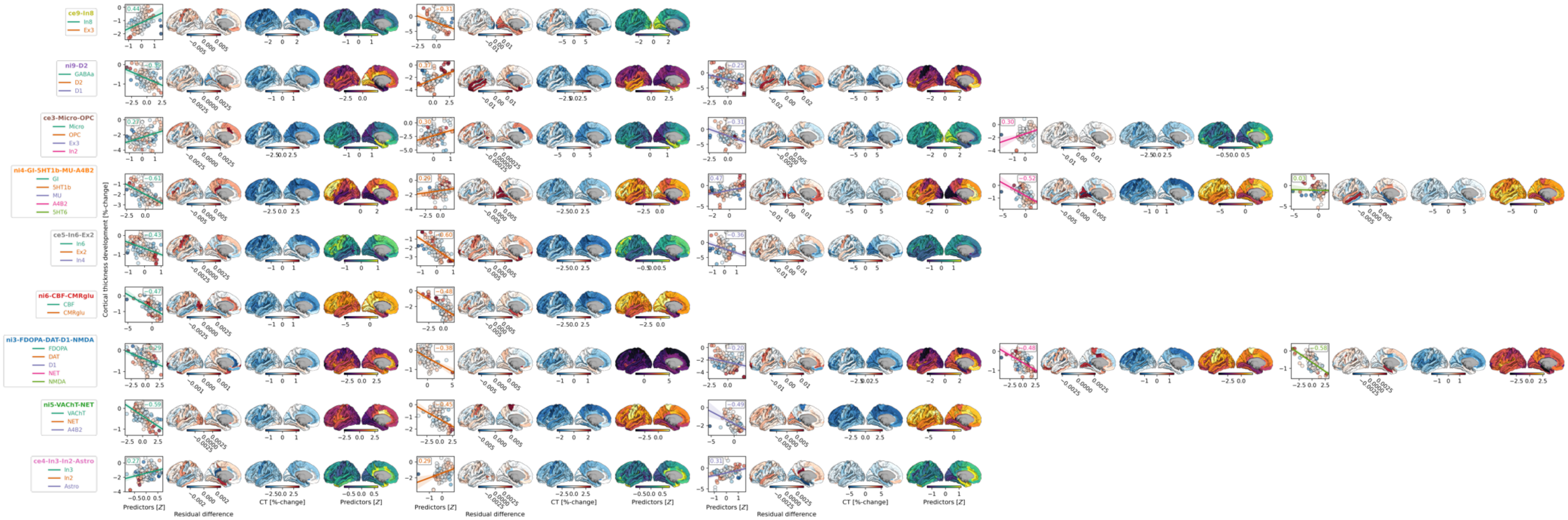

Fig. S10: Cortical distributions of residual differences, cortical thickness changes, and average values of the original atlases associated to each selected predictor

This figure corresponds to each row of Fig. S10 and mirrors the layout of Fig. 4B. Please see Fig.s S11 and 4B for descriptions of the panel elements.

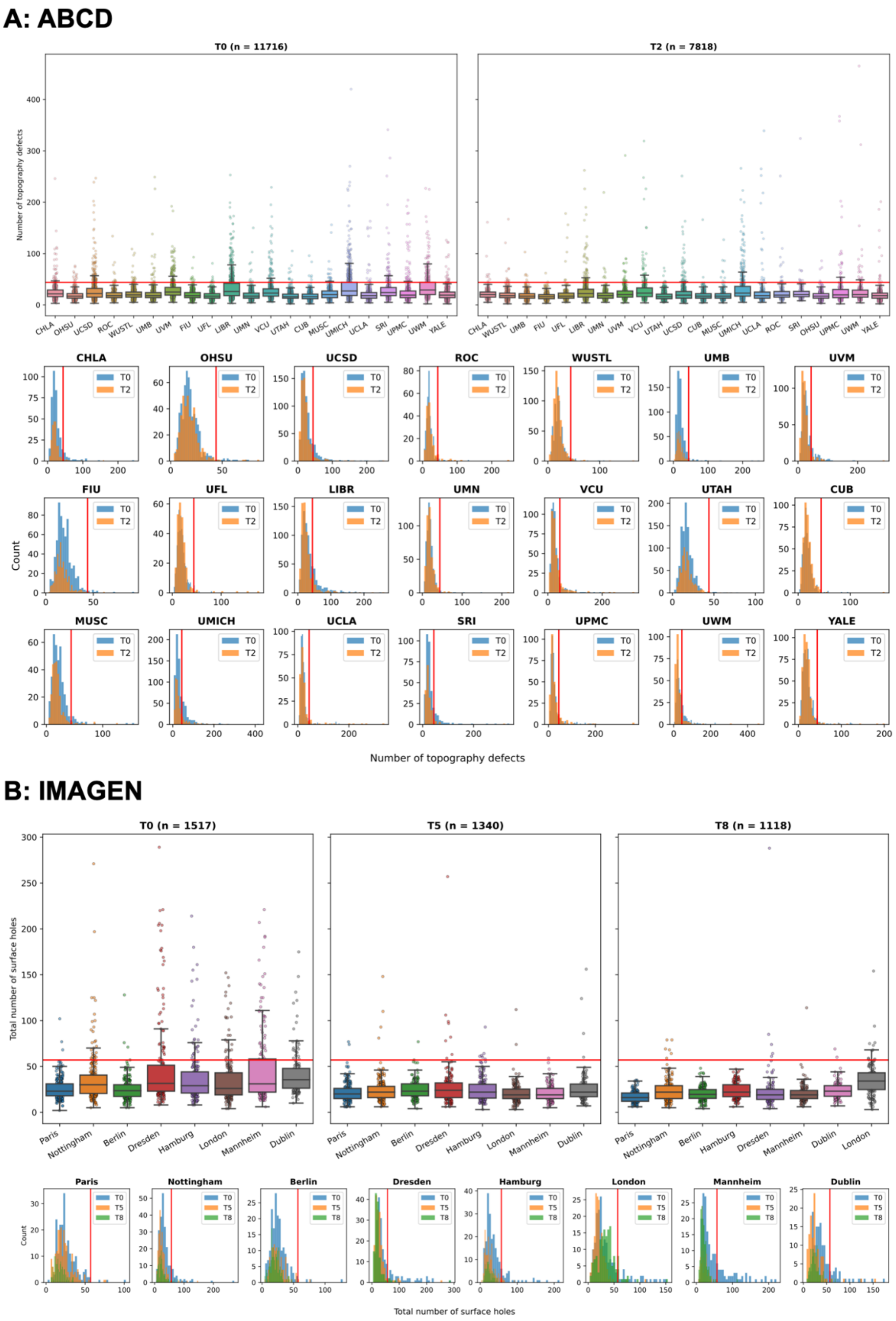

Fig. S11: Euler number-based quality control of ABCD and IMAGEN data

Distributions of Euler number-related metrics in ABCD (A) and IMAGEN (B) datasets at different study time points. The upper and lower sub-panels of A and B visualize the distributions of the quality control metrics in different ways. For IMAGEN, the sum of the two hemispheric Euler numbers, and for ABCD, the variable “apqc_smri_topo_ndefect” was used. Subjects were excluded if they exceeded a threshold of Q3 + 1.5 * IQR calculated across study time points within each study.

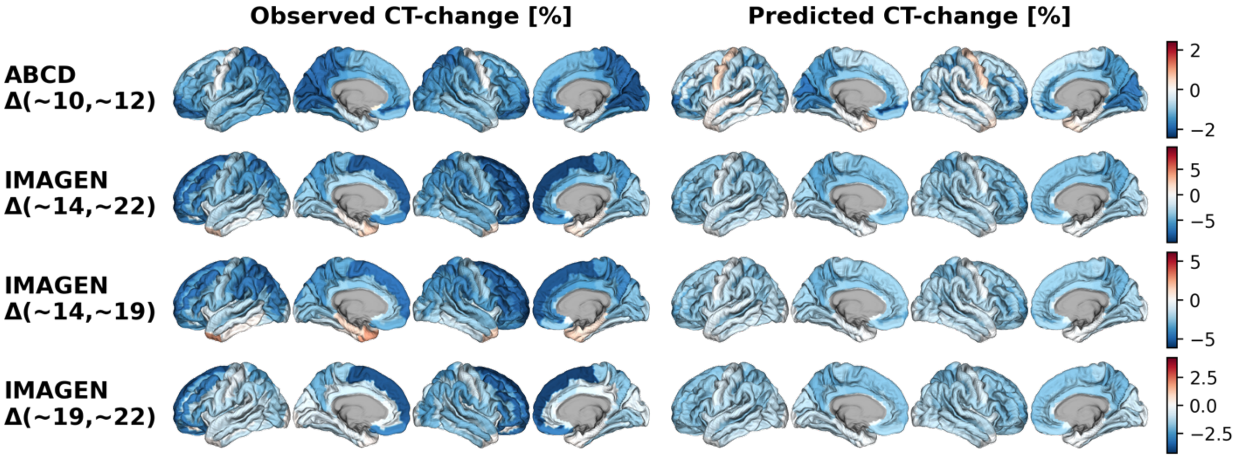

Fig. S12: Observed and predicted ABCD and IMAGEN cohort-average cortical thickness change

Average relative CT change in percent across four time periods in the ABCD and IMAGEN datasets as observed (left side) and as predicted by the Braincharts model based on age and sex of the subjects.

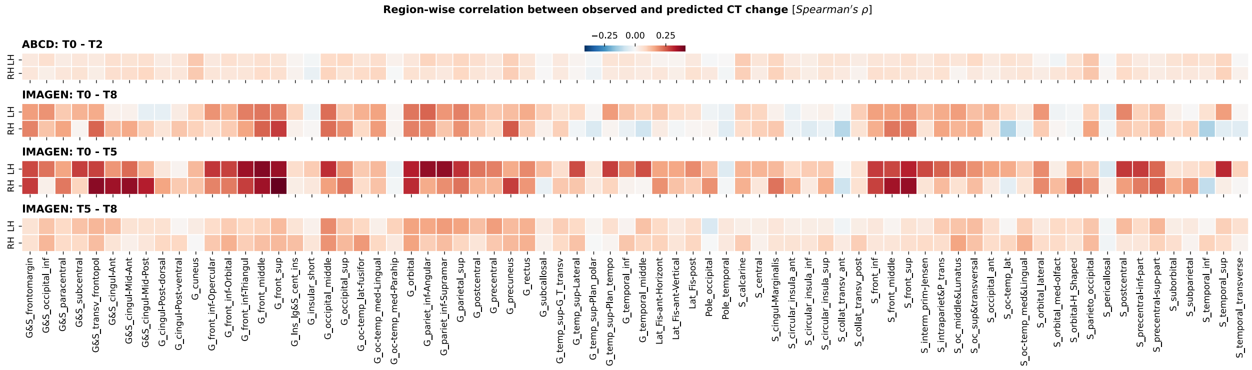

Fig. S13: Region-wise correlation between predicted and observed cortical thickness change

Regional correlation (Spearman's rho) between relative CT change as predicted by the Braincharts model (percent-change of predictd CT values from timepoint one to timepoint two) and relative CT change as observed in ABCD and IMAGEN datasets across each time period. Predicted CT change was calculated for each subject individually based on their age and sex.

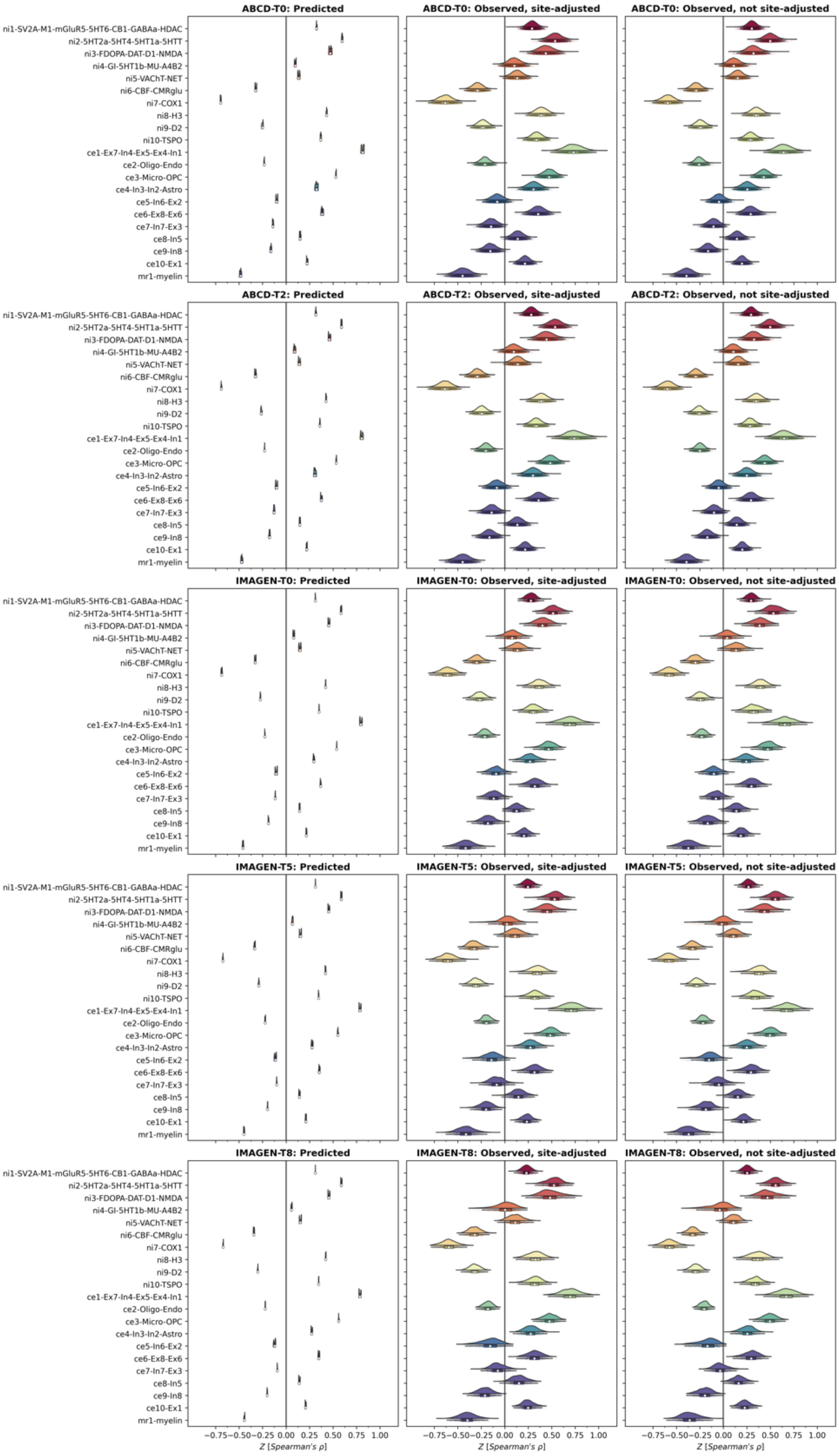
ABCD-T0: Predicted
ABCD-T0: Observed, site-adjusted
ABCD-T0: Observed, not site-adjusted
ABCD-T2: Predicted
ABCD-T2: Observed, site-adjusted
ABCD-T2: Observed, not site-adjusted
IMAGEN-T0: Predicted
IMAGEN-T0: Observed, site-adjusted
IMAGEN-T0: Observed, not site-adjusted
IMAGEN-T5: Predicted
IMAGEN-T5: Observed, site-adjusted
IMAGEN-T5: Observed, not site-adjusted
IMAGEN-T8: Predicted
IMAGEN-T8: Observed, site-adjusted
IMAGEN-T8: Observed, not site-adjusted
ni1-SV2A-M1-mGluR5-5HT6-CB1-GABAa-HDAC
ni2-5HT2a-5HT4-5HT1a-5HTT
ni3-FDOPA-DAT-D1-NMDA
ni4-GI-5HT1b-MU-A4B2
ni5-VAChT-NET
ni6-CBF-CMRglu
ni7-COX1
ni8-H3
ni9-D2
ni10-TSPO
ce1-Ex7-In4-Ex5-Ex4-In1
ce2-Oligo-Endo
ce3-Micro-OPC
ce4-In3-In2-Astro
ce5-In6-Ex2
ce6-Ex8-Ex6
ce7-In7-Ex3
ce8-In5
ce9-In8
ce10-Ex1
mr1-myelin
−0.75 −0.50 −0.25 0.00 0.25 0.50 0.75 1.00
Z [Spearman's ρ]

Fig. S14: Spatial colocalization between cross-sectional ABCD and IMAGEN cortical thickness data and multimodal brain systems

Spatial colocalization with multimodal predictors quantified as Z-transformed Spearman correlation for each ABCD and IMAGEN subject at each study time point (rows) based on CT data as predicted by the Braincharts model (first column), observed CT data after site-correction using the Braincharts model (second column), and original observed CT data without site-correction (third column). The latter serves the purpose to exclude potential overfitting effects due to the baseline ABCD data being used in estimation of the Braincharts model. See also the trajectory plots (Fig. 2 and Fig. S5

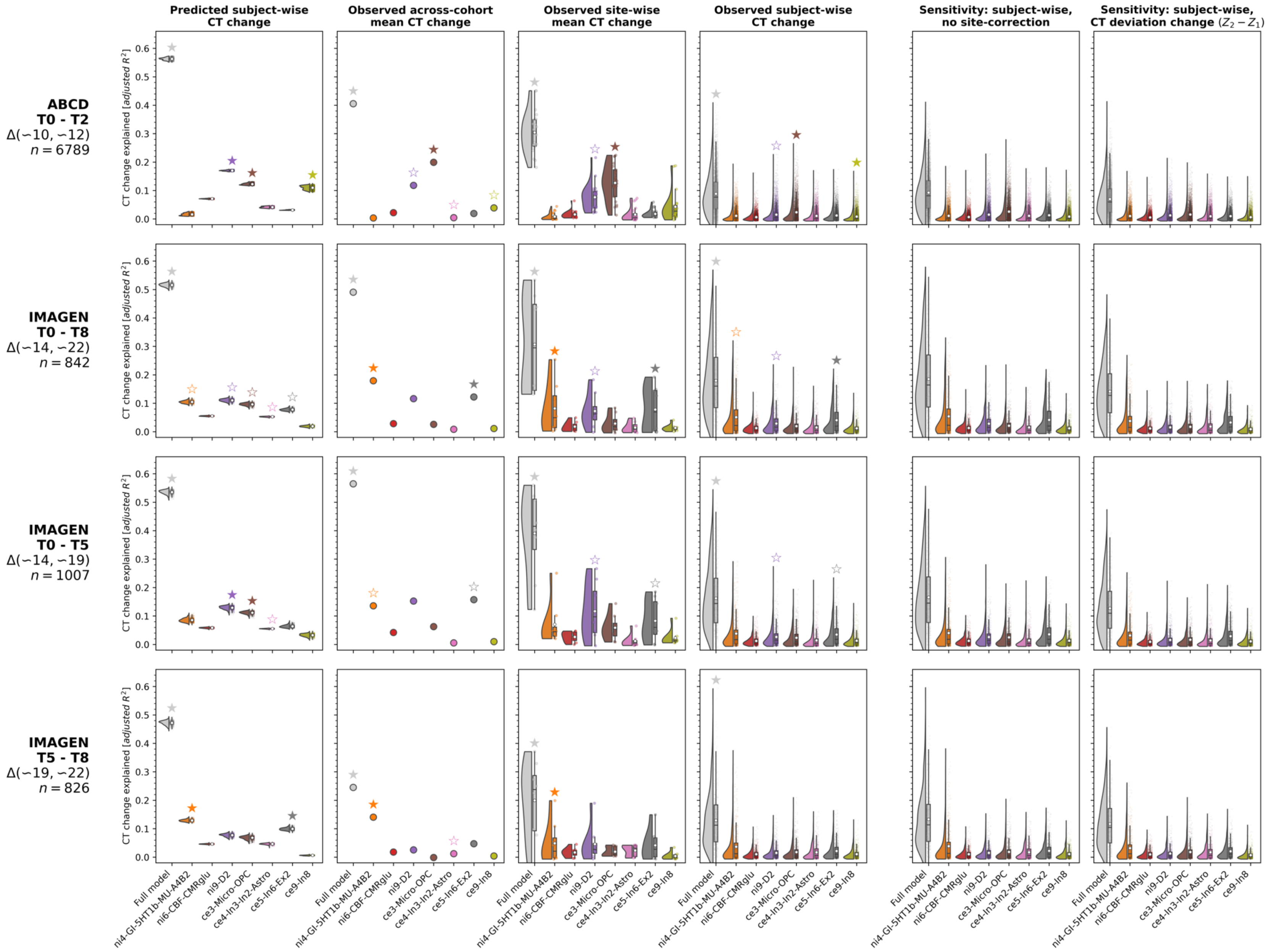

Predicted subject-wise CT change
Observed across-cohort mean CT change
Observed site-wise mean CT change
Observed subject-wise CT change
Sensitivity: subject-wise, no site-correction
Sensitivity: subject-wise, CT deviation change $(Z_2 - Z_1)$
ABCD
T0 - T2
Δ(~10, ~12)
$n = 6789$
IMAGEN
T0 - T8
Δ(~14, ~22)
$n = 842$
IMAGEN
T0 - T5
Δ(~14, ~19)
$n = 1007$
IMAGEN
T5 - T8
Δ(~19, ~22)
$n = 826$
CT change explained [adjusted R^2]
Full model
ni4-GI-5HT1b-MU-A4B2
ni6-CBF-CMRglu
ni9-D2
ce3-Micro-OPC
ce4-In3-In2-Astro
ce5-In6-Ex2
ce9-In8

Fig. S15: Detailed explained variance results of dominance analyses predicting ABCD and IMAGEN CT change from multilevel brain systems

Prediction of ABCD and IMAGEN CT change across four study time periods (rows) in different analysis settings. Raincloud plots and/or scatters show distributions of R^2 values resulting from cohort- or subject-wise dominance analyses. The leftmost grey elements depict the full model explained variance, the rightsided colored elements show the predictor-wise estimated *total dominance* statistics. For each subject- or cohort-wise analysis, the sum of the predictor-wise R^2 values equals the full model value. Stars printed over the plot elements indicate statistical significance determined based on null distributions of R^2 values as estimated by rerunning regression analyses with predictor null maps (filled star = FDR-corrected within each analysis/panel).
Column 1: prediction result based on CT change as predicted by the Braincharts model. Column 2: average CT change across each cohort. Column 3: average CT change across each site within each cohort. Column 4: CT change in single subjects. Column 5 and 6: sensitivity analyses on subject-level data; (5) values without site-correction to estimate effects of overfitting, and (6) change between deviation Z scores as estimated by the normative model

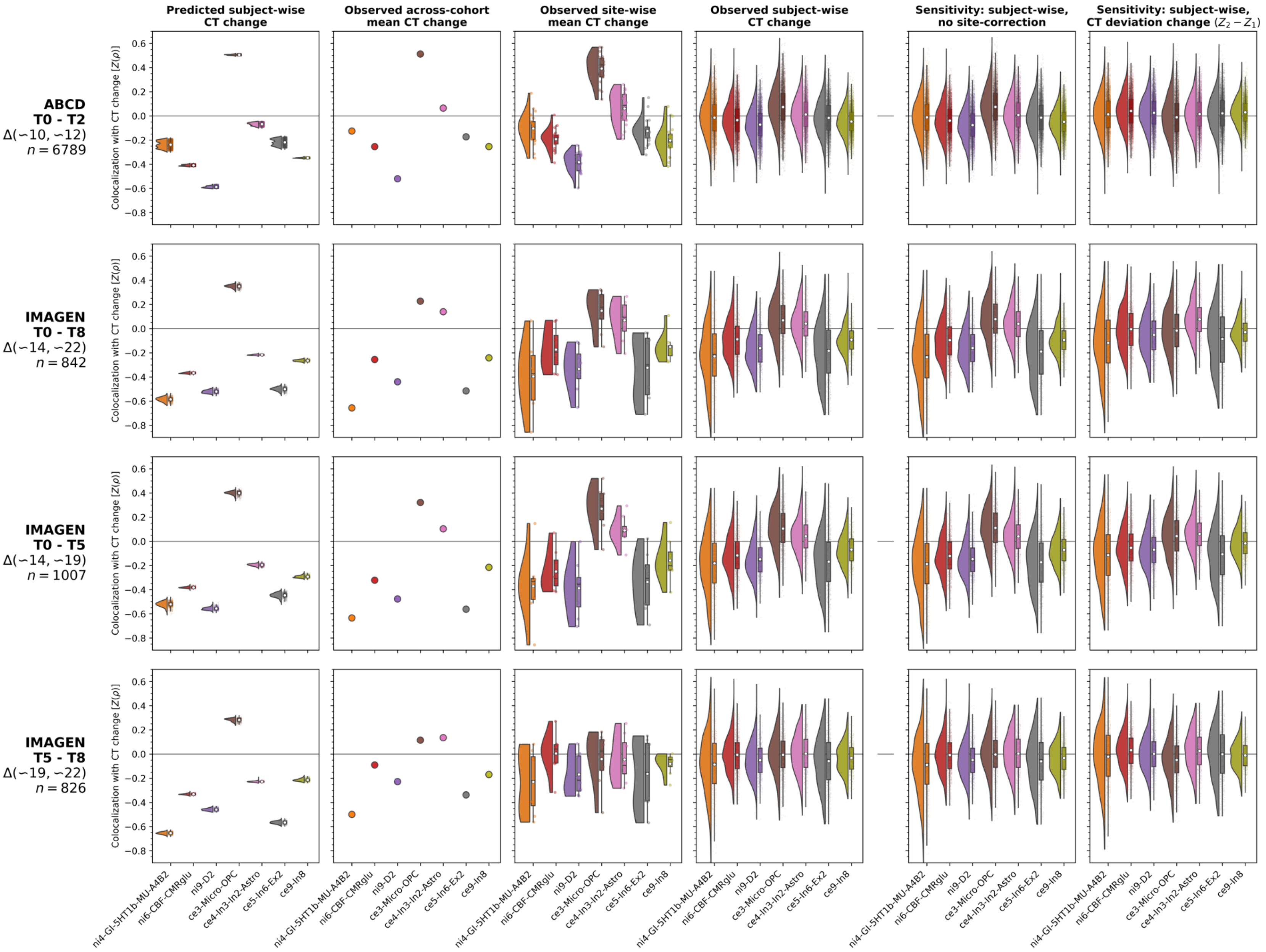

Predicted subject-wise CT change
Observed across-cohort mean CT change
Observed site-wise mean CT change
Observed subject-wise CT change
Sensitivity: subject-wise, no site-correction
Sensitivity: subject-wise, CT deviation change $(Z_2 - Z_1)$
ABCD
T0 - T2
Δ(~10, ~12)
$n = 6789$
IMAGEN
T0 - T8
Δ(~14, ~22)
$n = 842$
IMAGEN
T0 - T5
Δ(~14, ~19)
$n = 1007$
IMAGEN
T5 - T8
Δ(~19, ~22)
$n = 826$
Colocalization with CT change [Z(ρ)]
0.6
0.4
0.2
0.0
−0.2
−0.4
−0.6
−0.8
ni4-GI-5HT1b-MU-A4B2
ni6-CBF-CMRglu
ni9-D2
ce3-Micro-OPC
ce4-In3-In2-Astro
ce5-In6-Ex2
ce9-In8

Fig. S16: Detailed Spearman correlation results showing colocalization patterns between ABCD and IMAGEN CT change and multimodal predictors

Add-on to Fig. S14. Instead of multivariate regression analyses, Spearman correlations were calculated, to capture the sign of the spatial associations. As of this demonstrational purpose, no significance tests were performed.

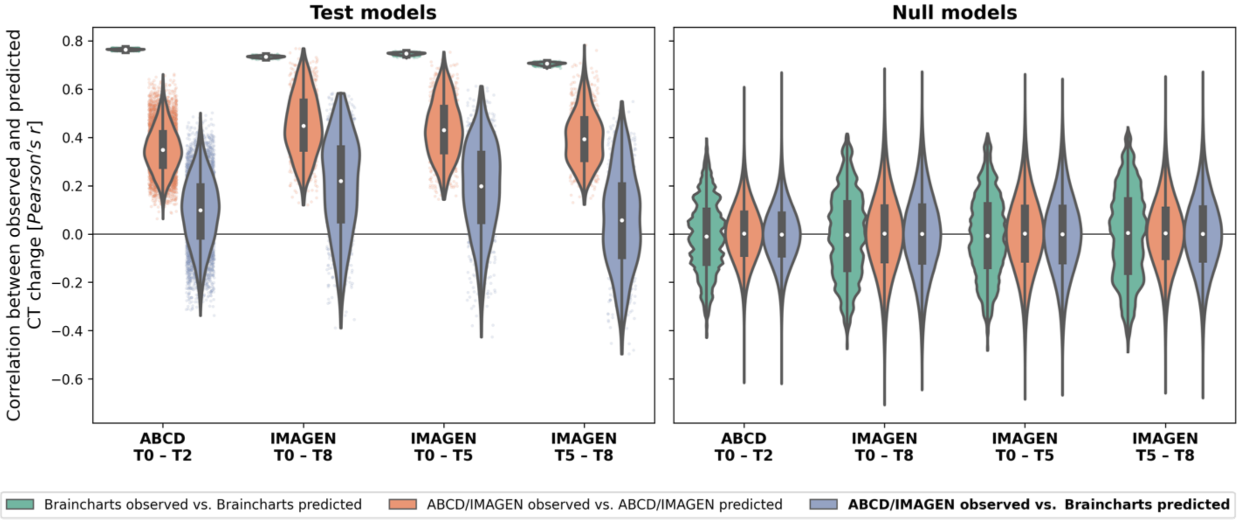

Fig. S17: Generalization of CT change prediction models trained on normative single-subject data to observed data

Evaluation of the generalizability of normative CT change prediction (= blue violins). The y-axis shows regression model fit as the Pearson correlation between observed and predicted responses (i.e., CT change patterns). The left panel shows analyses based on the actual biological system brain maps, the right panel shows results based on permuted maps (1,000 iterations). Green violins: Fit of regression models estimated on each subject's *normative* CT change patterns as predicted by the "Braincharts" model. Orange violins: Fit of regression models estimated on each subject's observed CT change patterns. **Blue violins:** Fit of models estimated on the *normative* CT change patterns but applied to the *observed* CT change. The result indicates how well the normative model, as the "population average", performs in representing each subject's CT associations to multilevel biological systems.

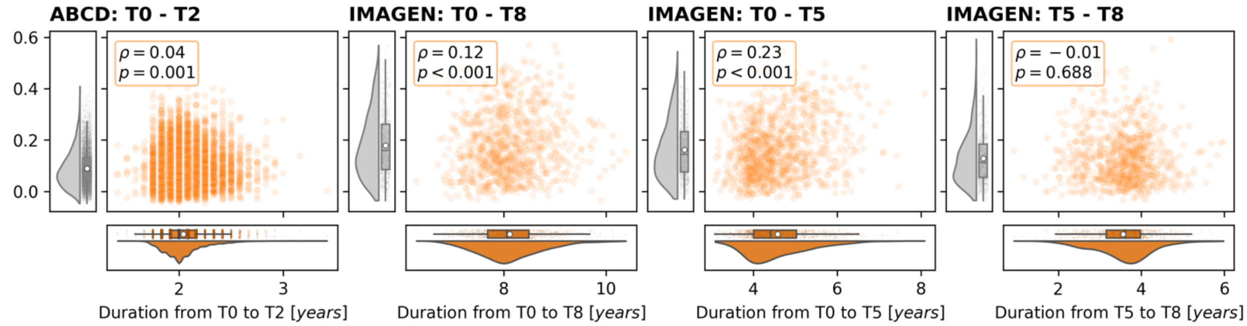

Fig. S18: Effects of follow-up duration on total explained cortical thickness change

Correlations between folluw-up duration for each dataset and at each time period (x axes) with total explained CT change variance (y axes). Boxes in the upper corners of each panel contain Spearman's rho and the associated parametric *p* value for each bivariate association.

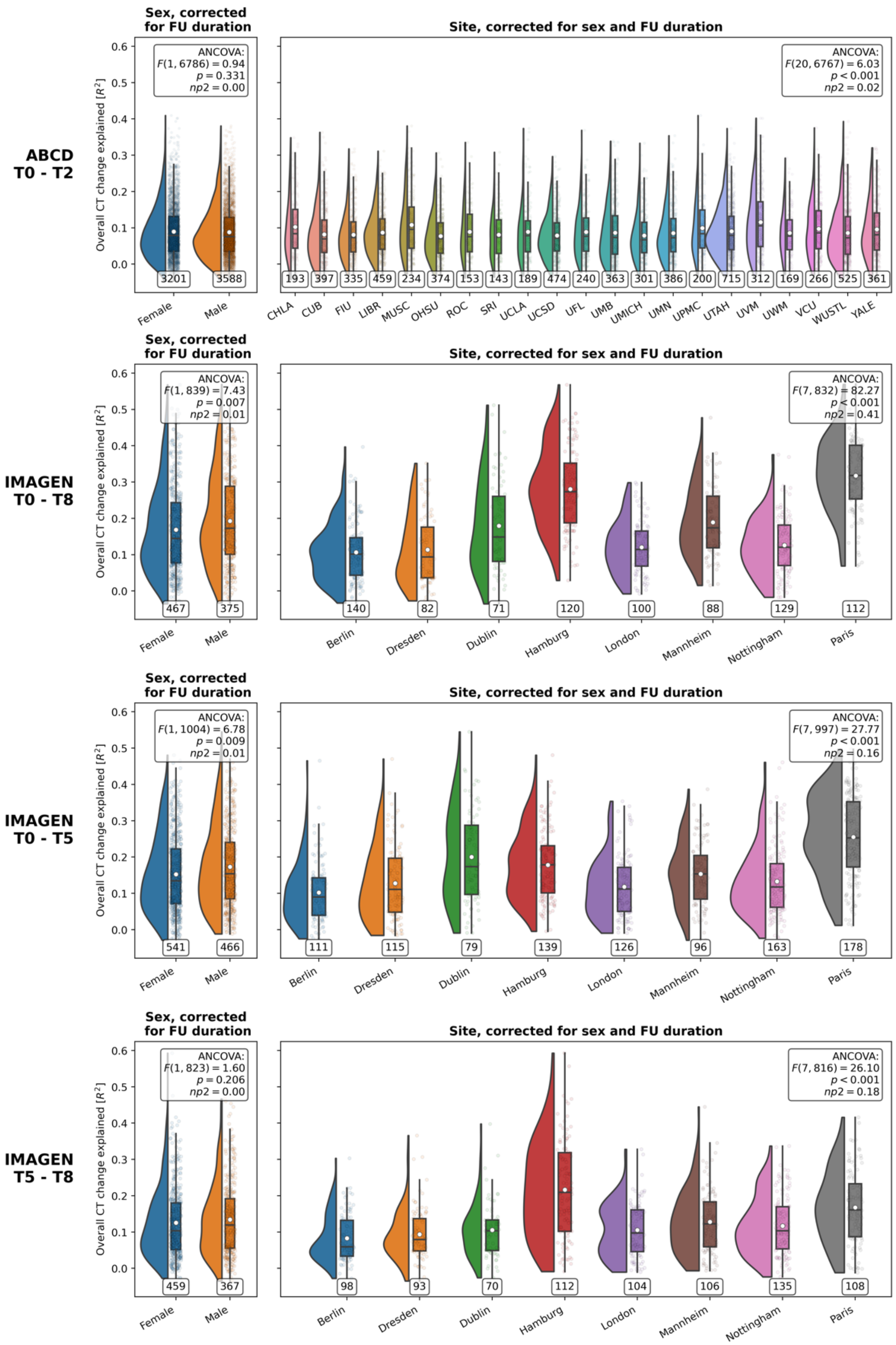

Fig. S19: Effects of sex and site on total explained cortical thickness change variance

Comparisons testing if the overall extent to which ABCD and IMAGEN CT change was explained (y axes) across each tested time period (rows) varied by binary sex (column 1) and site (column 2). Raincloud plot elements as described above. Sex was compared using T tests with Welch's correction, effect sizes are expressed as Hedge's *g*. Sites were compared using analyses of covariance (ANCOVAs) assessing the effect of site on explained CT change variance, including binary sex as covariate. Effect sizes are expressed as eta-squared (np2).

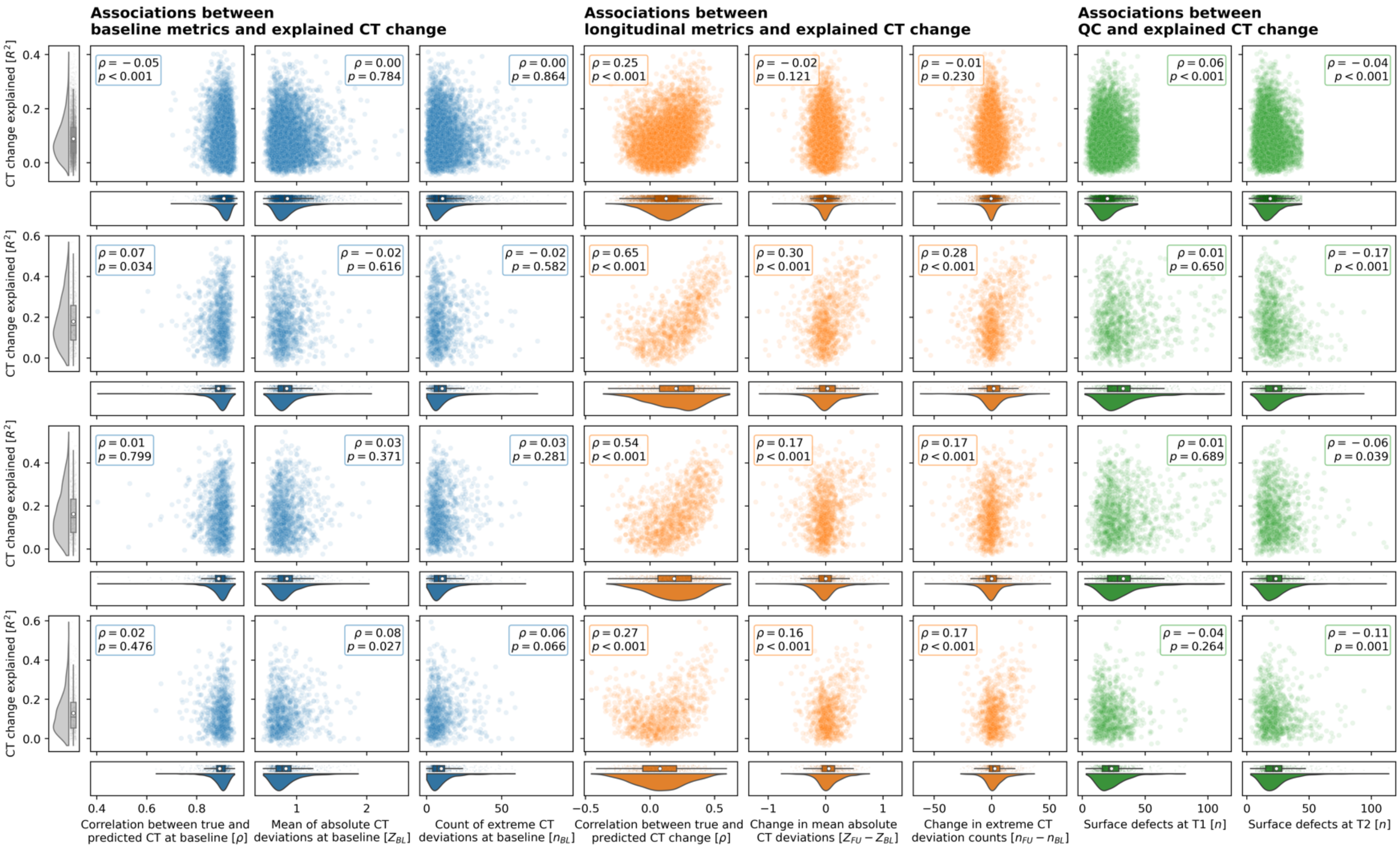

Fig. S20: Effects of predictive performance, subject-level atypical development, and surface reconstruction quality on explained cortical thickness

Scatterplots show relationships between cohort- and time period-wise (rows) total explained CT change (y axes) as estimated in subject-wise dominance analyses and on the x axes: Columns 1 and 4: Model fit; correlation between observed and predicted baseline CT or CT change. Columns 2 and 5: CT deviations; absolute CT deviation Z scores or their longitudinal change. Columns 3 and 6: CT deviations; counts of extreme deviations per subject or their longitudinal change (defined as deviation $Z > 2$). Columns 7 and 8: Data quality: Total Euler number, FreeSurfer's quality control metric for surface reconstruction. Blue plots indicate baseline metrics, orange plots indicate longitudinal metrics, green plots indicate associations with the Euler number at the first and second timepoint of each studied time period. Boxes in the upper corners of each panel contain Spearman's rho and the associated parametric p value for each bivariate association.

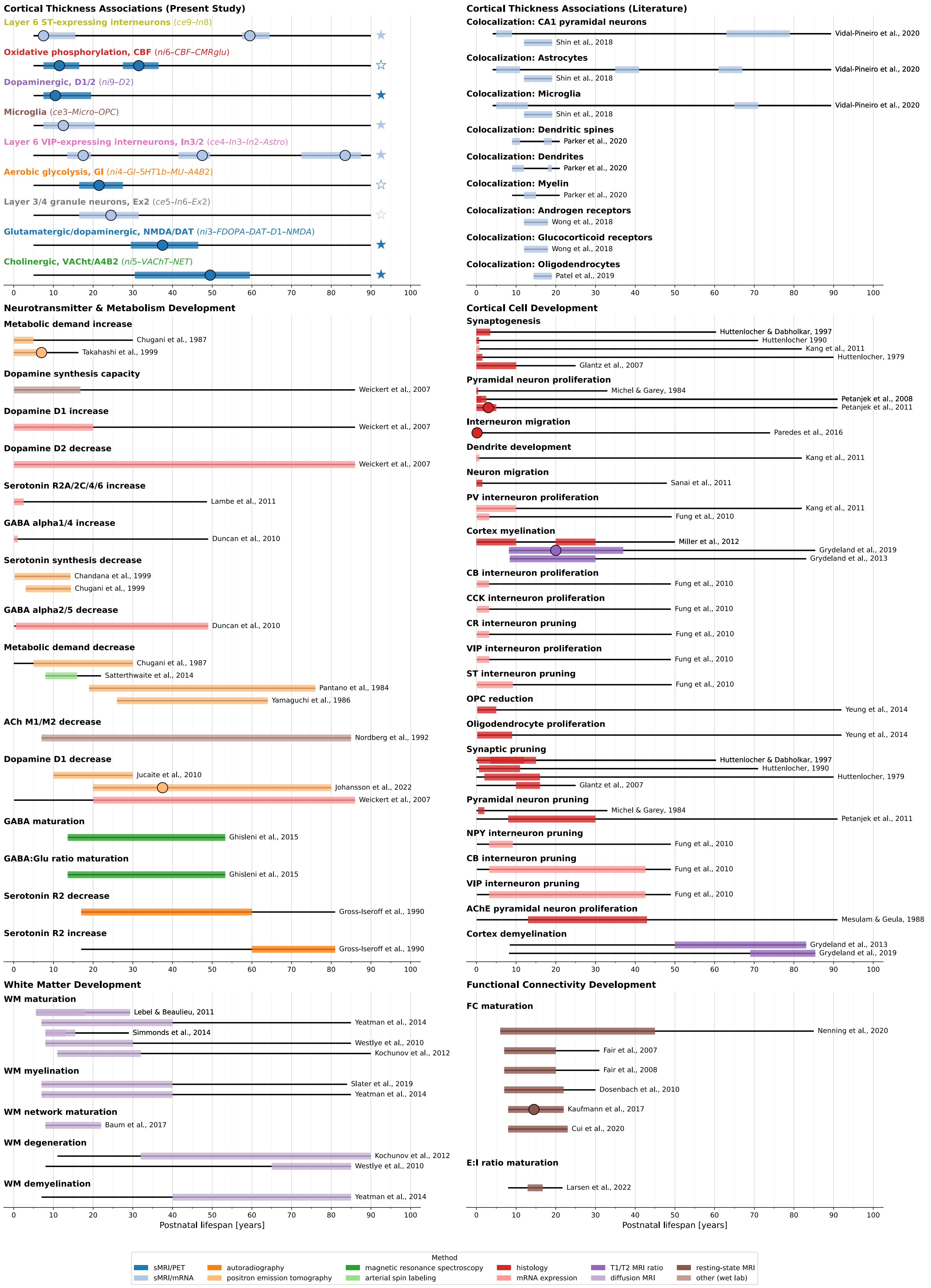
Cortical Thickness Associations (Present Study)
Layer 6 ST-expressing interneurons (ce9–In8)
Oxidative phosphorylation, CBF (ni6–CBF–CMRglu)
Dopaminergic, D1/2 (ni9–D2)
Microglia (ce3–Micro–OPC)
Layer 6 VIP-expressing interneurons, In3/2 (ce4–In3–In2–Astro)
Aerobic glycolysis, GI (ni4–GI–5HT1b–MU–A4B2)
Layer 3/4 granule neurons, Ex2 (ce5–In6–Ex2)
Glutamatergic/dopaminergic, NMDA/DAT (ni3–FDOPA–DAT–D1–NMDA)
Cholinergic, VAChT/A4B2 (ni5–VAChT–NET)
Cortical Thickness Associations (Literature)
Colocalization: CA1 pyramidal neurons
Vidal-Pineiro et al., 2020
Shin et al., 2018
Colocalization: Astrocytes
Vidal-Pineiro et al., 2020
Shin et al., 2018
Colocalization: Microglia
Vidal-Pineiro et al., 2020
Shin et al., 2018
Colocalization: Dendritic spines
Parker et al., 2020
Colocalization: Dendrites
Parker et al., 2020
Colocalization: Myelin
Parker et al., 2020
Colocalization: Androgen receptors
Wong et al., 2018
Colocalization: Glucocorticoid receptors
Wong et al., 2018
Colocalization: Oligodendrocytes
Patel et al., 2019
Neurotransmitter & Metabolism Development
Metabolic demand increase
Chugani et al., 1987
Takahashi et al., 1999
Dopamine synthesis capacity
Weickert et al., 2007
Dopamine D1 increase
Weickert et al., 2007
Dopamine D2 decrease
Weickert et al., 2007
Serotonin R2A/2C/4/6 increase
Lambe et al., 2011
GABA alpha1/4 increase
Duncan et al., 2010
Serotonin synthesis decrease
Chandana et al., 1999
Chugani et al., 1999
GABA alpha2/5 decrease
Duncan et al., 2010
Metabolic demand decrease
Chugani et al., 1987
Satterthwaite et al., 2014
Pantano et al., 1984
Yamaguchi et al., 1986
ACh M1/M2 decrease
Nordberg et al., 1992
Dopamine D1 decrease
Jucaite et al., 2010
Johansson et al., 2022
Weickert et al., 2007
GABA maturation
Ghisleni et al., 2015
GABA:Glu ratio maturation
Ghisleni et al., 2015
Serotonin R2 decrease
Gross-Iseroff et al., 1990
Serotonin R2 increase
Gross-Iseroff et al., 1990
Cortical Cell Development
Synaptogenesis
Huttenlocher & Dabholkar, 1997
Huttenlocher 1990
Kang et al., 2011
Huttenlocher, 1979
Glantz et al., 2007
Pyramidal neuron proliferation
Michel & Garey, 1984
Petanjek et al., 2008
Petanjek et al., 2011
Interneuron migration
Paredes et al., 2016
Dendrite development
Kang et al., 2011
Neuron migration
Sanai et al., 2011
PV interneuron proliferation
Kang et al., 2011
Fung et al., 2010
Cortex myelination
Miller et al., 2012
Grydeland et al., 2019
Grydeland et al., 2013
CB interneuron proliferation
Fung et al., 2010
CCK interneuron proliferation
Fung et al., 2010
CR interneuron pruning
Fung et al., 2010
VIP interneuron proliferation
Fung et al., 2010
ST interneuron pruning
Fung et al., 2010
OPC reduction
Yeung et al., 2014
Oligodendrocyte proliferation
Yeung et al., 2014
Synaptic pruning
Huttenlocher & Dabholkar, 1997
Huttenlocher, 1990
Huttenlocher, 1979
Glantz et al., 2007
Pyramidal neuron pruning
Michel & Garey, 1984
Petanjek et al., 2011
NPY interneuron pruning
Fung et al., 2010
CB interneuron pruning
Fung et al., 2010
VIP interneuron pruning
Fung et al., 2010
AChE pyramidal neuron proliferation
Mesulam & Geula, 1988
Cortex demyelination
Grydeland et al., 2013
Grydeland et al., 2019
White Matter Development
WM maturation
Lebel & Beaulieu, 2011
Yeatman et al., 2014
Simmonds et al., 2014
Westlye et al., 2010
Kochunov et al., 2012
WM myelination
Slater et al., 2019
Yeatman et al., 2014
WM network maturation
Baum et al., 2017
WM degeneration
Kochunov et al., 2012
Westlye et al., 2010
WM demyelination
Yeatman et al., 2014
Postnatal lifespan [years]
Functional Connectivity Development
FC maturation
Nenning et al., 2020
Fair et al., 2007
Fair et al., 2008
Dosenbach et al., 2010
Kaufmann et al., 2017
Cui et al., 2020
E:I ratio maturation
Larsen et al., 2022
Postnatal lifespan [years]
0 10 20 30 40 50 60 70 80 90 100
Method
sMRI/PET
sMRI/mRNA
autoradiography
positron emission tomography
magnetic resonance spectroscopy
arterial spin labeling
histology
mRNA expression
T1/T2 MRI ratio
diffusion MRI
resting-state MRI
other (wet lab)

Fig. S21: Summary of previous literature on processes involved in human brain development and maturation

Visualization of published results on correlates of human CT development and maturation. This collection is not exhaustive. The selection is limited to in vivo and postmortem studies in humans. The upper two panels are replicated from main Fig. 6 and are shown for completeness. Thin black lines depict the age range covered by each study. Thick colored bars show the time period in which an association between cortex measures and the respective study target was reported. If available, peaks of these assocations are marked by circles. Results are grouped by the broad area of research, colors code the applied methodology. All references can be found in the supplementary reference list (Chandana et al., 2005; D. C. Chugani et al., 1999; H. T. Chugani et al., 1987; Cui et al., 2020; Dosenbach et al., 2010; Duncan et al., 2010; Fair et al., 2007, 2008; Fung et al., 2010; Ghisleni et al., 2015; Glantz et al., 2007; Gross-Isseroff et al., 1990; Grydeland et al., 2013, 2019; Huttenlocher, 1979, 1990; Huttenlocher & Dabholkar, 1997; Johansson et al., 2022; Jucaite et al., 2010; Kang et al., 2011, 2011; Kaufmann et al., 2017; Kochunov et al., 2012; Lambe et al., 2011; Larsen et al., 2022; Lebel & Beaulieu, 2011; Mesulam & Geula, 1988; Michel & Garey, 1984, 1984; Miller et al., 2012; Nenning et al., 2020; Nordberg et al., 1992; Pantano et al., 1984; Paredes et al., 2016; Parker et al., 2020; Patel et al., 2019; Petanjek et al., 2008, 2011; Sanai et al., 2011; Satterthwaite et al., 2014; Shin et al., 2018; Simmonds et al., 2014; Slater et al., 2019; Takahashi et al., 1999; Vidal-Pineiro et al., 2020; Weickert et al., 2007; Westlye et al., 2010; Wong et al., 2018; Yamaguchi et al., 1986; Yeatman et al., 2014; Yeung et al., 2014).

4. Supplementary References

Aghourian, M., Legault-Denis, C., Soucy, J.-P., Rosa-Neto, P., Gauthier, S., Kostikov, A., Gravel, P., & Bédard, M.-A. (2017). Quantification of brain cholinergic denervation in Alzheimer's disease using PET imaging with [18F]-FEOBV. *Molecular Psychiatry*, *22*(11), 1531–1538. https://doi.org/10.1038/mp.2017.183

Arnatkevičiūtė, A., Fulcher, B. D., & Fornito, A. (2019). A practical guide to linking brain-wide gene expression and neuroimaging data. *NeuroImage*, *189*, 353–367. https://doi.org/10.1016/j.neuroimage.2019.01.011

Bayer, J. M. M., Dinga, R., Kia, S. M., Kottaram, A. R., Wolfers, T., Lv, J., Zalesky, A., Schmaal, L., & Marquand, A. (2021). *Accommodating site variation in neuroimaging data using normative and hierarchical Bayesian models* (p. 2021.02.09.430363). https://doi.org/10.1101/2021.02.09.430363

Beliveau, V., Ganz, M., Feng, L., Ozenne, B., Højgaard, L., Fisher, P. M., Svarer, C., Greve, D. N., & Knudsen, G. M. (2017). A High-Resolution In Vivo Atlas of the Human Brain's Serotonin System. *The Journal of Neuroscience*, *37*(1), 120–128. https://doi.org/10.1523/JNEUROSCI.2830-16.2016

Casey, B. J., Cannonier, T., Conley, M. I., Cohen, A. O., Barch, D. M., Heitzeg, M. M., Soules, M. E., Teslovich, T., Dellarco, D. V., Garavan, H., Orr, C. A., Wager, T. D., Banich, M. T., Speer, N. K., Sutherland, M. T., Riedel, M. C., Dick, A. S., Bjork, J. M., Thomas, K. M., … Dale, A. M. (2018). The Adolescent Brain Cognitive Development (ABCD) study: Imaging acquisition across 21 sites. *Developmental Cognitive Neuroscience*, *32*, 43–54. https://doi.org/10.1016/j.dcn.2018.03.001

Chandana, S. R., Behen, M. E., Juhász, C., Muzik, O., Rothermel, R. D., Mangner, T. J., Chakraborty, P. K., Chugani, H. T., & Chugani, D. C. (2005). Significance of abnormalities in developmental trajectory and asymmetry of cortical serotonin synthesis in autism. *International Journal of Developmental Neuroscience*, *23*(2–3), 171–182. https://doi.org/10.1016/j.ijdevneu.2004.08.002

Chugani, D. C., Muzik, O., Behen, M., Rothermel, R., Janisse, J. J., Lee, J., & Chugani, H. T. (1999). Developmental changes in brain serotonin synthesis capacity in autistic and nonautistic children. *Annals of Neurology*, *45*(3), 287–295. https://doi.org/10.1002/1531-8249(199903)45:3<287::aid-ana3>3.0.co;2-9

Chugani, H. T., Phelps, M. E., & Mazziotta, J. C. (1987). Positron emission tomography study of human brain functional development. *Annals of Neurology*, *22*(4), 487–497. https://doi.org/10.1002/ana.410220408

Cui, Z., Li, H., Xia, C. H., Larsen, B., Adebimpe, A., Baum, G. L., Cieslak, M., Gur, R. E., Gur, R. C., Moore, T. M., Oathes, D. J., Alexander-Bloch, A. F., Raznahan, A., Roalf, D. R., Shinohara, R. T., Wolf, D. H., Davatzikos, C., Bassett, D. S., Fair, D. A., … Satterthwaite, T. D. (2020). Individual Variation in Functional Topography of Association Networks in Youth. *Neuron*, *106*(2), 340-353.e8. https://doi.org/10.1016/j.neuron.2020.01.029

Destrieux, C., Fischl, B., Dale, A., & Halgren, E. (2010). Automatic parcellation of human cortical gyri and sulci using standard anatomical nomenclature. *NeuroImage*, *53*(1), 1–15. https://doi.org/10.1016/j.neuroimage.2010.06.010

Ding, Y.-S., Singhal, T., Planeta-Wilson, B., Gallezot, J.-D., Nabulsi, N., Labaree, D., Ropchan, J., Henry, S., Williams, W., Carson, R. E., Neumeister, A., & Malison, R. T. (2010). PET imaging of the effects of age and cocaine on the norepinephrine transporter in the human brain using (S,S)-[(11)C]O-methylreboxetine and HRRT. *Synapse (New York, N.Y.)*, *64*(1), 30–38. https://doi.org/10.1002/syn.20696

Dosenbach, N. U. F., Nardos, B., Cohen, A. L., Fair, D. A., Power, J. D., Church, J. A., Nelson, S. M., Wig, G. S., Vogel, A. C., Lessov-Schlaggar, C. N., Barnes, K. A., Dubis, J. W., Feczko, E., Coalson, R. S., Pruett, J. R., Barch, D. M., Petersen, S. E., & Schlaggar, B. L. (2010). Prediction of Individual Brain Maturity Using fMRI. *Science*, *329*(5997), 1358–1361. https://doi.org/10.1126/science.1194144

Dukart, J., Holiga, Š., Chatham, C., Hawkins, P., Forsyth, A., McMillan, R., Myers, J., Lingford-Hughes, A. R., Nutt, D. J., Merlo-Pich, E., Risterucci, C., Boak, L., Umbricht, D., Schobel, S., Liu, T., Mehta, M. A., Zelaya, F. O., Williams, S. C., Brown, G., … Sambataro, F. (2018). Cerebral blood flow predicts differential neurotransmitter activity. *Scientific Reports*, *8*(1), 4074. https://doi.org/10.1038/s41598-018-22444-0

Duncan, C. E., Webster, M. J., Rothmond, D. A., Bahn, S., Elashoff, M., & Shannon Weickert, C. (2010). Prefrontal GABAA receptor α-subunit expression in normal postnatal human development and schizophrenia. *Journal of Psychiatric Research*, *44*(10), 673–681. https://doi.org/10.1016/j.jpsychires.2009.12.007

Fair, D. A., Cohen, A. L., Dosenbach, N. U. F., Church, J. A., Miezin, F. M., Barch, D. M., Raichle, M. E., Petersen, S. E., & Schlaggar, B. L. (2008). The maturing architecture of the brain's default network. *Proceedings of the National Academy of Sciences*, *105*(10), 4028–4032. https://doi.org/10.1073/pnas.0800376105

Fair, D. A., Dosenbach, N. U. F., Church, J. A., Cohen, A. L., Brahmbhatt, S., Miezin, F. M., Barch, D. M., Raichle, M. E., Petersen, S. E., & Schlaggar, B. L. (2007). Development of distinct control networks through segregation and integration. *Proceedings of the National Academy of Sciences*, *104*(33), 13507–13512. https://doi.org/10.1073/pnas.0705843104

Finnema, S. J., Nabulsi, N. B., Eid, T., Detyniecki, K., Lin, S.-F., Chen, M.-K., Dhaher, R., Matuskey, D., Baum, E., Holden, D., Spencer, D. D., Mercier, J., Hannestad, J., Huang, Y., & Carson, R. E. (2016). Imaging synaptic density in the living human brain. *Science Translational Medicine*, *8*(348), 348ra96. https://doi.org/10.1126/scitranslmed.aaf6667

Fulcher, B. D., Little, M. A., & Jones, N. S. (2013). Highly comparative time-series analysis: The empirical structure of time series and their methods. *Journal of The Royal Society Interface*, *10*(83), 20130048. https://doi.org/10.1098/rsif.2013.0048

Fung, S. J., Webster, M. J., Sivagnanasundaram, S., Duncan, C., Elashoff, M., & Weickert, C. S. (2010). Expression of Interneuron Markers in the Dorsolateral Prefrontal Cortex of the Developing Human and in Schizophrenia. *American Journal of Psychiatry*, *167*(12), 1479–1488. https://doi.org/10.1176/appi.ajp.2010.09060784

Gallezot, J.-D., Nabulsi, N., Neumeister, A., Planeta-Wilson, B., Williams, W. A., Singhal, T., Kim, S., Maguire, R. P., McCarthy, T., Frost, J. J., Huang, Y., Ding, Y.-S., & Carson, R. E. (2010). Kinetic modeling of the serotonin 5-HT(1B) receptor radioligand [(11)C]P943 in humans. *Journal of Cerebral Blood Flow and Metabolism: Official Journal of the International Society of Cerebral Blood Flow and Metabolism*, *30*(1), 196–210. https://doi.org/10.1038/jcbfm.2009.195

Gallezot, J.-D., Planeta, B., Nabulsi, N., Palumbo, D., Li, X., Liu, J., Rowinski, C., Chidsey, K., Labaree, D., Ropchan, J., Lin, S.-F., Sawant-Basak, A., McCarthy, T. J., Schmidt, A. W., Huang, Y., & Carson, R. E. (2017). Determination of receptor occupancy in the presence of mass dose: [11C]GSK189254 PET imaging of histamine H3 receptor occupancy by PF-03654746. *Journal of Cerebral Blood Flow & Metabolism*, *37*(3), 1095–1107. https://doi.org/10.1177/0271678X16650697

Galovic, M., Al-Diwani, A., Vivekananda, U., Torrealdea, F., Erlandsson, K., Fryer, T. D., Hong, Y. T., Thomas, B. A., McGinnity, C. J., Edmond, E., Sander, K., Årstad, E., Jelcic, I., Aigbirhio, F. I., Groves, A. M., Thielemans, K., Hutton, B., Hammers, A., Duncan, J. S., … Investigators, for the N. (2021). *In vivo NMDA receptor function in people with NMDA receptor antibody encephalitis*. medRxiv. https://doi.org/1v

Ghisleni, C., Bollmann, S., Poil, S.-S., Brandeis, D., Martin, E., Michels, L., O'Gorman, R. L., & Klaver, P. (2015). Subcortical Glutamate Mediates the Reduction of Short-Range Functional Connectivity with Age in a Developmental Cohort. *Journal of Neuroscience*, *35*(22), 8433–8441. https://doi.org/10.1523/JNEUROSCI.4375-14.2015

Glantz, L. A., Gilmore, J. H., Hamer, R. M., Lieberman, J. A., & Jarskog, L. F. (2007). Synaptophysin and PSD-95 in the human prefrontal cortex from mid-gestation into early adulthood. *Neuroscience*, *149*(3), 582–591. https://doi.org/10.1016/j.neuroscience.2007.06.036

Gómez, F. J. G., Huertas, I., Ramírez, J. A. L., & Solís, D. G. (2018). Elaboración de una plantilla de SPM para la normalización de imágenes de PET con 18F-DOPA. *Imagen Diagnóstica*, *9*(01), Article 01. https://doi.org/10.33588/imagendiagnostica.901.2

Gross-Isseroff, R., Salama, D., Israeli, M., & Biegon, A. (1990). Autoradiographic analysis of age-dependent changes in serotonin 5-HT2 receptors of the human brain postmortem. *Brain Research*, *519*(1), 223–227. https://doi.org/10.1016/0006-8993(90)90081-L

Grydeland, H., Vértes, P. E., Váša, F., Romero-Garcia, R., Whitaker, K., Alexander-Bloch, A. F., Bjørnerud, A., Patel, A. X., Sederevičius, D., Tamnes, C. K., Westlye, L. T., White, S. R., Walhovd, K. B., Fjell, A. M., & Bullmore, E. T. (2019). Waves of Maturation and Senescence in Micro-structural MRI Markers of Human Cortical Myelination over the Lifespan. *Cerebral Cortex*, *29*(3), 1369–1381. https://doi.org/10.1093/cercor/bhy330

Grydeland, H., Walhovd, K. B., Tamnes, C. K., Westlye, L. T., & Fjell, A. M. (2013). Intracortical myelin links with performance variability across the human lifespan: Results from T1- and T2-weighted MRI myelin mapping and diffusion tensor imaging. *The Journal of Neuroscience: The Official Journal of the Society for Neuroscience*, *33*(47), 18618–18630. https://doi.org/10.1523/JNEUROSCI.2811-13.2013

Hawrylycz, M. J., Lein, E. S., Guillozet-Bongaarts, A. L., Shen, E. H., Ng, L., Miller, J. A., van de Lagemaat, L. N., Smith, K. A., Ebbert, A., Riley, Z. L., Abajian, C., Beckmann, C. F., Bernard, A., Bertagnolli, D., Boe, A. F., Cartagena, P. M., Chakravarty, M. M., Chapin, M., Chong, J., … Jones, A. R. (2012). An anatomically comprehensive atlas of the adult human brain transcriptome. *Nature*, *489*(7416), 391–399. https://doi.org/10.1038/nature11405

Hawrylycz, M. J., Miller, J. A., Menon, V., Feng, D., Dolbeare, T., Guillozet-Bongaarts, A. L., Jegga, A. G., Aronow, B. J., Lee, C.-K., Bernard, A., Glasser, M. F., Dierker, D. L., Menche, J., Szafer, A., Collman, F., Grange, P., Berman, K. A., Mihalas, S., Yao, Z., … Lein, E. (2015). Canonical genetic signatures of the adult human brain. *Nature Neuroscience*, *18*(12), Article 12. https://doi.org/10.1038/nn.4171

Hillmer, A. T., Esterlis, I., Gallezot, J. D., Bois, F., Zheng, M. Q., Nabulsi, N., Lin, S. F., Papke, R. L., Huang, Y., Sabri, O., Carson, R. E., & Cosgrove, K. P. (2016). Imaging of cerebral α4β2* nicotinic acetylcholine receptors with (-)-[(18)F]Flubatine PET: Implementation of bolus plus constant infusion and sensitivity to acetylcholine in human brain. *NeuroImage*, *141*, 71–80. https://doi.org/10.1016/j.neuroimage.2016.07.026

Huttenlocher, P. R. (1979). Synaptic density in human frontal cortex—Developmental changes and effects of aging. *Brain Research*, *163*(2), 195–205. https://doi.org/10.1016/0006-8993(79)90349-4

Huttenlocher, P. R. (1990). Morphometric study of human cerebral cortex development. *Neuropsychologia*, *28*(6), 517–527. https://doi.org/10.1016/0028-3932(90)90031-I

Huttenlocher, P. R., & Dabholkar, A. S. (1997). Regional differences in synaptogenesis in human cerebral cortex. *The Journal of Comparative Neurology*, *387*(2), 167–178. https://doi.org/10.1002/(sici)1096-9861(19971020)387:2<167::aid-cne1>3.0.co;2-z

Johansson, J., Nordin, K., Pedersen, R., Karalija, N., Papenberg, G., Andersson, M., Korkki, S. M., Riklund, K., Guitart-Masip, M., Rieckmann, A., Bäckman, L., Nyberg, L., & Salami, A. (2022). *Bi-phasic patterns of age-related differences in dopamine D1 receptors across the adult lifespan* (p. 2022.05.24.493225). bioRxiv. https://doi.org/10.1101/2022.05.24.493225

Jucaite, A., Forssberg, H., Karlsson, P., Halldin, C., & Farde, L. (2010). Age-related reduction in dopamine D1 receptors in the human brain: From late childhood to adulthood, a positron emission tomography study. *Neuroscience*, *167*(1), 104–110. https://doi.org/10.1016/j.neuroscience.2010.01.034

Kaller, S., Rullmann, M., Patt, M., Becker, G.-A., Luthardt, J., Girbardt, J., Meyer, P. M., Werner, P., Barthel, H., Bresch, A., Fritz, T. H., Hesse, S., & Sabri, O. (2017). Test-retest measurements of dopamine D1-type receptors using simultaneous PET/MRI imaging. *European Journal of Nuclear Medicine and Molecular Imaging*, *44*(6), 1025–1032. https://doi.org/10.1007/s00259-017-3645-0

Kang, H. J., Kawasawa, Y. I., Cheng, F., Zhu, Y., Xu, X., Li, M., Sousa, A. M. M., Pletikos, M., Meyer, K. A., Sedmak, G., Guennel, T., Shin, Y., Johnson, M. B., Krsnik, Ž., Mayer, S., Fertuzinhos, S., Umlauf, S., Lisgo, S. N., Vortmeyer, A., … Šestan, N. (2011). Spatio-temporal transcriptome of the human brain. *Nature*, *478*(7370), Article 7370. https://doi.org/10.1038/nature10523

Kantonen, T., Karjalainen, T., Isojärvi, J., Nuutila, P., Tuisku, J., Rinne, J., Hietala, J., Kaasinen, V., Kalliokoski, K., Scheinin, H., Hirvonen, J., Vehtari, A., & Nummenmaa, L. (2020). Interindividual variability and lateralization of μ-opioid receptors in the human brain. *NeuroImage*, *217*, 116922. https://doi.org/10.1016/j.neuroimage.2020.116922

Kaufmann, T., Alnæs, D., Doan, N. T., Brandt, C. L., Andreassen, O. A., & Westlye, L. T. (2017). Delayed stabilization and individualization in connectome development are related to psychiatric disorders. *Nature Neuroscience*, *20*(4), 513–515. https://doi.org/10.1038/nn.4511

Kaulen, N., Rajkumar, R., Régio Brambilla, C., Mauler, J., Ramkiran, S., Orth, L., Sbaihat, H., Lang, M., Wyss, C., Rota Kops, E., Scheins, J., Neumaier, B., Ermert, J., Herzog, H., Langen, K.-J., Lerche, C., Shah, N. J., Veselinović, T., & Neuner, I. (2022). mGluR5 and GABAA receptor-specific parametric PET atlas construction—PET/MR data processing pipeline, validation, and application. *Human Brain Mapping*, *43*(7), 2148–2163. https://doi.org/10.1002/hbm.25778

Kim, M.-J., Lee, J.-H., Juarez Anaya, F., Hong, J., Miller, W., Telu, S., Singh, P., Cortes, M. Y., Henry, K., Tye, G. L., Frankland, M. P., Montero Santamaria, J. A., Liow, J.-S., Zoghbi, S. S., Fujita, M., Pike, V. W., & Innis, R. B. (2020). First-in-human evaluation of [11C]PS13, a novel PET radioligand, to quantify cyclooxygenase-1 in the brain. *European Journal of Nuclear Medicine and Molecular Imaging*, *47*(13), 3143–3151. https://doi.org/10.1007/s00259-020-04855-2

Kochunov, P., Williamson, D. E., Lancaster, J., Fox, P., Cornell, J., Blangero, J., & Glahn, D. C. (2012). Fractional anisotropy of water diffusion in cerebral white matter across the lifespan. *Neurobiology of Aging*, *33*(1), 9–20. https://doi.org/10.1016/j.neurobiolaging.2010.01.014

Lake, B. B., Ai, R., Kaeser, G. E., Salathia, N. S., Yung, Y. C., Liu, R., Wildberg, A., Gao, D., Fung, H.-L., Chen, S., Vijayaraghavan, R., Wong, J., Chen, A., Sheng, X., Kaper, F., Shen, R., Ronaghi, M., Fan, J.-B., Wang, W., … Zhang, K. (2016). Neuronal subtypes and diversity revealed by single-nucleus RNA sequencing of the human brain. *Science (New York, N.Y.)*, *352*(6293), 1586–1590. https://doi.org/10.1126/science.aaf1204

Lambe, E. K., Fillman, S. G., Webster, M. J., & Weickert, C. S. (2011). Serotonin Receptor Expression in Human Prefrontal Cortex: Balancing Excitation and Inhibition across Postnatal Development. *PLOS ONE*, *6*(7), e22799. https://doi.org/10.1371/journal.pone.0022799

Larsen, B., Cui, Z., Adebimpe, A., Pines, A., Alexander-Bloch, A., Bertolero, M., Calkins, M. E., Gur, R. E., Gur, R. C., Mahadevan, A. S., Moore, T. M., Roalf, D. R., Seidlitz, J., Sydnor, V. J., Wolf, D. H., & Satterthwaite, T. D. (2022). A developmental reduction of the excitation:inhibition ratio in association cortex during adolescence. *Science Advances*, *8*(5), eabj8750. https://doi.org/10.1126/sciadv.abj8750

Lebel, C., & Beaulieu, C. (2011). Longitudinal Development of Human Brain Wiring Continues from Childhood into Adulthood. *Journal of Neuroscience*, *31*(30), 10937–10947. https://doi.org/10.1523/JNEUROSCI.5302-10.2011

Lois, C., González, I., Izquierdo-García, D., Zürcher, N. R., Wilkens, P., Loggia, M. L., Hooker, J. M., & Rosas, H. D. (2018). Neuroinflammation in Huntington's Disease: New Insights with ^{11}C-PBR28 PET/MRI. *ACS Chemical Neuroscience*, *9*(11), 2563–2571. https://doi.org/10.1021/acschemneuro.8b00072

Markello, R. D., Arnatkeviciute, A., Poline, J.-B., Fulcher, B. D., Fornito, A., & Misic, B. (2021). Standardizing workflows in imaging transcriptomics with the abagen toolbox. *ELife*, *10*, e72129. https://doi.org/10.7554/eLife.72129

Markello, R. D., Hansen, J. Y., Liu, Z.-Q., Bazinet, V., Shafiei, G., Suárez, L. E., Blostein, N., Seidlitz, J., Baillet, S., Satterthwaite, T. D., Chakravarty, M. M., Raznahan, A., & Misic, B. (2022). neuromaps: Structural and functional interpretation of brain maps. *Nature Methods*, 1–8. https://doi.org/10.1038/s41592-022-01625-w

Mesulam, M.-M., & Geula, C. (1988). Acetylcholinesterase-rich pyramidal neurons in the human neocortex and hippocampus: Absence at birth, development during the life span, and dissolution in Alzheimer's disease. *Annals of Neurology*, *24*(6), 765–773. https://doi.org/10.1002/ana.410240611

Michel, A. E., & Garey, L. J. (1984). The development of dendritic spines in the human visual cortex. *Human Neurobiology*, *3*(4), 223–227.

Miller, D. J., Duka, T., Stimpson, C. D., Schapiro, S. J., Baze, W. B., McArthur, M. J., Fobbs, A. J., Sousa, A. M. M., Šestan, N., Wildman, D. E., Lipovich, L., Kuzawa, C. W., Hof, P. R., & Sherwood, C. C. (2012). Prolonged myelination in human neocortical evolution. *Proceedings of the National Academy of Sciences*, *109*(41), 16480–16485. https://doi.org/10.1073/pnas.1117943109

Naganawa, M., Nabulsi, N., Henry, S., Matuskey, D., Lin, S.-F., Slieker, L., Schwarz, A. J., Kant, N., Jesudason, C., Ruley, K., Navarro, A., Gao, H., Ropchan, J., Labaree, D., Carson, R. E., & Huang, Y. (2021). First-in-Human Assessment of 11C-LSN3172176, an M1 Muscarinic Acetylcholine Receptor PET Radiotracer. *Journal of Nuclear Medicine*, *62*(4), 553–560. https://doi.org/10.2967/jnumed.120.246967

Nenning, K.-H., Xu, T., Schwartz, E., Arroyo, J., Woehrer, A., Franco, A. R., Vogelstein, J. T., Margulies, D. S., Liu, H., Smallwood, J., Milham, M. P., & Langs, G. (2020). Joint embedding: A scalable alignment to compare individuals in a connectivity space. *NeuroImage*, *222*, 117232. https://doi.org/10.1016/j.neuroimage.2020.117232

Nordberg, A., Alafuzoff, I., & Winblad, B. (1992). Nicotinic and muscarinic subtypes in the human brain: Changes with aging and dementia. *Journal of Neuroscience Research*, *31*(1), 103–111. https://doi.org/10.1002/jnr.490310115

Normandin, M. D., Zheng, M.-Q., Lin, K.-S., Mason, N. S., Lin, S.-F., Ropchan, J., Labaree, D., Henry, S., Williams, W. A., Carson, R. E., Neumeister, A., & Huang, Y. (2015). Imaging the cannabinoid CB1 receptor in humans with [11C]OMAR: Assessment of kinetic analysis methods, test-retest reproducibility, and gender differences. *Journal of Cerebral Blood Flow and Metabolism: Official Journal of the International Society of Cerebral Blood Flow and Metabolism*, *35*(8), 1313–1322. https://doi.org/10.1038/jcbfm.2015.46

Pantano, P., Baron, J. C., Lebrun-Grandié, P., Duquesnoy, N., Bousser, M. G., & Comar, D. (1984). Regional cerebral blood flow and oxygen consumption in human aging. *Stroke*, *15*(4), 635–641. https://doi.org/10.1161/01.str.15.4.635

Paredes, M. F., James, D., Gil-Perotin, S., Kim, H., Cotter, J. A., Ng, C., Sandoval, K., Rowitch, D. H., Xu, D., McQuillen, P. S., Garcia-Verdugo, J.-M., Huang, E. J., & Alvarez-Buylla, A. (2016). Extensive migration of young neurons into the infant human frontal lobe. *Science*, *354*(6308), aaf7073. https://doi.org/10.1126/science.aaf7073

Parker, N., Patel, Y., Jackowski, A. P., Pan, P. M., Salum, G. A., Pausova, Z., Paus, T., & Saguenay Youth Study and the IMAGEN Consortium. (2020). Assessment of Neurobiological Mechanisms of Cortical Thinning During Childhood and Adolescence and Their Implications for Psychiatric Disorders. *JAMA Psychiatry*, *77*(11), 1127. https://doi.org/10.1001/jamapsychiatry.2020.1495

Patel, Y., Shin, J., Gowland, P. A., Pausova, Z., Paus, T., & IMAGEN consortium. (2019). Maturation of the Human Cerebral Cortex During Adolescence: Myelin or Dendritic Arbor? *Cerebral Cortex (New York, N.Y.: 1991)*, *29*(8), 3351–3362. https://doi.org/10/gqs4xc

Petanjek, Z., Judaš, M., Kostović, I., & Uylings, H. B. M. (2008). Lifespan Alterations of Basal Dendritic Trees of Pyramidal Neurons in the Human Prefrontal Cortex: A Layer-Specific Pattern. *Cerebral Cortex*, *18*(4), 915–929. https://doi.org/10.1093/cercor/bhm124

Petanjek, Z., Judaš, M., Šimić, G., Rašin, M. R., Uylings, H. B. M., Rakic, P., & Kostović, I. (2011). Extraordinary neoteny of synaptic spines in the human prefrontal cortex. *Proceedings of the National Academy of Sciences*, *108*(32), 13281–13286. https://doi.org/10.1073/pnas.1105108108

Quackenbush, J. (2002). Microarray data normalization and transformation. *Nature Genetics*, *32 Suppl*, 496–501. https://doi.org/10.1038/ng1032

Radhakrishnan, R., Nabulsi, N., Gaiser, E., Gallezot, J.-D., Henry, S., Planeta, B., Lin, S., Ropchan, J., Williams, W., Morris, E., D'Souza, D. C., Huang, Y., Carson, R. E., & Matuskey, D. (2018). Age-Related Change in 5-HT6 Receptor Availability in Healthy Male Volunteers Measured with 11C-GSK215083 PET. *Journal of Nuclear Medicine*, *59*(9), 1445–1450. https://doi.org/10.2967/jnumed.117.206516

Rutherford, S., Fraza, C., Dinga, R., Kia, S. M., Wolfers, T., Zabihi, M., Berthet, P., Worker, A., Verdi, S., Andrews, D., Han, L. K., Bayer, J. M., Dazzan, P., McGuire, P., Mocking, R. T., Schene, A., Sripada, C., Tso, I. F.,

Duval, E. R., … Marquand, A. F. (2022). Charting brain growth and aging at high spatial precision. *ELife*, *11*, e72904. https://doi.org/10.7554/eLife.72904

Rutherford, S., Kia, S. M., Wolfers, T., Fraza, C., Zabihi, M., Dinga, R., Berthet, P., Worker, A., Verdi, S., Ruhe, H. G., Beckmann, C. F., & Marquand, A. F. (2021). *The Normative Modeling Framework for Computational Psychiatry* (p. 2021.08.08.455583). https://doi.org/10.1101/2021.08.08.455583

Sanai, N., Nguyen, T., Ihrie, R. A., Mirzadeh, Z., Tsai, H.-H., Wong, M., Gupta, N., Berger, M. S., Huang, E., Garcia-Verdugo, J.-M., Rowitch, D. H., & Alvarez-Buylla, A. (2011). Corridors of migrating neurons in the human brain and their decline during infancy. *Nature*, *478*(7369), Article 7369. https://doi.org/10.1038/nature10487

Sandiego, C. M., Gallezot, J.-D., Lim, K., Ropchan, J., Lin, S., Gao, H., Morris, E. D., & Cosgrove, K. P. (2015). Reference region modeling approaches for amphetamine challenge studies with [11C]FLB 457 and PET. *Journal of Cerebral Blood Flow & Metabolism*, *35*(4), 623–629. https://doi.org/10.1038/jcbfm.2014.237

Satterthwaite, T. D., Shinohara, R. T., Wolf, D. H., Hopson, R. D., Elliott, M. A., Vandekar, S. N., Ruparel, K., Calkins, M. E., Roalf, D. R., Gennatas, E. D., Jackson, C., Erus, G., Prabhakaran, K., Davatzikos, C., Detre, J. A., Hakonarson, H., Gur, R. C., & Gur, R. E. (2014). Impact of puberty on the evolution of cerebral perfusion during adolescence. *Proceedings of the National Academy of Sciences*, *111*(23), 8643–8648. https://doi.org/10.1073/pnas.1400178111

Schumann, G., Loth, E., Banaschewski, T., Barbot, A., Barker, G., Büchel, C., Conrod, P. J., Dalley, J. W., Flor, H., Gallinat, J., Garavan, H., Heinz, A., Itterman, B., Lathrop, M., Mallik, C., Mann, K., Martinot, J.-L., Paus, T., Poline, J.-B., … Struve, M. (2010). The IMAGEN study: Reinforcement-related behaviour in normal brain function and psychopathology. *Molecular Psychiatry*, *15*(12), Article 12. https://doi.org/10.1038/mp.2010.4

Shin, J., French, L., Xu, T., Leonard, G., Perron, M., Pike, G. B., Richer, L., Veillette, S., Pausova, Z., & Paus, T. (2018). Cell-Specific Gene-Expression Profiles and Cortical Thickness in the Human Brain. *Cerebral Cortex*, *28*(9), 3267–3277. https://doi.org/10.1093/cercor/bhx197

Simmonds, D. J., Hallquist, M. N., Asato, M., & Luna, B. (2014). Developmental stages and sex differences of white matter and behavioral development through adolescence: A longitudinal diffusion tensor imaging (DTI) study. *NeuroImage*, *92*, 356–368. https://doi.org/10.1016/j.neuroimage.2013.12.044

Slater, D. A., Melie-Garcia, L., Preisig, M., Kherif, F., Lutti, A., & Draganski, B. (2019). Evolution of white matter tract microstructure across the life span. *Human Brain Mapping*, *40*(7), 2252–2268. https://doi.org/10.1002/hbm.24522

Smart, K., Cox, S. M. L., Scala, S. G., Tippler, M., Jaworska, N., Boivin, M., Séguin, J. R., Benkelfat, C., & Leyton, M. (2019). Sex differences in [11C]ABP688 binding: A positron emission tomography study of mGlu5 receptors. *European Journal of Nuclear Medicine and Molecular Imaging*, *46*(5), 1179–1183. https://doi.org/10.1007/s00259-018-4252-4

Takahashi, T., Shirane, R., Sato, S., & Yoshimoto, T. (1999). Developmental Changes of Cerebral Blood Flow and Oxygen Metabolism in Children. *AJNR: American Journal of Neuroradiology*, *20*(5), 917–922.

Vaishnavi, S. N., Vlassenko, A. G., Rundle, M. M., Snyder, A. Z., Mintun, M. A., & Raichle, M. E. (2010). Regional aerobic glycolysis in the human brain. *Proceedings of the National Academy of Sciences of the United States of America*, *107*(41), 17757–17762. https://doi.org/10.1073/pnas.1010459107

Vidal-Pineiro, D., Parker, N., Shin, J., French, L., Grydeland, H., Jackowski, A. P., Mowinckel, A. M., Patel, Y., Pausova, Z., Salum, G., Sørensen, Ø., Walhovd, K. B., Paus, T., & Fjell, A. M. (2020). Cellular correlates of cortical thinning throughout the lifespan. *Scientific Reports*, *10*(1), Article 1. https://doi.org/10.1038/s41598-020-78471-3

Weickert, C. S., Webster, M. J., Gondipalli, P., Rothmond, D., Fatula, R. J., Herman, M. M., Kleinman, J. E., & Akil, M. (2007). Postnatal alterations in dopaminergic markers in the human prefrontal cortex. *Neuroscience*, *144*(3), 1109–1119. https://doi.org/10.1016/j.neuroscience.2006.10.009

Westlye, L. T., Walhovd, K. B., Dale, A. M., Bjørnerud, A., Due-Tønnessen, P., Engvig, A., Grydeland, H., Tamnes, C. K., Østby, Y., & Fjell, A. M. (2010). Life-Span Changes of the Human Brain White Matter: Diffusion Tensor Imaging (DTI) and Volumetry. *Cerebral Cortex*, *20*(9), 2055–2068. https://doi.org/10.1093/cercor/bhp280

Wey, H.-Y., Gilbert, T. M., Zürcher, N. R., She, A., Bhanot, A., Taillon, B. D., Schroeder, F. A., Wang, C., Haggarty, S. J., & Hooker, J. M. (2016). Insights into neuroepigenetics through human histone deacetylase PET imaging. *Science Translational Medicine*, *8*(351), 351ra106. https://doi.org/10.1126/scitranslmed.aaf7551

Wong, A. P.-Y., French, L., Leonard, G., Perron, M., Pike, G. B., Richer, L., Veillette, S., Pausova, Z., & Paus, T. (2018). Inter-Regional Variations in Gene Expression and Age-Related Cortical Thinning in the Adolescent Brain. *Cerebral Cortex (New York, N.Y.: 1991)*, *28*(4), 1272–1281. https://doi.org/10.1093/cercor/bhx040

Yamaguchi, T., Kanno, I., Uemura, K., Shishido, F., Inugami, A., Ogawa, T., Murakami, M., & Suzuki, K. (1986). Reduction in regional cerebral metabolic rate of oxygen during human aging. *Stroke*, *17*(6), 1220–1228. https://doi.org/10.1161/01.str.17.6.1220

Yeatman, J. D., Wandell, B. A., & Mezer, A. A. (2014). Lifespan maturation and degeneration of human brain white matter. *Nature Communications*, *5*, 4932. https://doi.org/10.1038/ncomms5932

Yeung, M. S. Y., Zdunek, S., Bergmann, O., Bernard, S., Salehpour, M., Alkass, K., Perl, S., Tisdale, J., Possnert, G., Brundin, L., Druid, H., & Frisén, J. (2014). Dynamics of Oligodendrocyte Generation and Myelination in the Human Brain. *Cell*, *159*(4), 766–774. https://doi.org/10.1016/j.cell.2014.10.011
